# Studies of α′,β′‐Epoxyketone Synthesis by Small‐Molecule Flavins and Flavoenzymes

**DOI:** 10.1002/anie.202512568

**Published:** 2025-10-14

**Authors:** Alexandra Walter, Wolfgang Kuttenlochner, Wolfgang Eisenreich, Chengyang Yao, Michael Groll, Golo Storch

**Affiliations:** ^1^ Technical University of Munich (TUM) School of Natural Sciences and Catalysis Research Center (CRC) Lichtenbergstr. 4 85747 Garching Germany; ^2^ Technical University of Munich (TUM) School of Natural Sciences and Center for Protein Assemblies (CPA) Ernst‐Otto‐Fischer‐Straße 8 85748 Garching Germany; ^3^ Technical University of Munich (TUM) School of Natural Sciences and Bavarian NMR Center (BNMRZ), Structural Membrane Biochemistry Lichtenbergstr. 4 85747 Garching Germany

**Keywords:** Enzymes, Epoxyketones, Flavins, Inhibitors, Reaction mechanisms

## Abstract

Epoxomicin is a highly potent natural proteasome inhibitor and the structural scaffold for the anticancer drug carfilzomib. The biosynthesis of its α′,β′‐epoxyketone warhead involves the flavoenzyme EpxF, but a molecular understanding of the key catalytic reaction cascade remained elusive. Here, we disclose detailed mechanistic insights by characterizing all intermediates in the sequential steps of decarboxylation, desaturation, and epoxidation with synthetic flavins and the flavin‐dependent oxidoreductase EpxF. A high‐resolution crystal structure of EpxF revealed the architecture of the active site and enabled the identification of key catalytic residues. Exploratory docking based on this structure served as a qualitative tool to guide mutagenesis and rationalize substrate recognition. NMR studies with a ^13^C‐labeled epoxomicin precursor and structure‐based EpxF variants further supported the proposed mechanism. Our integrated approach revealed similarities between synthetic and natural flavin catalysts and offers avenues for developing sustainable biomimetic reactions.

## Graphical abstract

A combined synthetic and biochemical approach links the flavoenzyme EpxF to three key steps in epoxomicin biosynthesis. Crystallography, mutagenesis, and ^13^C‐labeling complete the mechanistic picture of α′,β′‐epoxyketone formation and illustrate the potential of flavin‐mediated transformations.

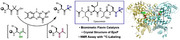

Proteasome inhibitors are key pharmaceutical agents in cancer therapy.^[^ ^1^, ^2^ ^]^ Carfilzomib **1**, marketed as Kyprolis, is a leading member of this class and was approved by the U.S. Food and Drug Administration in 2012 for the treatment of multiple myeloma.^[^ ^3^ ^]^ Among the many variations of the active pharmaceutical molecule are Oprozomib^[^ ^4^ ^]^ with improved oral bioavailability, and Zetomipzomib (KZR‐616),^[^ ^5^ ^]^ a candidate for treating rheumatic arthritis. The development of carfilzomib was inspired by the natural product epoxomicin^[^ ^2^, ^6^ ^]^ with both compounds featuring an epoxyketone warhead attached to a linear peptide (Figure 1a).^[^ ^7^ ^]^ Epoxomicin was originally isolated from the *Actinomycetes* strain *Goodfellowia coeruleoviolacea* ATCC 53 904 and identified as a potent antitumor agent due to its covalent binding to the trypsin‐like (β2) and chymotrypsin‐like (β5) catalytic sites of the proteasome core particle (CP).^[^ ^8^, ^9^ ^]^ Selective targeting of the CP over other proteases occurs via a two‐step reaction with the active site threonine (**3**).^[^ ^10^ ^]^ First, the side chain secondary alcohol (Thr1O^γ^) reversibly forms a hemiketal with the α′,β′‐epoxyketone warhead ketone. Epoxide ring‐opening with the amine *N*‐terminus (Figure 1b) then irreversibly binds the ligand to the CP. This bivalent reaction generates a 1,4‐oxazepane adduct (**4**), a peculiar covalent modification confirmed by crystallographic studies^[^ ^11^ ^]^ and investigated by computational analysis.^[^ ^12^ ^]^

**Figure 1:**
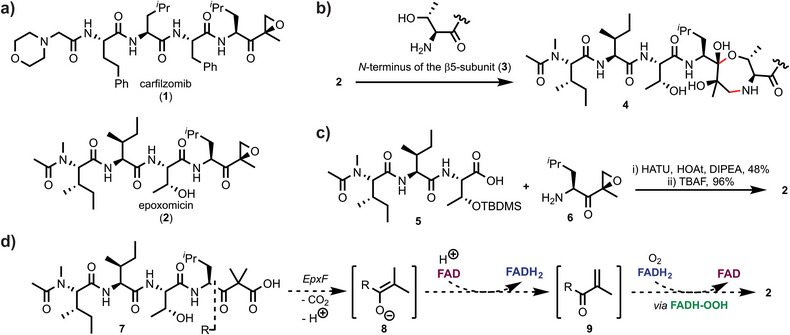
Origin and mode of action of epoxyketone proteasome inhibitors. a) Structures of carfilzomib (**1**) and epoxomicin (**2**). b) The epoxomicin warhead forms two new covalent bonds with the *N*‐terminal threonine of the 20S proteasome β5‐subunit. c) Key step of the chemical total synthesis of epoxomicin. d) The presumed biosynthetic key step is mediated by EpxF and relies on three consecutive reactions: Decarboxylation, desaturation, and epoxidation. TBDMS: *tert*‐Butyldimethylsilyl; HATU: Hexafluorophosphate azabenzotriazole tetramethyl uronium; HOAt: 1‐Hydroxy‐7‐azabenzotriazole; TBAF: tetra‐*n*‐Butylammonium fluoride; DIPEA: *N*,*N*‐Diisopropylethylamine.

Sin and colleagues first reported the total synthesis of epoxomicin, which relied on a seven‐step preparation of tripeptide **5**, followed by the coupling of epoxyketone **6** (Figure 1c).^[^ ^13^ ^]^ At the time, this approach provided access to epoxomicin analogs for biological studies since the natural biosynthetic route had not yet been elucidated. The corresponding biosynthetic gene clusters (BGCs) of epoxomicin^[^ ^14^, ^15^ ^]^ and of related epoxyketones such as eponemycin,^[^ ^16^ ^]^ macyranones,^[^ ^17^ ^]^ and epopromycines^[^ ^18^ ^]^ were identified only later and revealed a conserved assembly line of nonribosomal peptide and polyketide synthases. In contrast to chemical synthesis,^[^ ^13^, ^19^, ^20^ ^]^ the BGCs also encode an acyl‐CoA dehydrogenase, which installs the reactive epoxyketone warhead starting from the β‐ketoacid precursor **7**. Notably, homologous flavoenzymes are widely distributed across different epoxyketone pathways and consistently mediate the formation of the α′,β′‐epoxyketone pharmacophore from these precursors. In epoxomicin biosynthesis, this conversion is catalyzed by the flavoenzyme EpxF, which remains to be fully characterized. Studies of related enzymes, including TmcF (for TMC‐86A)^[^ ^21^ ^]^ and EpnF (for eponemycin),^[^ ^22^ ^]^ suggest that these oxidoreductases function by a flavin‐mediated dehydrogenase‐monooxygenase mechanism. If this pathway is operative, the reaction starts with decarboxylation of β‐ketoacid **7** to form enolate **8**, followed by oxidation to the unsaturated ketone **9** with flavin adenine dinucleotide (FAD) as the cosubstrate (Figure 1d). The final epoxidation step involves a flavin hydroperoxide [FADH‐OOH] intermediate,^[^ ^21^ ^]^ generated by the activation of O_2_ with FADH_2_.^[^ ^23^ ^]^ Despite these findings, none of the enzymes responsible for the conversion of β‐ketoacid precursors to the epoxyketone warhead have been structurally analyzed.

This study aims to decipher the reaction mechanism of EpxF through an integrated approach that combines organic chemistry, biosynthesis, structural analysis, mutagenesis, and catalysis. Specifically, we envision molecular insights into the elementary steps of epoxyketone formation by investigating small‐molecule flavin catalysts and characterizing structural features of EpxF. In contrast to the enzymatic reaction, where the entire series of events is orchestrated in the active site, the individual catalytic steps can be studied separately with molecular catalysts by changing the reaction conditions. Here, we elucidate similarities and differences between epoxomicin's chemical and enzymatic synthesis by validating and comparing each step of the reaction cascade.

The reaction cascade mediated by EpxF was first studied with a molecular flavin catalyst to probe for general feasibility and reaction intermediates. Leucine **10** was chosen as a model substrate for the biosynthetic precursor **7** since it is a derivative of the first amino acid in epoxomicin and contains the β‐ketoacid functionality. The compound slowly decarboxylates in solution, and the β‐ketoacid is transformed into isopropyl ketone **11**.^[^ ^21^ ^]^ This reaction proceeds via the enol intermediate, which tautomerizes. According to the proposed EpxF mechanism, the decarboxylation occurs in the active site and places the enol near the flavin cofactor, which initiates desaturation. In organic solvents, the substrate conversion is driven by photochemically excited flavin **12** (*E*_1/2_ (^3^ **12** ^*^/**12** ^•–^)  =  +1.35 V versus SCE^[^ ^24^ ^]^), which potentially also accelerates decarboxylation.^[^ ^25^, ^26^ ^]^ Indeed, isopropyl ketone **11** is only formed as a minor side product, and the oxidation led to hydroquinoid flavin **13** and unsaturated ketone **14** in 56% yield (Figure 2a), which confirms that the flavin‐mediated reaction occurs more rapidly than the competing thermal decarboxylation and tautomerization. Notably, both irradiation and the quinoid flavin were essential, as unproductive decarboxylation occurred in their absence (Figure 2b). Ester **15** remained inert since it cannot decarboxylate. The desaturation of leucine **10** to unsaturated ketone **14** was achieved catalytically using stoichiometric amounts of K_2_S_2_O_8_ to regenerate quinoid flavin **12** (Figure 2c). In contrast, no reactivity was detected without a catalyst.

**Figure 2:**
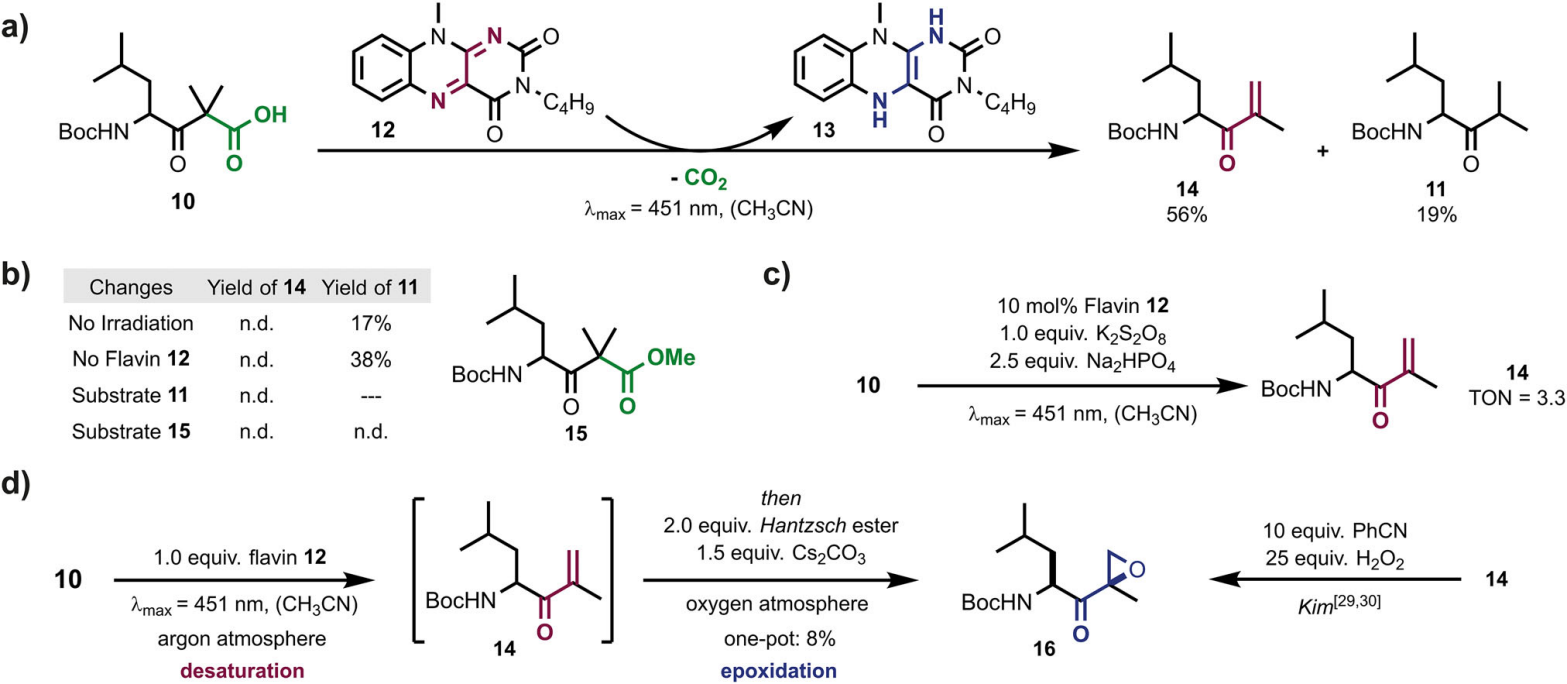
The flavin‐mediated sequential decarboxylation‐desaturation‐epoxidation reaction. a) Photochemical oxidation of β‐ketoacid **10**. b) Variations of the desaturation reaction conditions. 1.0 equiv. flavin **12**, (CH_3_CN), 3 h, *λ*_max_ = 451 nm. c) Catalytic desaturation with K_2_S_2_O_8_ as stoichiometric flavin oxidant. 10 mol% flavin **12**, (CH_3_CN), 8 h, *λ*_max_ = 451 nm, 33%. d) The desaturation‐epoxidation sequence leads to epoxyketone **16**. n.d.: No product formation was detected. Yields were determined relative to an internal NMR standard.

Next, leucine substrate **10** was subjected to a one‐pot multistep decarboxylation, oxidation, and epoxidation process with flavin **12**, closely mimicking the key steps of EpxF‐mediated epoxomicin biosynthesis. This approach was achieved by adjusting the reaction conditions following the formation of unsaturated ketone **14**. The argon atmosphere was replaced with oxygen, and *Hantzsch* ester was added as a flavin reductant, efficiently reducing O_2_ via a C4a hydroperoxide intermediate.^[^ ^27^ ^]^ As a result, the formation of epoxyketone **16** was unambiguously confirmed in this cascade, albeit in low yield due to susceptibility to ring‐opening^[^ ^28^ ^]^ and to decomposition during workup and purification (Figure 2d). A single diastereomer of product **16** was formed in the flavin‐mediated reaction. Its relative configuration was determined by comparison with the known stereochemical properties of the benzonitrile‐mediated epoxidation of unsaturated ketone **14**.^[^ ^29^, ^30^ ^]^ These results demonstrate that flavin catalysts are active in each step of the synthetic reaction sequence to convert β‐ketoacid substrates into α′,β′‐epoxyketones.

Having established a comprehensive characterization of all elementary steps of the flavin‐mediated epoxyketone synthesis, we next focused on elucidating the active site of EpxF, which catalyzes the analogous reaction in biosynthesis. Therefore, EpxF was heterologously expressed in *Escherichia coli* and purified using nickel affinity and size exclusion chromatography (Figure S1A). The crystal structure of EpxF solved at 2.4 Å resolution (PDB ID: 9GN5), reveals the typical topology of Acyl‐CoA dehydrogenases.^[^ ^31^ ^]^ Each subunit is composed of an *N*‐terminal α‐helical bundle, a seven‐stranded β‐sheet, and a *C*‐terminal α‐helical domain (Figure 3a). Structural comparison with the DALI server^[^ ^32^ ^]^ revealed significant similarities to other flavin‐dependent oxidoreductases despite low sequence identity at the amino acid level (<23%). Consistent with size exclusion chromatography data (Figure S1B), EpxF assembles into a homodimer with an interface of approximately 2500 Å^2^, further stabilized by interactions between the *C*‐terminal loops of the monomers. The 2F_o_‐F_c_ electron density map depicts FAD bound at two distinct catalytic centers located on opposite sides of the oxidoreductase (Figure 3a). Notably, the substrate binding loop (residues 253–264) and a flexible region (residues 336–346) are not defined in the electron density map. The adenine moiety of each catalytic FAD is anchored within the dimeric interface, involving residues Ile301, Tyr309, Leu306, Glu490, Gln412, and Phe411 (Figure 3b). The phosphate groups are coordinated via hydrogen bonds to Arg299 and the peptide main chain atoms of residues 156 to 158. The isoalloxazine ring of FAD is planar, consistent with its oxidized quinoid state, and engaged in π‐stacking with Phe227. The C2 carbonyl is positioned in a pocket formed by residues 149–152, while the C4 carbonyl oxygen interacts with the backbone NH of Gly185, and N5 is aligned with the backbone of His183 (Figure S2A).

**Figure 3:**
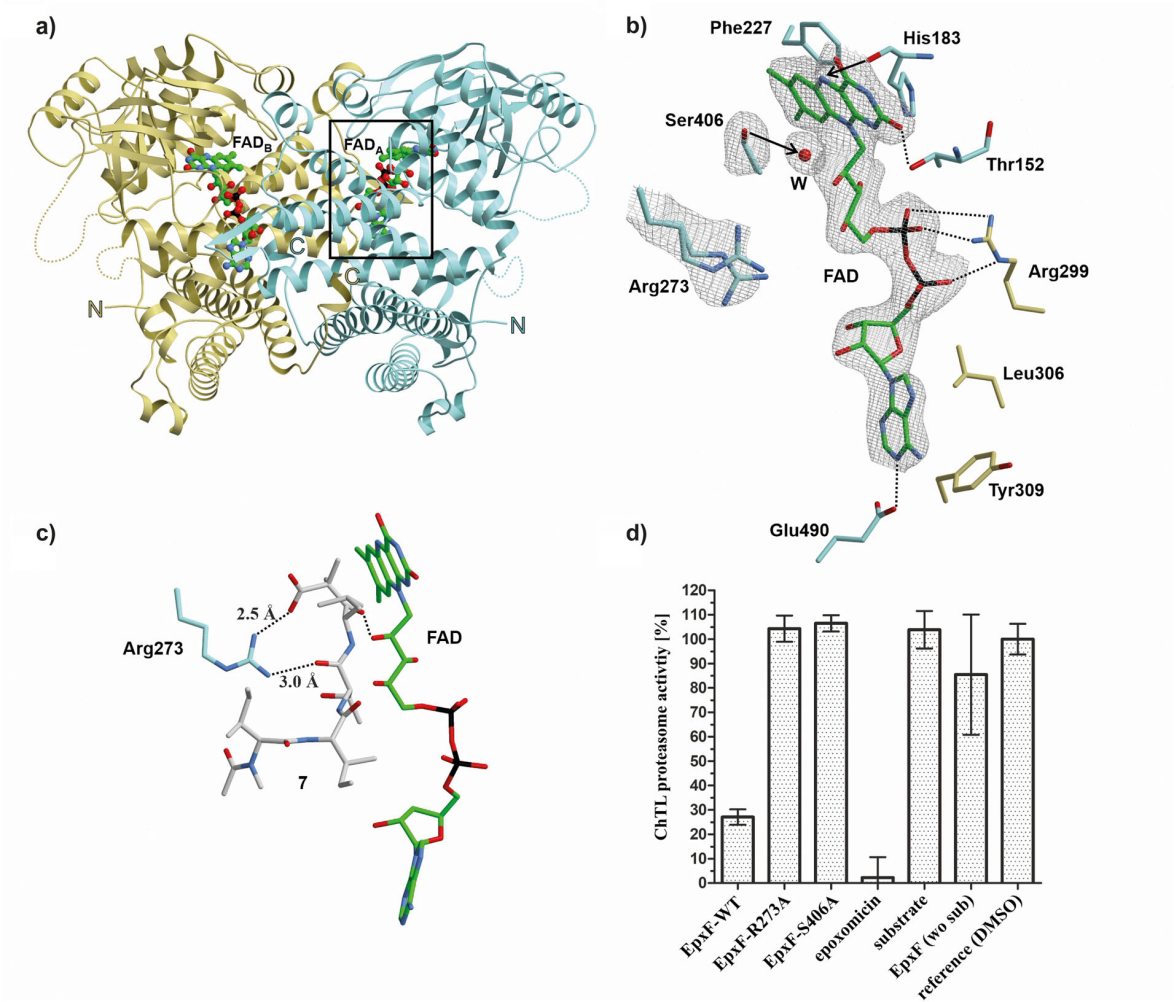
Crystal structure, ligand docking, and activity of EpxF. a) Cartoon representation of dimeric EpxF (PDB ID: 9GN5) with monomer A (blue) and monomer B (salmon), each binding an FAD cofactor (carbon in green). Arrows indicate the unresolved substrate binding loop (residues 253–264, dotted lines). b) Close‐up of the 2F_o_‐F_c_ electron density map (grey mesh, 1σ) showing active site residues (carbon in gold) and FAD in the dimeric interface, with dotted lines indicating H‐bonds. A functional water molecule (W) coordinates with Ser406 in proximity to the isoalloxazine C4a position. c) Predicted binding mode of substrate **7** (carbon in grey) with FAD, as determined by Autodock Vina.^[^ ^39^, ^40^ ^]^ d) Kinetic measurements of chymotrypsin‐like activity (ChTL) of yCP in EtOAc extracts with EpxF‐WT, EpxF‐R273A, and EpxF‐S406A in the presence of substrate. As a control, one reaction was carried out without the substrate (wo sub). Measurements were performed in triplicate, and results are presented as mean ± SEM, with values referenced to the DMSO control.

Beyond these FAD interactions, our crystallographic analysis of EpxF revealed a well‐defined water molecule located 4.1 Å from Ser406, 3.3 Å from the Gly407 backbone amide, and 3.6 Å from the C4a atom of the flavin (Figure 3b). This water molecule likely mimics the position of a C4a‐hydroperoxide and its geometry suggests that Ser406 may contribute to stabilizing such an intermediate. In contrast, an N5 hydroperoxide or N5 oxide would point away from Ser406 and thus appear less consistent with the observed arrangement.^[^ ^33^, ^34^, ^35^, ^36^, ^37^, ^38^ ^]^ To complement these structural observations and to obtain initial insights into substrate positioning during decarboxylation, we performed ligand docking with AutoDock Vina ^[^ ^39^, ^40^ ^]^ using the EpxF:FAD holo complex in a fixed conformation. The resulting model indicated a short hydrogen bond of 2.5 Å between Arg273 and the carboxylate group of β‐ketoacid **7** (Figures 3c and S2B), supporting that Arg273 contributes to productive substrate binding. However, given the flexibility of the substrate‐binding loop, this model was not interpreted quantitatively but rather used as a qualitative guide for designing subsequent mutagenesis experiments (see below).

To characterize the role of Arg273 and Ser406 in catalysis, a proteasome inhibitor assay was performed using the β‐ketoacid substrate **7** (see the Supporting Information for synthetic details). The ability of EpxF to synthesize epoxomicin was evaluated by in vitro fluorescence‐based kinetic measurements, specifically targeting the chymotrypsin‐like activity of the yeast 20S proteasome (yCP). Successful formation of epoxomicin inhibits yCP activity in this assay, providing a direct readout of enzymatic function. Wild‐type EpxF effectively catalyzed epoxomicin synthesis, resulting in complete inhibition of yCP as evidenced by the absence of a fluorescence signal. In contrast, the EpxF‐R273A and EpxF‐S406A variants failed to generate a functional proteasome inhibitor, leaving yCP activity unaffected (Figure 3d). Thus, Arg273 and Ser406 are essential residues for epoxomicin biosynthesis.

Next, we conducted a detailed mechanistic study of epoxomicin biosynthesis based on the molecular insights from the synthetic flavin‐mediated reaction sequence and EpxF active site analysis. NMR spectroscopy was employed to monitor enzyme catalysis and to identify reaction intermediates. Therefore, the ^13^C‐labeled β‐ketoester [^13^C_2_]**17** was synthesized from tripeptide **18** and the labeled leucine derivative [^13^C_2_]**15** with 58% yield (Figure 4a). Saponification resulted in a mixture of [^13^C_2_]**7** and *epi*‐[^13^C_2_]**7** due to epimerization adjacent to the carbonyl group.^[^ ^41^ ^]^ The ^13^C isotope labels enabled monitoring of EpxF‐mediated product formation by DEPT‐135 carbon NMR spectroscopy (Figure 4b, see the Supporting Information for details). Epoxomicin was identified by comparison to the authentic natural product using the oxirane methylene position (53.0 ppm, orange sphere) and the methyl group (16.1 ppm, blue sphere) as markers (Figure 4c). Notably, the unsaturated ketone intermediate [^13^C_2_]**9** was also detected through the characteristic olefinic methylene (128.0 ppm, rose sphere) and methyl group (16.9 ppm, green sphere) signals. Moreover, EpxF exclusively processed the β‐ketoacid substrate [^13^C_2_]**7** with (*S*)‐configuration, while *epi*‐[^13^C_2_]**7** remained unaffected. Control experiments (Figure 4d) in the absence of EpxF confirmed that neither epoxomicin [^13^C_2_]**2** nor unsaturated ketone [^13^C_2_]**9** were formed. Instead, partial decarboxylation of β‐ketoacids [^13^C_2_]**7** and *epi*‐[^13^C_2_]**7** to ketones [^13^C_2_]**19** and *epi*‐[^13^C_2_]**19** occurred non‐enzymatically (purple spheres).

**Figure 4:**
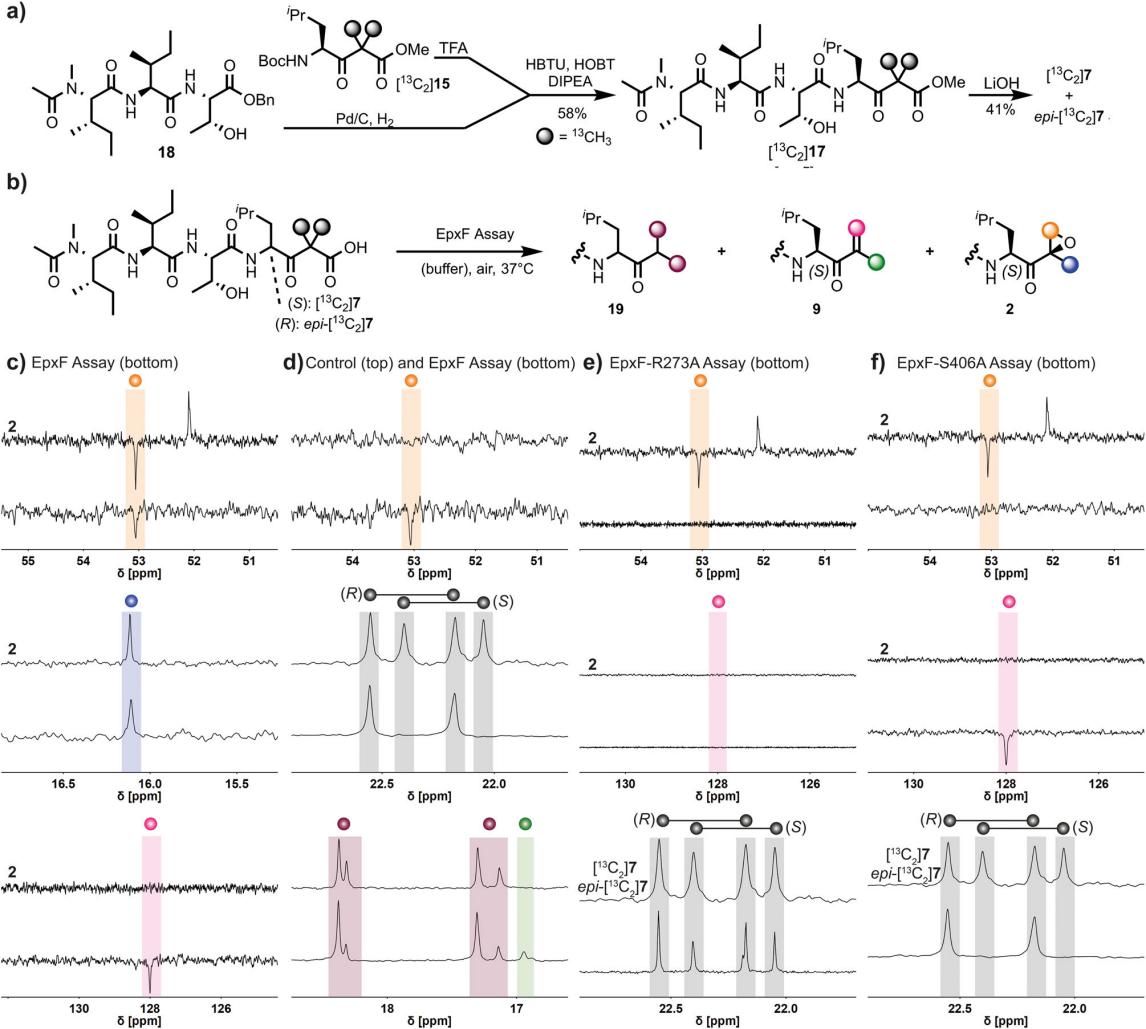
Flavoenzymatic formation of epoxomicin. a) Synthesis of the epoxomicin precursors [^13^C_2_]7 and *epi*‐[^13^C_2_]7. b) Enzymatic assay with EpxF. c) Overlay of DEPT‐135 carbon NMR spectra comparing EpxF assays with authentic epoxomicin 2, and d) a control reaction without EpxF. e) The EpxF‐R273A variant showed no conversion of [^13^C_2_]7 and *epi*‐[^13^C_2_]7. f) The assay with EpxF‐S406A demonstrated stereospecific conversion of [^13^C_2_]7 to the unsaturated intermediate [^13^C_2_]9, while *epi*‐[^13^C_2_]7 remained unaffected. TFA: Trifluoroacetic acid; HBTU: Hexafluorophosphate benzotriazole tetramethyl uronium; HOBt: Hydroxybenzotriazole.

Further NMR assays with the EpxF mutants R273A and S406A were performed to gain detailed insights into the sequential catalytic process of this sophisticated oxidoreductase. The R273A variant neither showed the formation of unsaturated ketone [^13^C_2_]**9**, epoxomicin [^13^C_2_]**2**, nor accelerated conversion of the natural substrate stereoisomer [^13^C_2_]**7** (Figure 4e). These results confirm that Arg273 is essential for substrate binding and decarboxylation. In contrast, the S406A variant demonstrated complete turnover of the natural stereoisomer of β‐ketoacid [^13^C_2_]**7** to intermediate [^13^C_2_]**9**, yet no epoxomicin [^13^C_2_]**2** formation was detected (Figure 4f). Thus, Ser406 is a key residue for the subsequent oxygenation step, likely through interaction with the flavin C4a hydroperoxide. In agreement with our activity assay (c.f. Figure 3d) and previous reports,^[^ ^42^ ^]^ the α,β‐unsaturated ketone [^13^C_2_]**9** does not inhibit the proteasome. Therefore, Arg273 is essential for the initial catalytic steps by coordinating the substrate for decarboxylation, while Ser406 is required to form the reactive flavin C4a hydroperoxide, which facilitates epoxidation.

Our study integrates organic synthesis, structural analysis, molecular catalysis, mutagenesis, activity assays, and isotopic labeling experiments to provide a comprehensive understanding of α′,β′‐epoxyketone synthesis. We identified the catalytic roles of key residues in EpxF by elucidating the individual steps of the flavin‐mediated cascade reaction, which opens up new avenues for discovering similar biosynthetic pathways in unrelated enzymes. From an organic synthesis perspective, the results provide a blueprint for translating complex enzymatic reactions into scalable processes in which stereoselectivity can be achieved via substrate control.^[^ ^43^ ^]^ Together, our findings demonstrate the synergy between structural insights into enzyme mechanisms and the strategic design of biomimetic organic reactions, highlighting the potential to advance catalytic transformations that are essential for the sustainable production of tailored organic molecules.

## Supporting Information

The crystallographic data has been deposited at the Protein Data Bank (PDB) under deposition number PDB ID: 9GN5. The authors have cited additional references within the Supporting Information.^[^ ^44^, ^45^, ^46^, ^47^, ^48^, ^49^, ^50^, ^51^, ^52^, ^53^, ^54^ ^]^

## Conflict of Interests

The authors declare no conflict of interest.

## Supporting information



Supporting Information

Supporting Information

Supporting Information

## Data Availability

The data that support the findings of this study are available in the Supporting Information of this article.
