# Supplementary material for: Studies of α′,β′‐Epoxyketone Synthesis by Small‐Molecule Flavins and Flavoenzymes
Source: Angew Chem Int Ed Engl. 2025 Oct 14;64(47):e202512568. doi: 10.1002/anie.202512568 (PMC12624316; doi:10.1002/anie.202512568)
Supplement: Supplementary file 1 — Supporting Information [file ANIE-64-e202512568-s003.pdf]

Supporting Information

Studies of α’,β’-Epoxyketone Synthesis by Small-Molecule Flavins and Flavoenzymes

Alexandra Walter,[a]‡ Wolfgang Kuttenlochner,[b]‡ Wolfgang Eisenreich,[c] Chengyang Yao,[b] Michael Groll,*[b] Golo Storch*[a]

[a] Technical University of Munich (TUM), School of Natural Sciences and Catalysis Research Center (CRC), Lichtenbergstr. 4, 85747 Garching, Germany.

[b] Technical University of Munich (TUM), School of Natural Sciences and Center for Protein Assemblies (CPA), Ernst-Otto-Fischer-Straße 8, 85748 Garching, Germany.

[c] Technical University of Munich (TUM), School of Natural Sciences and Bavarian NMR Center (BNMRZ), Structural Membrane Biochemistry, Lichtenbergstr. 4, 85747 Garching, Germany.

‡ These authors contributed equally: Alexandra Walter and Wolfgang Kuttenlochner

Table of Contents

Additional Figures and Tables 2
Materials and Methods 7
Synthetic Procedures 11
Saponification Study 30
Stoichiometric Flavin-Mediated Reactions 36
Catalytic Flavin-Mediated Reactions 43
NMR Spectra 49
References 75

Additional Figures and Tables

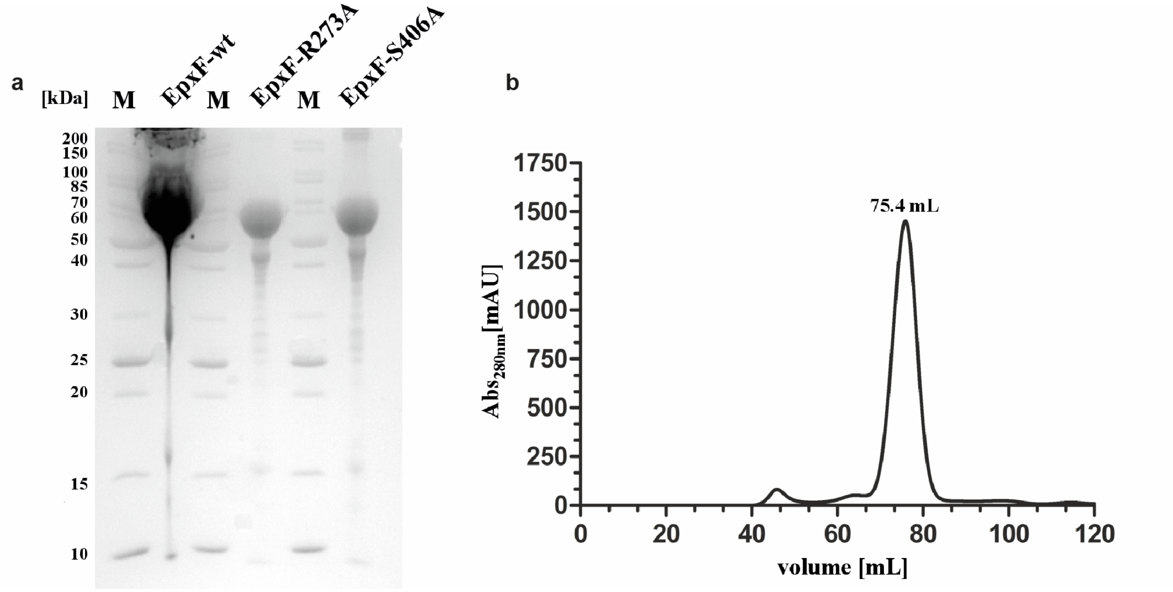

Figure S1. SDS-gel analysis and size exclusion chromatogram of EpxF. (a) Coomassie stained SDS-gel (15%) of EpxF, EpxF-R273A, and EpxF-S406A. 5 µg of purified protein were used for the analysis. (b) EpxF elutes as a dimer in size-exclusion chromatography using a HiLoad Superdex 200 prep grade column (Cytiva) indicated by a retention volume of 75.4 mL.

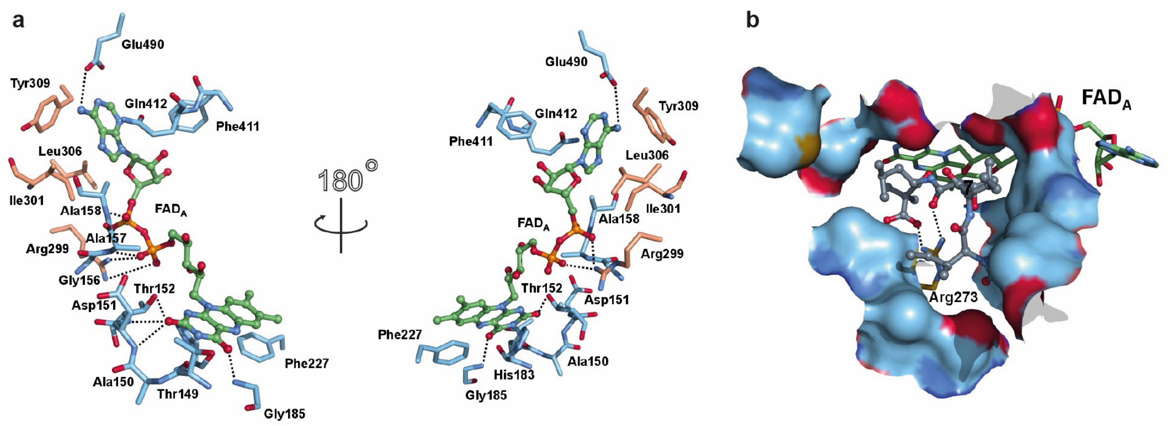

Figure S2. Detailed view of substrate binding in the active site of EpxF. (a) Detailed view of the cosubstrate binding site of EpxF. (b) Surface representation of the substrate binding channel interacting with docked **7** (formed by Thr149, Asp151, Ala157, Ala158, Met209, Val269, Ile270, Met276, Ser350, Gly408, Leu413, Trp416, and Ile420). Even though the interactions are reasonable, the substrate binding loop is absent in the electron density, presumably interacting with the substrate and altering its conformation. However, interactions near the catalytic active FAD cofactor proved valid in structure-based mutagenesis experiments

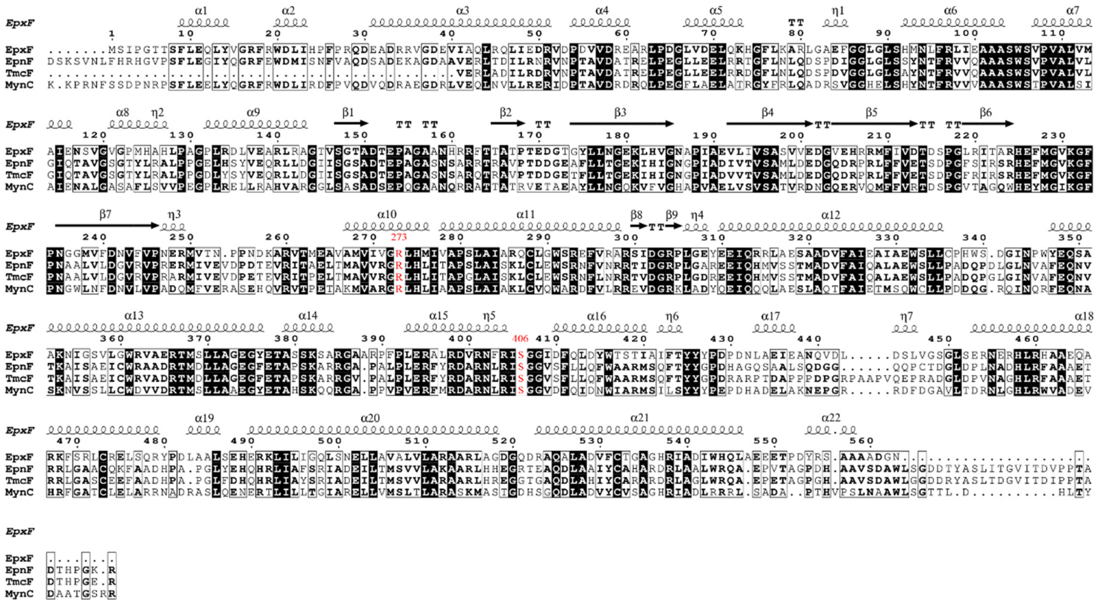

Figure S3. Primary sequence alignment of epoxyketone synthases. The primary sequence alignment of the epoxyketone synthases EpxF (GenBank: AHB38499.1), EpnF (GenBank: AHB38508.1), TmcF (GenBank: CUX96953.1), and MynC (NCBI Reference Sequence: WP_002626007.1) indicates strong overall homology and strict conservation of the catalytically active arginine and serine residues highlighted in red. Secondary structures are depicted according to the crystal structure of EpxF.[44]

Table S1. Oligonucleotides used in this study.

Oligonucleotide	Sequence 5' → 3'
EpxF_pET28b_for	GATTGGTGGATCCATTCCGGGGACAACCAGC
EpxF_pET28b_rev	CAGCCGCTGCGGATGGTAATTAACTGCAGATATCCATC
EpxF_R273A_for	CATTGTGGGGGCATTGCATATGATTGTAGCACCG
EpxF_R273A_rev	ACCATCGCAACGGCTTCC
EpxF_S406A_for	GGGAGGTATCGATTTCCAGCTCGATTATTG
EpxF_S406A_rev	GCAATACGGAAATTGCGCACATCG

Table S2. Crystallographic data collection and refinement statistics.

	EpxF:FAD
**Crystal parameters**	
Space group	C222
Cell constants	a = 131.2 Å<br>b = 147.9 Å<br>c = 67.2 Å
molecules / AU[a]	1
**Data collection**	
Beam line	X06SA, SLS
Wavelength (Å)	1.0
Resolution range (Å)[b]	30-2.4 (2.5-2.4)
No. observations	107236
No. unique reflections[c]	25881
Completeness (%)[b]	99.3 (99.8)
R_{merge} (%)[b, d]	6.2 (65.1)
I/s (I)[b]	15.3 (2.0)
**Refinement (REFMAC5)**	
Resolution range (Å)	30-2.4
No. refl. working set	24574
No. refl. test set	1293
No. non hydrogen	4314
No. of ligand atoms	53
Solvent molecules (H_2O, ions, buffer, FAD_{crys})	41
R_{work}/R_{free} (%)[e]	17.8/23.8
r.m.s.d. bond (Å) / angle (°)[f]	0.002/1.2
Average B-factor (Å^2)	61.8
Ramachandran Plot (%)[g]	96.0/3.8/0.2
PDB accession code	9GN5

[a] Asymmetric unit

[b] The values in parentheses for resolution range, completeness, R_{merge} and I/σ (I) correspond to the highest resolution shell

[c] Data reduction was carried out from a single crystal. Friedel pairs were treated as identical reflections

[d] $R_{merge}(I) = \Sigma_{hkl}\Sigma_j \mid I(hkl)_j - \langle I(hkl)\rangle \mid / \Sigma_{hkl} \Sigma_j I(hkl)_j$, where $I(hkl)_j$ is the jth measurement of the intensity of reflection hkl and <I(hkl)> is the average intensity

[e] $R = \Sigma_{hkl} \mid |F_{obs}| - |F_{calc}| \mid / \Sigma_{hkl} |F_{obs}|$, where R_{free} is calculated without a sigma cut off for a randomly chosen 5% of reflections, which were not used for structure refinement, and R_{work} is calculated for the remaining reflections

[f] Deviations from ideal bond lengths/angles

[g] Percentage of residues in favored / allowed / outlier region

Materials and Methods

Kinetic endpoint measurements with the yeast 20S proteasome

Assays were conducted with 50 µM of purified EpxF or its mutants. The enzyme was mixed with the assay buffer (20 mM Tris-HCl, pH 7.5, 200 mM NaCl) and 400 µM of the substrate **7** were added to start the reaction. 1 mL assays were incubated for three hours at 30 °C and 500 rpm and extracted with an equal volume of ethyl acetate. Volatiles were removed, and the extract was diluted in 20 µL DMSO. The relative chymotrypsin-like activity of the yeast 20S proteasome was determined with serial dilutions of the extracts in the presence of Suc-Leu-Leu-Val-Tyr-AMC in a fluorescence-based ($\lambda_{excitation}$ = 360 nm, $\lambda_{emission}$ = 460 nm) as previously described.[45]

NMR-based activity assay

Assays were conducted in 150 µL with 50 µM of purified EpxF or its mutants in assay buffer (20 mM Tris-HCl, pH 7.5, 200 mM NaCl, 10 % D_2O). 400 µM of the substrate (100 mM stock solution of a mixture of [$^{13}C_2$]**7** and *epi*-[$^{13}C_2$]**7** in DMSO) were added to start the reaction, run for 3 h at 30 °C and 500 rpm and transferred to a NMR microtube. DEPT-135 carbon NMR spectra were recorded on an AVHD-500cryo by the company Bruker at 300 K (10k scans). The chemical shifts are given in δ-values (ppm). 1 mg of epoxomicin (BLD Pharm) was used in reference measurements in the assay buffer. See pages 64 – 75 for NMR spectra.

Computation of docking structures

Structural models of EpxF (crystal data) and substrate **7** (created using ACEDRG[46]) were prepared for docking calculations by adding non-polar hydrogens and Kollman United Atom charges using AutoDockTools 1.5.7 (ADT). Grid boxes for the docking simulations were defined using ADT, the origin of a cube was manually set to the active site of an EpxF-Monomer with a total size of 27000 A^3. Parameters were maintained at the default configuration. Subsequently, the docking was performed with the AutoDock Vina tool based on a fixed geometry of the protein and FAD. PyMol was used to analyze nine output binding modes with a calculated binding affinity of -6.6 to -6.9 kcal/mol of **7** to EpxF and revealed only one biochemically meaningful binding mode to the active site.[39,40]

Cloning and Protein Expression

EpxF from *Goodfellowia coeruleoviolacea* ATCC 53904 was cloned into a pETDuet expression vector modified to encode an *N*-terminal His6-SUMO tag using the restriction enzymes *Bam*HI and *Pst*I. Mutagenesis was conducted following the instructions of the Q5 Site-Directed Mutagenesis Kit (NEB) and correct insertion was verified by Sanger sequencing (Eurofins genomics). The used oligonucleotides are presented in Table S1. *Escherichia coli* strains were grown in lysogeny broth (LB) medium supplemented with 50 mg/L kanamycin. DNA isolation and manipulations were carried out according to standard methods. *E. coli* BL21(DE3) cells were transformed by electroporation and grown in glass shake flasks containing 3 L lysogenic broth with 50 mg/L kanamycin at 37 °C. After reaching an optical density measured at a wavelength of 600 nm (OD_{600}) of 0.6-0.8, flasks were stored at 4 °C for 30 minutes before adding isopropyl-β-*D*-1-thiogalactopyranoside to a final concentration of 0.5 mM to induce protein expression. After incubation with shaking overnight at 20 °C, cells were harvested by centrifugation, washed with 0.9% (w/v) sodium chloride, and cell pellets were stored at −20 °C until further use.

Protein purification protocol

E. coli pellets were dissolved in buffer A (100 mM Tris/ HCl pH 7.5, 100 mM NaCl, 20 mM imidazole, 2 mM 2-mercaptoethanol) and lysed by sonication (Branson Digital Sonifier 250). After centrifugation (40,000g, 4 °C, 30 min), the supernatant was applied onto a 5 ml HisTrap HP column at a flow rate of 5 mL/min with an ÄKTA Pure system (Cytiva), previously equilibrated with buffer A. After washing with buffer A, EpxF was eluted by applying a linear gradient from 0 - 100 % buffer B (100 mM Tris/ HCl pH 7.5, 100 mM NaCl, 500 mM imidazole, 2 mM 2-mercaptoethanol) in 10 column volumes. Fractions containing protein were pooled and supplemented with SUMO-protease to remove the His6-SUMO-tag and dialyzed overnight at 4 °C against buffer C (20 mM Tris/ HCl pH 7.5, 100 mM NaCl, 2 mM 2-mercaptoethanol). HisTrap affinity chromatography was repeated and the flow-through was concentrated to 4 ml using Amicon Ultra-15 centrifugal filters (30,000 MWCO). Centrifugation (16,000g, 4 °C, 15 min) removed residual protein aggregates, and the supernatant was used for size exclusion chromatography with a HiLoad Superdex 200 pg 16/600 column in buffer D (20 mM Tris/ HCl pH 7.5, 100 mM NaCl, 2 mM dithiothreitol) at 1.0 mL/min. Purity was assigned by Coomassie-stained SDS-PAGE analysis, pure fractions were pooled and concentrated to 50 mg/mL.

Crystallization and structure determination of EpxF

50 mg/mL of EpxF were mixed with 2 mM of FAD and incubated at 4 °C for 1 hour. Precipitant was removed by centrifugation (16,000g, 4 °C, 15 min) and the solution was subsequently used for crystallization trials. Sparse-matrix screens were set up with a drop ratio of 0.2 µL + 0.2 µL, 0.2 µL + 0.1 µL, or 0.3 µL + 0.1 µL at 20°C. Crystals grew within three weeks in 0.3 µL EpxF + 0.1 µL reservoir conditions containing 100 mM NaCl, 1.8 M AmSO4, 0.1 M HEPES pH 7.6, 2.0 M NDSB-221, and were cryo-protected with 30 % ethylene glycol before vitrification in liquid nitrogen. Data sets were recorded at the beam line X06SA at the Swiss Light Source in Villingen, Switzerland.

The XDS software package was used for initial data processing and scaling.[47] Details of data collection and analysis are listed in Table S2. All further steps for structure solution were performed using programs of the CCP4 software package.[48] Conventional crystallographic rigid body, positional, and temperature factor refinements were carried out with REFMAC5[49] using coordinates of 4N5F (https://doi.org/10.2210/pdb4N5F/pdb) as starting model. For model building, the program COOT[50] was used. The final coordinates yielded excellent R factors, as well as geometric bond and angle values. Coordinates were confirmed to fulfill the Ramachandran plot and have been deposited in the RCSB.

General Remarks on Chemical Synthesis

Room temperature is defined as 21–23 °C. All reactions with air sensitive reactants were carried out under an argon atmosphere (Ar 4.8) applying standard *Schlenk* technique.

Solvents and Reagents

Unless otherwise noted, all chemicals were obtained from Sigma-Aldrich, Acros, TCI, abcr, Fisher Scientific, BLD Pharm, or Alfa Aesar and used without further purification. Dichloromethane (2×MB-KOLA type 2, aluminium oxide), diethyl ether (1×MB-KOL-A type 2, aluminium oxide), and tetrahydrofuran (2×MB-KOL-M type 2, 3 Å molecular sieves) were obtained from an MBSPS 800 MBraun solvent purification system.

Irradiation Setup

Photochemical reactions were carried out in crimp-cap vials using 1W 451 nm LEDs (Osram Oslon SSL 80 LDCQ7P-2U3U, 1W, 451 nm, 80°) (Fig. S4).

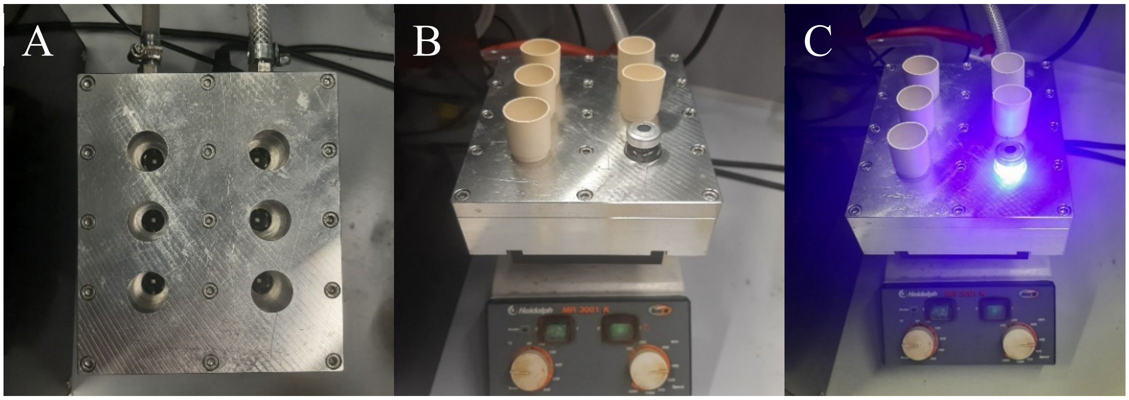

Figure S4. Irradiation setup (λ_{max} = 451 nm) with six slots from above (left), turned off (middle), and turned on (right).

Thin Layer Chromatography (TLC) and Column Chromatography
Thin layer chromatography was carried out with TLC glass plates purchased from Merck (0.25 mm, silica 60, F_{254}). Spots were detected by UV light (λ = 254 nm), color, and standard stains, i.e. $KMnO_4$ stain, I_2 stain (developing by iodine vapour), ninhydrine stain. Preparative flash column chromatography was performed on silica 60 (Merck, 230-400 mesh).

Nuclear Magnetic Resonance (NMR) Spectroscopy
NMR spectra were recorded on Bruker AVHD500-, AV400-, AV300- and AV500cryo-devices at 298 K. Chemical shifts are given in the delta-scale (ppm) and ^{1}H-NMR-spectra are referenced to the residual proton signals of the deuterated solvents ($CDCl_3$ = 7.26 ppm, MeOH–d_4 = 3.31 ppm, DMSO–d_6 = 2.50 ppm, CD_2Cl_2 = 5.32 ppm). ^{13}C-NMR-spectra are referenced the deuterium-coupled ^{13}C-signals of the solvents ($CDCl_3$ = 77.2 ppm, MeOH–d_4 = 49.0 ppm, DMSO–d_6 = 39.5 ppm). Solvents were supplied by Sigma Aldrich or Deutero. The following abbreviations were used for the assignments of the multiplets: s-singlet, d-doublet, t-triplet. q-quartet, quint-quintet, sex-sextet, hept-heptet, m-multiplet, br-broad, *virt.*-virtual. Coupling constants *J* [Hz] were calculated as the average of the coupling and back-coupling. For signal assignments the following experiments were used. ^{1}H-^{13}C-HSQC-ME, ^{1}H-^{13}C-HMBC, ^{1}H-^{1}H-COSY and ^{1}H-^{1}H-NOESY. In cases where an unambiguous assignment was not possible, it is indicated by "/", while "," is used in cases where resonances of two or more atoms overlap. Apparent multiplets which occur as a result of accidental equality of coupling constants to those of magnetically non-equivalent protons are marked as virtual (*virt.*).

High Resolution Mass Spectrometry (HR-MS)
High-resolution mass spectrometry was performed on a Thermo Scientific Q Exactive Plus (HRESI) equipped with an orbitrap mass analyzer.

Ultra Performance Liquid Chromatography Mass Spectrometry (UPLC-MS)
Ultra Performance Liquid Chromatography Mass Spectrometry was performed on a Dionex UltiMate 3000 UHPLC device with a Rs variable wavelength detector (200 nm) and a C18 reversed phase column coupled to a Thermo Scientific LCQ Fleet mass spectrometer (electrospray ionization, positive mode). A water/acetonitrile (containing 0.1 v/v% formic acid) gradient (90/10→10/90 over 8 min) and a flow rate of 0.9 mL/min was applied.

Infrared (IR) Spectroscopy

Infrared spectra were recorded on a Perkin Elmer Frontier ATR/FT-IR spectrometer, and $\tilde{\nu}_{max}$ are reported in cm^{-1}. The signal intensity is assigned using the following abbreviations: vs-very strong, s-strong, m-medium, w-weak, br-broad.

Synthetic Procedures

Methyl (*S*)-4-((tert-butoxycarbonyl)amino)-6-methyl-3-oxoheptanoate (Compound **S1**)

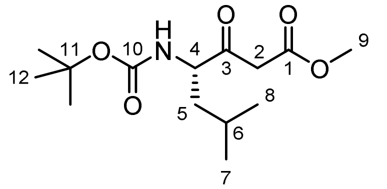

Compound **S1** is synthesized according to a literature procedure.[51] To a solution of Boc-L-Leu-OH (hydrate, 500 mg, 2.16 mmol, 1.00 equiv.) in THF (anhydrous, 10.8 mL, 200 mM) in a flame-dried round bottom flask is added carbonyldiimidazole (701 mg, 4.32 mmol, 2.00 equiv.) under an atmosphere of argon. The reaction mixture is stirred at RT for 2 h. Then, $MgCl_2$ (206 mg, 2.16 mmol, 1.00 equiv.) and potassium methylmalonate (506 mg, 3.24 mmol, 1.50 equiv.) are added. After stirring at RT for 24 h, the reaction is diluted with ethyl acetate (50 mL), washed with citric acid (aqueous, 10% (*w/v*), 1 x 20 mL), $NaHCO_3$-solution (aqueous, saturated, 1 x 20 mL) and brine (1 x 20 mL). The combined organic layers are dried over Na_2SO_4, filtered, and all volatiles are removed *in vacuo*. Purification by column chromatography (silica, *n*-pentane/EtOAc = 90/10 → 80/20) yields **S1**.

Colorless oil, 482 mg (1.68 mmol, 78%), **TLC**: $R_f = 0.61$ (*n*-pentane/EtOAc = 70/30); **^{1}H NMR** (500 MHz, $CDCl_3$, 298 K) δ (ppm) = 4.90 (d, $^3J_{H\text{-}H} = 8.0$ Hz, 1H, N*H*), 4.34 (m, 1H, H^4), 3.74 (s, 3H, H^9), 3.60 (d, $^2J_{H\text{-}H} = 15.9$ Hz, 1H, H^{2a}), 3.54 (d, $^2J_{H\text{-}H} = 15.9$ Hz, 1H, H^{2b}), 1.71 (m, 1H, H^6), 1.61 (m, 1H, H^{5a}), 1.44 (m, 10H, $H^{5b,12}$), 0.95 (d, $^3J_{H\text{-}H} = 6.6$ Hz, 3H, $H^{7/8}$), 0.94 (d, $^3J_{H\text{-}H} = 6.6$ Hz, 3H, $H^{7/8}$).

The analytical data is in accordance with literature.[51]

Methyl 4-((*tert*-butoxycarbonyl)amino)-2,2,6-trimethyl-3-oxoheptanoate (Compound **15**)

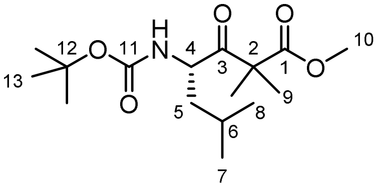

Compound **15** is synthesized according to a literature procedure.[52] K_2CO_3 (1.07 g, 7.71 mmol, 4.00 equiv.) and methyl iodide (1.37 g, 600 µL, 9.64 mmol, 5.00 equiv.) are added to a solution of **S1** (554 mg, 1.93 mmol, 1.00 equiv.) in DMF (6.43 mL, 300 mM). After stirring at RT overnight, all volatiles are removed *in vacuo* using a cooling trap. The crude material is purified by column chromatography (silica, *n*-pentane/EtOAc = 95/5).

Colorless oil, 546 mg (1.73 mmol, 90%), TLC: R_f = 0.29 (*n*-pentane/EtOAc = 95/5); ^{1}H NMR (500 MHz, $CDCl_3$, 298 K) δ (ppm) = 4.78 (d, $^3J_{H\text{-}H}$ = 9.9 Hz, 1H, N*H*), 4.64 (ddd, $^3J_{H\text{-}H}$ = 10.2 Hz, 9.9 Hz, 3.4 Hz, 1H, H^4), 3.73 (s, 3H, H^{10}), 1.68 (m, 1H, H^6), 1.42 (m, 17H, $H^{5,9,13}$), 0.95 (d, $^3J_{H\text{-}H}$ = 6.6 Hz, 3H, $H^{7/8}$), 0.92 (d, $^3J_{H\text{-}H}$ = 6.6 Hz, 3H, $H^{7/8}$).

The analytical data is in accordance with literature.[52]

(*S*)-4-((*tert*-Butoxycarbonyl)amino)-2,2,6-trimethyl-3-oxoheptanoic acid (Compound **10**)

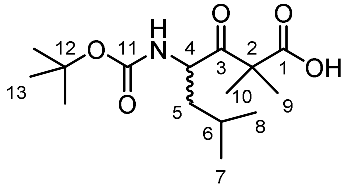

Compound **10** was synthesized by a modified literature procedure.[52] A solution of LiOH (5.43 mg, 227 µmol, 1.50 equiv.) in H_2O (deionized, 410 µL) is added to a solution of compound **15** (47.7 mg, 151 µmol, 1.00 equiv.) in THF (anhydrous, 410 µL) at T = 0 °C. After stirring at this temperature for 20 min, the reaction is poured on ice and extracted with CH_2Cl_2 (3 x 5 mL). The aqueous phase is cooled to 0 °C, carefully acidified to pH = 3 with 1 N sulfuric acid, and extracted with CH_2Cl_2 (4 x 5 mL). The organic layer is dried over Na_2SO_4, filtered, and all volatiles are removed *in vacuo* at T = 0 °C to avoid decarboxylation.

Colorless solid (44.4 mg, 147 µmol, 97%)*; **^{1}H NMR** (500 MHz, $CDCl_3$, 298 K) δ (ppm) = *Isomer A*: 5.00 (d, $^3J_{H\text{-}H}$ = 9.4 Hz, 1H, N*H*), 4.69 (ddd, $^3J_{H\text{-}H}$ = 10.8, 9.4, 3.1 Hz, 1H, H^4), 1.78 – 1.63 (m, 1H, H^6), 1.60 – 1.23 (m, 17H, $H^{5,9,10,13}$), 0.97 – 0.87 (m, 6H, $H^{7,8}$); *Isomer B:* 6.46 (s, 1H, N*H*), 4.34 (*virt.* t, $^3J_{H\text{-}H} \approx {}^3J_{H\text{-}H'}$ = 9.80 Hz, 1H, H^4), 1.78 – 1.63 (m, 1H, H^6), 1.60 – 1.23 (m, 17H, $H^{5,9,10,13}$), 0.97 – 0.87 (m, 6H, $H^{7,8}$); **^{13}C NMR** (126 MHz, $CDCl_3$, 298 K) δ (ppm) = *Isomer A:* 209.4 (s, C3), 177.2 (s, C1), 155.5 (s, C11), 80.4 (s, C12), 54.6 (s, C2), 54.4 (s, C4), 41.7 (s, C5), 28.4 (s, 3C, C^{13}), 24.9 (s, C^6), 23.7 (s, $C^{7/8/9/10}$), 22.6 (s, $C^{7/8/9/10}$), 22.2 (s, $C^{7/8/9/10}$), 21.4 (s, $C^{7/8/9/10}$); *Isomer B:* 208.6 (s, C^3), 177.8 (s, C^1), 157.0 (s, C^{11}), 82.2 (s, C^{12}), 57.9 (s, C^4), 53.5 (s, C^2), 41.1 (s, C^5), 28.3 (s, 3C, C^{13}), 24.4 (s, $C^{6/7/8/9/10}$), 23.9 (s, $C^{6/7/8/9/10}$), 23.5 (s, $C^{6/7/8/9/10}$), 21.8 (s, $C^{6/7/8/9/10}$), 21.2 (s, $C^{6/7/8/9/10}$). **HR-ESI-MS** (ESI^+): *m/z* = calc. for $[C_{15}H_{27}NNaO_5]^+$: 324.1781 ($[M+Na]^+$), found: 324.1776; *m/z* = calc. for $[C_{15}H_{27}KNO_5]^+$: 340.1526 ($[M+K]^+$), found: 340.1516; *m/z* = calc. for $[C_{30}H_{54}NaNO_{10}]^+$: 625.3671 ($[2M+Na]^+$), found: 625.3663; **IR:** (ATR) $\tilde{\nu}$ = 3320 (w), 3270 (w), 3113 (w), 2982 (m), 2960 (m), 2933 (m), 2871 (w), 2634 (w), 2544 (w), 1713 (s), 1655 (s), 1472 (w), 1399 (m), 1368 (m), 1282 (m), 1243 (w), 1214 (w), 1182 (m), 1162 (m), 1148 (m), 1112 (m), 1033 (w), 1011 (m), 997 (m), 960 (w), 932 (w), 918 (m), 871 (m), 845 (w), 828 (w), 796 (w), 775 (m), 762 (w), 734 (w), 675 (w). *Contains 2% (*w/w*) of THF.

The acidic proton was not observed in $CDCl_3$. No decarboxylation product (ketone, *vide infra*) was detected by NMR spectroscopy.

Compound **10** forms two isomers in $CDCl_3$ solution. This phenomenon is clearly observed by 2D NMR: The ^{1}H,^{1}H-COSY experiment shows two sets of N*H* and C*H* positions (Fig. S5). The ^{1}H,^{1}H-NOESY spectrum confirms that both species exchange on the NMR timescale, as the sign of the phase of these cross-peaks and the diagonal cross-peaks is identical (Fig. S6).

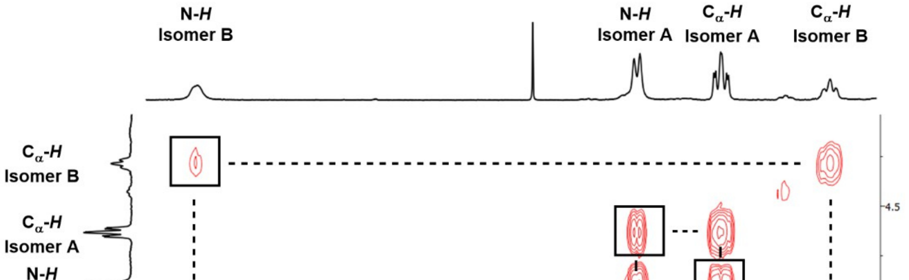

Figure S5. The ^{1}H,^{1}H-COSY NMR spectrum of compound **10** in $CDCl_3$.

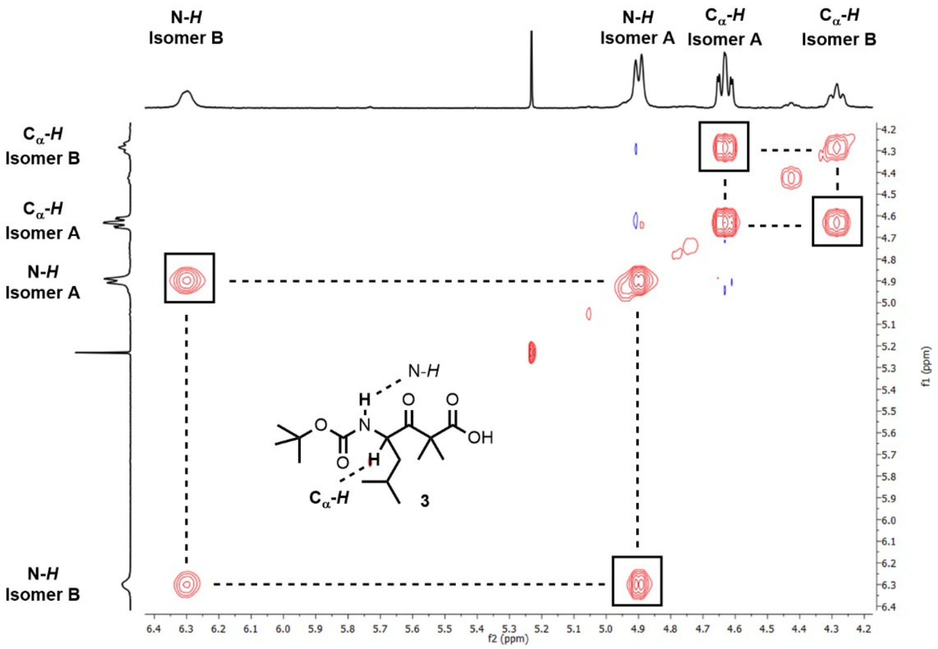

Figure S6. The ^{1}H,^{1}H-NOESY NMR spectrum of compound **10** in $CDCl_3$.

tert-Butyl (*S*)-(2,6-dimethyl-3-oxoheptan-4-yl)carbamate (Compound **11**)

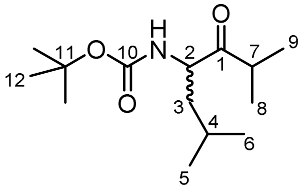

Compound **11** is prepared by saponification and decarboxylation of compound **15**. A solution of KOH (394 mg, 7.03 mmol, 4.00 equiv.) dissolved in H_2O (2 mL) is added to a solution of **15** (554 mg, 1.76 mmol, 1.00 equiv.) in MeOH (anhydrous, 2.93 mL, 600 mM). The reaction is stirred at RT for 30 min, after which completion of the reaction was confirmed *via* TLC. The reaction mixture is diluted with H_2O (10 mL) and EtOAc (20 mL) and the aqueous phase is extracted with EtOAc (3 x 10 mL). The combined organic layers are washed with brine, dried over Na_2SO_4, filtered and all volatiles are removed *in vacuo*. No further purification was necessary.

Colorless solid (436 mg, 1.69 mmol, 96%), **TLC:** $R_f = 0.45$ (*n*-pentane/EtOAc = 95/5); **^{1}H NMR** (500 MHz, $CDCl_3$, 298 K) δ (ppm) = 5.00 (d, $^3J_{H\text{-}H} = 8.6$ Hz, 1H, N*H*), 4.49 (ddd, $^3J_{H\text{-}H} = 9.3, 9.0, 3.7$ Hz, 1H, H^2), 2.80 (*virt.* hept., $^3J_{H\text{-}H} \approx {}^3J_{H\text{-}H} = 6.9$ Hz, 1H, H^7), 1.77 – 1.66 (m, 1H, H^4), 1.53 – 1.44 (m, 1H, H^{3a}), 1.47 (s, 9H, H^{12}), 1.30 (ddd, $^2J_{H\text{-}H} = 14.2$ Hz, $^3J_{H\text{-}H} = 10.0, 4.5$ Hz, 1H, H^{3b}), 1.14 (d, $^3J_{H\text{-}H} = 7.0$ Hz, 3H, $H^{8/9}$), 1.10 (d, $^3J_{H\text{-}H} = 6.7$ Hz, 3H, $H^{8/9}$), 0.98 (d, $^3J_{H\text{-}H} = 6.5$ Hz, 3H, $H^{5/6}$), 0.92 (d, $^3J_{H\text{-}H} = 6.7$ Hz, 3H, $H^{5/6}$); **^{13}C NMR** (126 MHz, $CDCl_3$, 298 K) δ (ppm) = 214.2 (C^1), 155.6 (C^{10}), 79.7 (C^{11}), 56.2 (C^2), 41.2 (C^3), 38.0 (C^7), 28.5 (3C, C^{12}), 25.1 (C^4), 23.6 ($C^{5/6}$), 21.9 ($C^{5/6}$), 19.2 ($C^{8/9}$), 17.9 ($C^{8/9}$).

The analytical data is in accordance with literature.[21]

<u>*tert*-Butyl (*S*)-(1-(methoxy(methyl)amino)-4-methyl-1-oxopentan-2-yl)carbamate (Compound **S2**)</u>

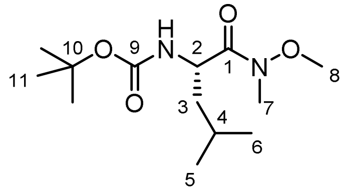

Compound **S2** is prepared according to a literature procedure.[29] *N*-Boc-L-Leu-OH (1.00 g, 4.32 mmol, 1.00 equiv.) is dissolved in CH_2Cl_2 (anhydrous, 18 mL, 240 mM) and cooled to T = 0 °C. HOBt (728 mg, 4.76 mmol, 1.10 equiv.) and EDCI (995 mg, 5.19 mmol, 1.20 equiv) are added and the reaction mixture is stirred for 15 min at T = 0 °C, after which *N*,*O*-dimethyl hydroxylamine hydrochloride (485 mg, 4.97 mmol, 1.15 equiv.) and *N*-methylmorpholine (525 mg, 577 µL, 5.19 mmol, 1.20 equiv.) are added. The reaction is allowed to warm up to RT and stirred overnight. Purification by column chromatography (*n*-pentane/EtOAc = 80/20 →70/30) yields **S2**.

Colorless oil (1.13g, 4.13 mmol, 96%), TLC: R_f = 0.05 (*n*-pentane/EtOAc), ^{1}H NMR (500 MHz, $CDCl_3$, 298 K) δ (ppm) = 5.04 (d, $^3J_{H\text{-}H}$ = 9.6 Hz, 1H, N*H*), 4.76 – 4.67 (m, 1H, H^2), 3.78 (s, 3H, H^8), 3.20 (s, 3H, H^7), 1.78 – 1.66 (m, 1H, H^4), 1.50 – 1.38 (m, 11H, $H^{3,11}$), 0.96 (d, $^3J_{H\text{-}H}$ = 6.6 Hz, 3H, $H^{5/6}$), 0.93 (d, $^3J_{H\text{-}H}$ = 6.7 Hz, 3H, $H^{5/6}$).

The analytical data is in accordance with literature.[29]

tert-Butyl (S)-(2,6-dimethyl-3-oxohept-1-en-4-yl)carbamate (Compound **14**)

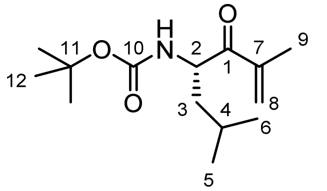

α,β-unsaturated ketone **14** was prepared according to a literature procedure.[29] Weinreb amide **S2** (640 mg, 2.33 mmol, 1.00 equiv.) is dissolved in THF (anhydrous,4.67 mL, 500 mM) and cooled to -78 °C. Isopropenylmagnesium bromide (0.5 M in THF, 1.02 g, 14 mL, 3.00 equiv.) is added dropwise while stirring and the reaction is allowed to slowly warm up to RT and stirred overnight. The reaction is quenched by the addition of NH_4Cl solution (aqueous, saturated 10 mL) and the aqueous layer is extracted with CH_2Cl_2 (3 x 15 mL). The combined organic layers are dried over Na_2SO_4, filtered and all solvents are removed *in vacuo*. Purification by column chromatography (*n*-pentane/EtOAc = 95/5) yields **14**.

Colorless solid (514 mg, 2.01 mmol, 86%), **TLC:** R_f = 0.38 (*n*-pentane/EtOAc), **^{1}H NMR** (500 MHz, $CDCl_3$, 298 K) δ (ppm) = 6.08 (s, 1H, H^{8a}), 5.91 – 5.86 (m, 1H, H^{8b}), 5.14 (d, $^3J_{H\text{-}H}$ = 9.0 Hz, 1H, N*H*), 5.06 (ddd, $^3J_{H\text{-}H}$ = 9.5, 9.2, 3.6 Hz, 1H, H^2), 1.90 (s, 3H, H^9), 1.78 – 1.68 (m, 1H, H^4), 1.50 – 1.40 (m, 10H, $H^{3a,12}$), 1.32 (ddd, $^2J_{H\text{-}H}$ = 14.1 Hz, $^3J_{H\text{-}H}$ = 10.0, 4.4 Hz, 1H, H^{3b}), 1.00 (d, $^3J_{H\text{-}H}$ = 6.6 Hz, 3H, $H^{5/6}$), 0.90 (d, $^3J_{H\text{-}H}$ = 6.7 Hz, 1H, $H^{5/6}$); **^{13}C NMR** (126 MHz, $CDCl_3$, 298 K) δ (ppm) = 201.8 (C^1), 155.7 (C^{10}), 142.4 (C^7), 126.3 (C^8), 79.7 (C^{11}), 52.7 (C^2), 43.4 (C^3), 28.5 (3C, C^{12}), 25.1 (C^4), 23.5 ($C^{5/6}$), 21.9 ($C^{5/6}$), 18.0 (C^9).

The analytical data is in accordance with literature.[29]

tert-Butyl ((2S)-4-methyl-1-(2-methyloxiran-2-yl)-1-oxopentan-2-yl)carbamate (Compound **16**)

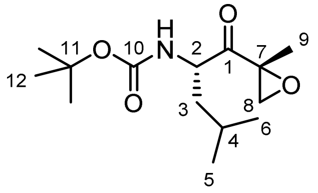

Epoxyketone **16** was prepared according to a literature procedure.[29] The α,β-unsaturated ketone **14** (77.5 mg, 304 µmol, 1.00 equiv.) is dissolved in MeOH (anhydrous, 1.01 mL, 300 mM) and cooled to 0 °C. Benzonitrile (313 µL, 3.03 mmol, 10.0 equiv.), H_2O_2 (aqueous, 30% (*w/v*), 860 µL, 7.59 mmol, 25 equiv.) and *N*,*N*-diisopropylethylamine (529 µL, 3.03 mmol, 10.0 equiv.) are added dropwise successively. After stirring for 4 h at 0 °C the reaction is terminated by the addition of H_2O (10 mL). The aqueous layer is extracted with CH_2Cl_2 (3 x 15 mL), and the combined organic layers are dried over Na_2SO_4 and filtered. After removal of all volatiles *in vacuo*, purification by column chromatography (*n*-pentane/EtOAc = 95/5, silica was deactivated with triethylamine (1% (*v/v*) in the eluent) before use) yields **16**.

Light yellow oil (7 mg, 25.8 µmol, 9%), **TLC:** R_f = 0.25 (*n*-pentane/EtOAc), **^{1}H NMR** (500 MHz, $CDCl_3$, 298 K) δ (ppm) = 4.84 (d, $^3J_{H\text{-}H}$ = 8.9 Hz, 1H, N*H*), 4.31 (ddd, $^3J_{H\text{-}H}$ = 10.4, 8.8, 3.1 Hz, 1H, H^2), 3.29 (d, $^2J_{H\text{-}H}$ = 5.0 Hz, 1H, H^{8a}), 2.89 (d, $^2J_{H\text{-}H}$ = 5.0 Hz, 1H, H^{8b}), 1.77 – 1.68 (m, 1H, H^4), 1.51 (s, 3H, H^9), 1.50 – 1.46 (m, 1H, H^{3a}), 1.40 (s, 9H, H^{12}), 1.17 (ddd, $^2J_{H\text{-}H}$ = 14.1 Hz, $^3J_{H\text{-}H}$ = 10.4, 4.1 Hz, 1H, H^{3b}), 0.96 (d, $^3J_{H\text{-}H}$ = 6.5 Hz, 3H, $H^{5/6}$), 0.93 (d, $^3J_{H\text{-}H}$ = 6.7 Hz, 3H, $H^{5/6}$).; **^{13}C NMR** (126 MHz, $CDCl_3$, 298 K) δ (ppm) = 209.8 (C^1), 155.8 (C^{10}), 79.9 (C^{11}), 59.2 (C^7), 52.5 (C^8), 51.5 (C^2), 40.5 (C^3), 28.4 (3C, C^{12}), 25.2 (C^4), 23.6 ($C^{5/6}$), 21.4 ($C^{5/6}$), 16.9 (C^9).

Methyl 4-((*tert*-butoxycarbonyl)amino)-6-methyl-2,2-di(methyl-^{13}C)-3-oxoheptanoate (Compound [$^{13}C_2$]**15**)

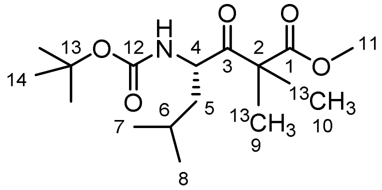

To a solution of **S1** (209 mg, 727 µmol, 1.00 equiv.) in DMF (anhydrous, 2.42 ml; 300 mM) is added K_2CO_3 (402 mg, 2.91 mmol, 4.00 equiv.) and [^{13}C]MeI (312 mg, 136 µL, 2.18 mmol, 3.00 equiv.). After stirring at RT for 7 h the reaction is diluted with CH_2Cl_2, washed with $NaHCO_3$-solution (aqueous, saturated, 10 mL), and the aqueous layer is extracted with CH_2Cl_2 (3 x 10 mL). The combined organic layers are dried over Na_2SO_4, filtered and all volatiles are removed *in vacuo*. Purification by column chromatography (silica, *n*-pentane/EtOAc = 95/5) yields [$^{13}C_2$]**15**.

Colorless solid (191 mg, 601 µmol, 83%), **TLC:** R_f = 0.82 (*n*-pentane/EtOAc = 80/20) [UV]; **^{1}H NMR:** (500 MHz, $CDCl_3$, 298 K) δ (ppm) = 4.78 (d, $^3J_{H\text{-}H}$ = 9.8 Hz, 1H, N*H*), 4.64 (ddd, $^3J_{H\text{-}H}$ = 10.2, 10.1, 3.4 Hz, 1H, H^4), 3.72 (s, 3H, H^{11}), 1.73 – 1.64 (m, 1H, H^6), 1.55 (dd, $^1J_{C\text{-}H}$ = 130.4 Hz, $^3J_{C\text{-}H}$ = 4.6 Hz, 3H, $H^{9/10}$), 1.54 (dd, $^1J_{C\text{-}H}$ = 130.4 Hz, $^3J_{C\text{-}H}$ = 4.7 Hz, 3H, $H^{9/10}$), 1.42 (s, 9H, H14), 1.41 – 1.31 (m, 2H, H^5), 0.95 (d, $^3J_{H\text{-}H}$ = 6.5 Hz, 3H, $H^{7/8}$), 0.92 (d, $^3J_{H\text{-}H}$ = 6.7 Hz, 3H, $H^{7/8}$); **^{13}C{1H} NMR:** (126 MHz, $CDCl_3$, 298 K) δ (ppm) = 209.0 (C^3), 173.7 (C^1), 155.2 (C^{12}), 79.9 (C^{13}), 54.87 (t, $^1J_{C\text{-}C}$ = 34.5 Hz, C^2), 53.9 (C^4), 52.8 (C^{11}), 42.0 (C^5), 28.4 (3C, C^{14}), 24.8 (C^6), 23.7 ($C^{7/8}$), 22.4 ($C^{9/10}$), 22.2 ($C^{9/10}$), 21.5 ($C^{7/8}$); **HR-ESI-MS** (ESI^+): *m/z* = calc. for $[C_{14}{}^{13}C_2H_{29}NNaO_5]^+$: 340.2005 ($[M+Na]^+$), found: 340.2004; *m/z* = calc. for $[C_{14}{}^{13}C_2H_{29}KNO_5]^+$: 356.1744 ($[M+K]^+$), found: 356.1743; **IR:** (ATR) $\tilde{\nu}$ = 3383 (m, N-H), 2957 (m, CH_2), 2937 (m, CH_2), 2871 (w, CH_2), 1702 (s, C=O), 1505 (s), 1471 (m), 1456 (m), 1439 (m), 1393 (w), 1381 (w), 1365 (m), 1354 (w), 1333 (w), 1287 (m), 1268 (m), 1249 (m), 1198 (w), 1155 (s), 1146 (s), 1083 (m), 1046 (m), 1023 (m), 1012 (w), 1001 (w), 985 (w), 945 (w), 931 (w), 894 (w), 876 (w), 845 (m), 784 (w), 772 (w), 745 (w).

Benzyl (*tert*-butoxycarbonyl)-L-isoleucinate (Compound **S3**)

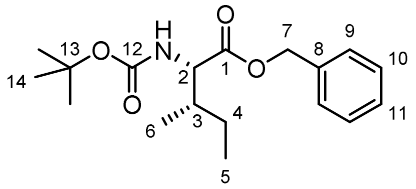

Compound **S3** was prepared according to a literature procedure.[53] *N*-Boc-L-Ile-OH (1.00 g, 4.32 mmol, 1.00 equiv.) is dissolved in MeCN (anhydrous, 28.8 mL, 150 mM). K_2CO_3 (1.20 g, 8.65 mmol, 2.00 equiv.) and benzylbromide (1.11 g, 770 µL, 6.49 mmol, 1.50 equiv.) are added and the reaction is stirred at RT overnight. The reaction mixture is partitioned between EtOAc (100 mL) and brine (50 mL) and the aqueous phase was extracted with EtOAc (3 x 20 mL). The organic layer is dried over Na_2SO_4, filtered and all volatiles are removed *in vacuo*. Purification by column chromatography (silica, *n*-pentane/EtOAc = 95/5 → 80/20) yielded **S3**.

Colorless oil (1.38g, 4.29 mmol, 99%), **TLC:** $R_f = 0.63$ (*n*-pentane/EtOAc = 90/10) [UV or Ninhydrine]. **^{1}H NMR:** (500 MHz, $CDCl_3$, 298 K) δ (ppm) = 7.42 – 7.28 (m, 5H, $H^{9,10,11}$), 5.22 (d, $^2J_{H\text{-}H}$ = 12.3 Hz, 1H, H^{7a}), 5.11 (d, $^2J_{H\text{-}H}$ = 12.2 Hz, 1H, H^{7b}), 4.09 (d, $^3J_{H\text{-}H}$ = 6.0 Hz, 1H, H^2), 1.87 – 1.77 (m, 1H, H^3), 1.47 – 1.34 (m, 10H, $H^{4a,14}$), 1.24 – 1.12 (m, 1H, H^{4b}), 0.90 – 0.84 (m, 6H, $H^{5,6}$).

The analytical data is in accordance with literature.[54]

Benzyl *N*-(((9*H*-fluoren-9-yl)methoxy)carbonyl)-*N*-methyl-L-isoleucyl-L-isoleucinate (Compound **S4**)

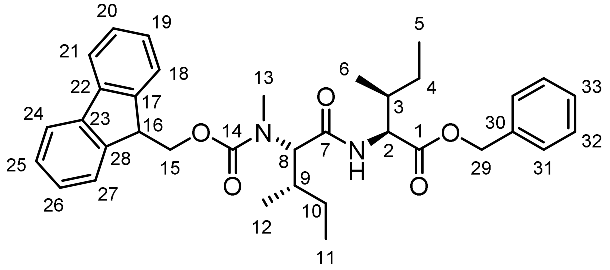

Compound **S3** (703 mg, 2.19 mmol, 1.00 equiv.) is deprotected using HCl (4 M in dioxane, 2.73 mL, 5.00 equiv.). All volatiles are removed *in vacuo* after completion of the reaction is confirmed by TLC and the residue is dissolved in CH_2Cl_2 (anhydrous, 21.9 mL, 100 mM) and cooled to T = 0 °C. Fmoc-*N*(Me)-L-Ile-OH (804 mg, 2.19 mmol, 1.00 equiv.) is added to the reaction mixture followed by HOBt (352 mg, 2.30 mmol, 1.05 equiv.), HBTU (871 mg, 2.30 mmol, 1.05 equiv.) and *N*,*N*-diisopropylethylamine (594 mg, 800 µL, 4.59 mmol, 2.10 equiv.). The reaction mixture is warmed up to RT overnight under constant stirring and then diluted with citric acid (aqueous, 10% (*w/v*), 20 mL). The aqueous layer is extracted with CH_2Cl_2 (3 x 10 mL) and the combined organic layers are washed with $NaHCO_3$-solution (aqueous, saturated, 20 mL) and brine (20 mL). After drying over Na_2SO_4 and filtration, all volatiles are removed *in vacuo*. Purification by column chromatography (silica, *n*-pentane/EtOAc = 80/20) yields **S4**.

Colorless oil (1.20 g, 2.10 mmol, 96%); **TLC**: $R_f = 0.17$ (*n*-pentane/EtOAc = 90/10) [UV]. **^{1}H NMR:** (500 MHz, $CDCl_3$, 298 K) δ (ppm) = *Major Rotamer*: 7.80 – 7.71 (m, 2H, $H^{21,24}$), 7.61 – 7.50 (m, 2H, $H^{18,27}$), 7.44 – 7.27 (m, 9H, $H^{19,20,25,26,31,32,33}$), 6.47 (d, $^3J_{H-H}$ = 8.7 Hz, 1H, N*H*), 5.19 (d, $^2J_{H-H}$ = 12.1 Hz, 1H, H^{29a}), 5.10 (d, $^2J_{H-H}$ = 12.3 Hz, 1H, H^{29b}), 4.58 (dd, $^3J_{H-H}$ = 8.8, 4.8 Hz, 1H, H^2), 4.44 (dd, $^2J_{H-H}$ = 10.7 Hz, $^3J_{H-H}$ = 7.0 Hz, 1H, H^{15a}), 4.37 (dd, $^2J_{H-H}$ = 10.6 Hz, $^3J_{H-H}$ = 7.1 Hz, 1H, H^{15b}), 4.25 (t, $^3J_{H-H}$ = 6.6 Hz, 1H, H^{16}), 4.20 (d, $^3J_{H-H}$ = 11.3 Hz, 1H, H^8), 2.88 (s, 3H, H^{13}), 2.15 – 2.06 (m, 1H, H^9), 1.92 – 1.83 (m, 1H, H^3), 1.44 – 1.34 (m, 1H, H^{10a}), 1.32 – 1.23 (m, 1H, H^{10b}), 1.10 – 0.98 (m, 2H, H^4), 0.92 – 0.87 (m, 6H, $H^{12,5/11}$), 0.83 – 0.73 (m, 6H, $H^{6,5/11}$); *Minor Rotamer:* 7.80 – 7.71 (m, 2H, $H^{21,24}$), 7.61 – 7.50 (m, 2H, $H^{18,27}$), 7.44 – 7.27 (m, 9H, $H^{19,20,25,26,31,32,33}$), 6.47 (d, $^3J_{H-H}$ = 8.7 Hz, 1H, N*H*), 5.19 (d, $^2J_{H-H}$ = 12.1 Hz, 1H, H^{29a}), 5.10 (d, $^2J_{H-H}$ = 12.3 Hz, 1H, H^{29b}), 4.87 – 4.78 (m, 1H, H^{15a}), 4.44 (dd, $^2J_{H-H}$ = 10.7 Hz, $^3J_{H-H}$ = 7.0 Hz, 2H, $H^{2,15b}$), 4.25 (t, $^3J_{H-H}$ = 6.6 Hz, 1H, H^{16}), 3.34 (d, $^3J_{H-H}$ = 11.0 Hz, 1H, H^8), 2.75 (s, 3H, H^{13}), 1.83 – 1.77 (m, 1H, H^9), 1.74 – 1.69 (m, 1H, H^3), 1.44 – 1.34 (m, 1H, H^{10a}), 1.32 – 1.23 (m, 1H, H^{10b}), 1.18 – 1.11 (m, 2H, H^4), 0.83 – 0.73 (m, 6H, $H^{5/11,6/12}$), 0.73 – 0.68 (m, 3H, $H^{5/11}$), 0.64 – 0.58 (m, 3H, $H^{6/12}$); **^{13}C{1H} NMR** (126 MHz, $CDCl_3$, 298 K) δ (ppm) = *Major Rotamer*: 171.5 (C^1), 170.1 (C^7), 157.5 (C^{14}), 143.96 ($C^{17/28}$) , 143.94 ($C^{17/28}$), 141.5 ($C^{22/23}$), 141.4 ($C^{22/23}$), 135.5 (C^{30}), 128.7 (2C, $C^{31/32/33}$), 128.53 ($C^{31/32/33}$), 128.50 (2C, $C^{31/32/33}$), 127.9 (2C, $C^{20,25}$), 127.2 (2C, $C^{19,26}$), 125.1 ($C^{18/27}$), 125.0 ($C^{18/27}$), 120.18 ($C^{21/24}$), 120.16 ($C^{21/24}$), 68.0 (C^{15}), 67.1 (C^{29}), 63.9 (C^8), 56.3 (C^2), 47.3 (C^{16}), 37.6 (C^3), 31.8 (C^9), 30.1 (C^{13}), 25.0 (C^4), 24.7 (C^{10}), 15.70 ($C^{6/12}$), 15.67 ($C^{6/12}$), 11.55 ($C^{5/11}$), 10.60 ($C^{5/11}$); *Minor Isomer:* 171.4 (C^1), 169.5 (C^7), 155.9 (C^{14}), 144.14 ($C^{17/28}$), 144.10 ($C^{17/28}$), 141.6 ($C^{22/23}$), 141.5 ($C^{22/23}$), 135.5 (C^{30}), 128.6 (3C, $C^{20/25/31/32/33}$), 128.0 (2C, $C^{20/25/31/32/33}$), 127.5 (2C, $C^{20/25/31/32/33}$), 127.4 (2C, $C^{20/25/31/32/33}$), 124.6 ($C^{18/27}$), 124.3 ($C^{18/27}$), 120.3 ($C^{21/24}$), 120.2 ($C^{21/24}$), 68.0 (C^{15}), 67.1 (C^{29}), 63.8 (C^8), 56.1 (C^2), 47.4 (C^{16}), 37.4 (C^3), 32.3 (C^9), 29.7 (C^{13}), 25.0 (C^4), 24.2 (C^{10}), 15.6 ($C^{6/12}$), 15.5 ($C^{6/12}$), 11.4 ($C^{5/11}$), 10.9 ($C^{5/11}$); **HR-ESI-MS** (ESI^+): *m/z* = calc. for $[C_{35}H_{42}NaN_2O_5]^+$: 592.2986 ($[M+Na]^+$), found: 593.2963; *m/z* = calc. for $[C_{35}H_{42}KN_2O_5]^+$: 609.2725 ($[M+K]^+$), found: 609.2698, **IR:** (ATR) $\tilde{\nu}$ = 3341

(m, br, N-H), 3066 (w, CH_{Ar}), 3039 (w, CH_{Ar}), 2964 (m, CH_{Ar}), 2933 (w, CH_2), 2877 (w, CH_2), 1740 (m, C=O), 1670 (s, C=O), 1610 (w), 1521 (m), 1478 (w), 1452 (m), 1385 (m), 1339 (w), 1310 (m), 1249 (w), 1153 (s), 1115 (m), 996 (w), 962 (w), 907 (w), 840 (w), 758 (m), 740 (s), 698 (m).

Benzyl *N*-acetyl-*N*-methyl-L-isoleucyl-L-isoleucinate (Compound **S5**)

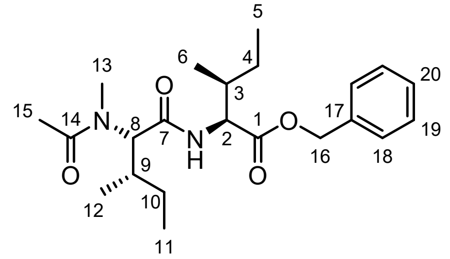

Compound **S4** (161 mg, 282 µmol, 1.00 equiv.) is dissolved in DMF (anhydrous, 2.26 mL, 125 mM). Piperidine (480 mg, 557 µL, 5.64 mmol, 20.0 equiv.) is added and the reaction mixture is stirred for 30 min at RT. All volatiles are removed *in vacuo* and the crude product was purified by column chromatography (silica, *n*-pentane/EtOAc = 95/5; R_f = 0.14 (*n*-pentane/EtOAc = 90/10) [UV]). After removal of all volatiles, the Fmoc-deprotected intermediate (92.3 mg, 265 µmol) is dissolved in CH_2Cl_2 (530 µL, 500 mM). Acetic anhydride (135 mg, 125 µL, 5.00 equiv.) and *N*,*N*-diisopropylethylamine (34.1 mg, 45.7 µL, 1.00 equiv.) are added and the reaction is stirred at RT for 3 h. The reaction mixture is diluted with $NaHCO_3$-solution (aqueous, saturated, 5 mL) and the aqueous layer is extracted with CH_2Cl_2 (3 x 10 mL). The combined organic layers are dried over Na_2SO_4, filtered and all volatiles are removed *in vacuo*. Purification by column chromatography (silica, *n*-pentane/EtOAc = 60/40) yielded **S5**.

Colorless solid (103 mg, 264 µmol, 94%); **TLC:** R_f = 0.54 *n*-pentane/EtOAc = 50/50); **^{1}H NMR** (500 MHz, $CDCl_3$, 298 K) δ (ppm) = 7.38 – 7.30 (m, 5H, $H^{18,19,20}$), 6.58 (d, $^3J_{H\text{-}H}$ 8.7 Hz, 1H, N*H*), 5.20 (d, $^2J_{H\text{-}H}$ = 12.2 Hz, 1H, H^{16a}), 5.11 (d, $^2J_{H\text{-}H}$ = 12.2 Hz, 1H, H^{16b}), 4.62 (d, $^3J_{H\text{-}H}$ = 11.4 Hz, 1H, H^8), 4.54 (dd, $^3J_{H\text{-}H}$ = 8.7, 5.0 Hz, 1H, H^2), 2.94 (s, 3H, H^{13}), 2.13 – 2.06 (m, 4H, H9,^{15}), 1.95 – 1.84 (m, 1H, H^3), 1.38 – 1.26 (m, 2H, H^4), 1.14 – 0.95 (m, 2H, H^{10}), 0.90 – 0.81 (m, 12H, $H^{5,6,11,12}$); **^{13}C{^{1}H} NMR** (126 MHz, $CDCl_3$, 298 K) δ (ppm) = 172.1 (C^{14}), 171.4 (C^1), 170.1 (C^7), 135.5 (C^{17}), 128.7 (3C, $C^{18/19,20}$), 128.5 (2C, $C^{18/19}$), 67.0 (C^{16}), 61.2 (C^8) 56.4 (C^2), 37.5 (C^3), 31.9 (C^9), 31.7 (C^{13}), 25.0 (C^{10}), 24.8 (C4), 22.2 (C^{15}), 15.7 ($C^{6/12}$), 15.6 ($C^{6/12}$), 11.6 ($C^{5/11}$), 10.6 ($C^{5/11}$); **HR-ESI-MS** (ESI^+): *m/z* = calc. for $[C_{22}H_{34}NaN_2O_4]^+$: 413.2411 ($[M+Na]^+$), found: 413.2403; *m/z* = calc. for $[C_{22}H_{34}KN_2O_4]^+$: 429.2150 ($[M+K]^+$), found: 429.2141; **IR:** (ATR) $\tilde{\nu}$ = 3323 (m, br. N-H), 3061 (w, CH_{Ar}), 2955 (m, CH_{Ar}), 2929 (m, CH_{Ar}), 2875 (w, CH_{Ar}), 1729 (s, C=O), 1673 (m, C=O), 1630 (s, C=O), 1541 (m), 1499 (w), 1457 (m), 1425 (w), 1405 (m), 1382 (m), 1362 (w), 1339 (w), 1314 (m), 1304 (m), 1290 (w), 1266 (m), 1225 (s), 1208 (s), 1156 (w), 1111 (w), 1055 (w), 1043 (w), 1019 (s), 979 (w), 961 (w), 946 (w), 937 (w), 912 (w), 854 (w), 842 (w), 833 (w), 872 (w), 771 (w), 754 (s), 741 (s), 699 (s), 683 (s).

Benzyl *N*-acetyl-*N*-methyl-L-isoleucyl-L-isoleucyl-L-threoninate (Compound **S6**)

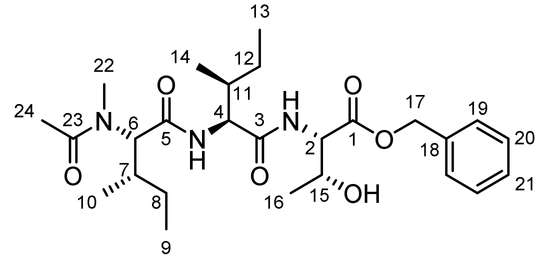

Compound **S5** (236.7 mg, 606 µmol, 1.00 equiv.) and palladium (10% (*w/w*) on carbon, 45.2 mg, 42.4 µmol, 0.07 equiv.) are suspended in methanol (anhydrous, 6.06 mL, 100 mM). The reaction mixture is degassed *via* freeze-pump-thaw (one cycle) and the atmosphere is exchanged for H_2. After stirring at RT for 4 h, the reaction mixture is filtered through a pad of Celite®, and all volatiles are removed *in vacuo* yielding the crude acid. *N*-Boc-L-Thr-OBn (187.5 mg, 606 µmol, 1.00 equiv.) is deprotected using HCl (4 M in dioxane, 758 µL, 5.00 equiv.). After stirring for 4 h at RT, all volatiles are removed *in vacuo* yielding the hydrochloride of H-Thr-OMe. The acid and the hydrochloride are dissolved in CH_2Cl_2 (anhydrous, 6.06 mL, 100 mM) and cooled to T = 0 °C. HOBt (92.8 mg, 606 µmol, 1.00 equiv.), HBTU (149 mg, 606 µmol, 1.00 equiv.) and *N*,*N*-diisopropylethylamine (157 mg, 211 µL, 1.21 mmol, 2.00 equiv.) are added successively. The reaction is allowed to warm up slowly to RT and stirred overnight. The reaction mixture is partitioned between CH_2Cl_2 (50 mL) and HCl (aqueous, 1 N, 20 mL), the aqueous layer is extracted with CH_2Cl_2 (3 x 20 mL), and the combined organic layers are washed with $NaHCO_3$-solution (aqueous, saturated, 20 mL), brine (20 mL), dried over Na_2SO_4 and filtered. All volatiles are removed *in vacuo* and purification by column chromatography (silica, hexanes/Ac = 70/30) yields **S6** (two isomers were observed in $CDCl_3$ solution [ratio of 87/13], presumably as a result of slight epimerization. The NMR assignments are reported for the major isomer).

Colorless solid (290 mg, 590 µmol, 97%); **TLC:** R_f = 0.21 (*n*-pentane/EtOAc = 50/50); **^{1}H NMR** (500 MHz, $CDCl_3$, 298 K) δ (ppm) = 7.38 – 7.30 (m, 5H, $H^{19,20,21}$), 6.83 (d, $^3J_{H-H}$ = 8.9 Hz, 1H, Ile-N*H*-Thr), 6.75 (d$^3J_{H-H}$ = 8.6 Hz, 1H, Ile-N*H*-Ile), 5.20 (d, $^2J_{H-H}$ = 12.3 Hz, 1H, H^{17a}), 5.16 (d, $^2J_{H-H}$ = 12.3 Hz, 1H, H^{17b}), 4.65 (dd, $^3J_{H-H}$ = 8.9, 2.6 Hz, 1H, H^2), 4.53 (d, $^3J_{H-H}$ = 11.4 Hz, 1H, H^6), 4.40 – 4.32 (m, 1H, H^{15}), 4.27 (dd, $^3J_{H-H}$ = 8.5, 6.9 Hz, 1H, H^4), 2.95 (s, 3H, H^{22}), 2.66 (d, $^3J_{H-H}$ = 5.7 Hz, 1H, O*H*), 2.15 – 2.10 (m, 1H, H^7), 2.09 (s, 3H, H^{24}), 2.02 – 1.94 (m, 1H, H^{11}), 1.44 – 1.38 (m, 1H, H^{12a}), 1.37 – 1.30 (m, 1H, H^{8a}), 1.18 (d, $^3J_{H-H}$ = 6.4 Hz, 1H, H^{16}), 1.16 – 1.06 (m, 1H, H^{12b}), 1.05 – 0.93 (m, 1H, H^{8b}), 0.91 – 0.82 (m, 12H, $H^{9,10,13,14}$); **^{13}C{^{1}H} NMR** (126 MHz, $CDCl_3$, 298 K) δ (ppm) = 172.4 (C^{23}), 171.5 (C^3), 170.6 (C^5), 170.5 (C^1), 135.3 (C^{18}), 128.8 (2C, $C^{19/20}$), 128.6 (C^{21}), 128.4 (2C, $C^{19/20}$), 68.4 (C^{15}), 67.5 (C^{17}), 62.2 (C^6), 58.1 (C^4), 57.4 (C^2), 36.2 (C^{11}), 32.6 (C^{22}), 31.7 (C^7), 24.8 ($C^{8/12}$), 24.7 ($C^{8/12}$), 22.2 (C^{24}), 20.1 (C^{16}), 15.7 ($C^{10/14}$), 15.6 ($C^{10/14}$), 11.2 ($C^{9/13}$), 10.6 ($C^{9/13}$)**; HR-ESI-MS** (ESI^+): *m/z* = calc. for $[C_{26}H_{41}N_3NaO_6]^+$: 514.2888 ($[M+Na]^+$), found: 514.2883; *m/z* = calc. for $[C_{26}H_{41}N_3KO_6]^+$: 530.2627 ($[M+K]^+$), found: 530.262; **IR:** (ATR) $\tilde{\nu}$ = 3312 (m, br. O-H, N-H), 2965 (m, CH_{Ar}), 2935 (w, CH_{Ar}), 2877 (w, CH_{Ar}), 1747 (m, C=O), 1626 (s, C=O), 1535 (m), 1456 (m), 1382 (w), 1311 (w), 1195 (w), 1153 (m), 1082 (w), 1016 (m), 828 (w), 736 (m), 697 (s).

Methyl (4*S*,7*S*,10*S*,13*S*)-4,7-di((*S*)-*sec*-butyl)-10-((*R*)-1-hydroxyethyl)-13-isobutyl-3,15,15-trimethyl-2,5,8,11,14-pentaoxo-3,6,9,12-tetraazahexadecan-16-oate (Compound **17**)

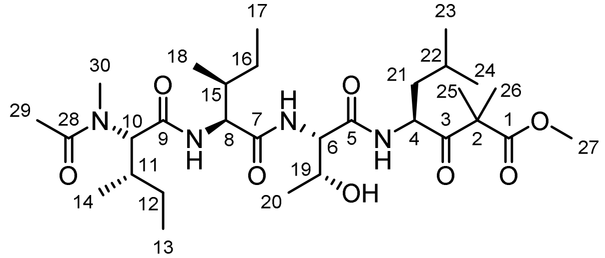

Compound **15** (29.0 mg, 227 µmol, 1.20 equiv.) is deprotected using TFA (10% (*v*/*v*) in CH_2Cl_2, [**15**] = 13 mM). The reaction mixture is stirred at RT for 2 h and all volatiles are removed *in vacuo*. Compound **S6** (92.9 mg, 189 µmol, 1.00 equiv.) and palladium (10% (*w*/*w*) on carbon, 20.1 mg, 18.9 µmol, 0.10 equiv.) are suspended in methanol (anhydrous, 3.78 mL, 50 mM). The reaction mixture is degassed *via* freeze-pump-thaw (one cycle) and the atmosphere is exchanged for H_2. After stirring at RT for 4 h, the reaction mixture is filtered through a pad of Celite®, and all volatiles are removed *in vacuo* yielding the crude acid. The acid, HOBT (28.9 mg, 189 µmol, 1.00 equiv.), and HBTU (71.7 mg, 189 µmol, 1.00 equiv.) are dissolved in CH_2Cl_2 (18.9 mL, 10 mm), cooled to T = 0 °C and *N*,*N*-diisopropylethylamine (65.8 µL, 48.9 mg, 378 µmol, 2.00 equiv.) is added slowly. The amine is dissolved in CH_2Cl_2 (5 mL) and added dropwise while stirring at this temperature. The reaction is stirred and allowed to warm up to RT overnight. The reaction mixture is partitioned between CH_2Cl_2 (50 mL) and HCl (aqueous, 1 N, 20 mL), the aqueous layer is extracted with CH_2Cl_2 (3 x 20 mL), and the combined organic layers are washed with $NaHCO_3$-solution (aqueous, saturated, 20 mL), brine (20 mL), dried over Na_2SO_4 and filtered. All volatiles are removed in vacuo and purification by column chromatography (silica, CH_2Cl_2/Ac = 90/10 → 70/30) yields **17** (two isomers were observed in $CDCl_3$ solution [ratio of 84/16], presumably as a result of slight epimerization. The NMR assignments are reported for the major isomer).

Colorless solid (50.7 mg, 84.7 µmol, 45%); **TLC:** R_f = 0.07 (CH_2Cl_2/Ac = 90/10) [CAM]; **^{1}H NMR** (500 MHz, $CDCl_3$, 298 K) δ (ppm) = 7.50 – 7.43 (m, 1H, Thr-N*H*-Leu), 7.40 – 7.31 (m, 1H, Ile-N*H*-Ile), 6.93 (d, $^3J_{H-H}$ = 7.9 Hz, 1H, Ile-N*H*-Thr), 4.94 (ddd, $^3J_{H-H}$ = 10.9, 9.1, 3.3 Hz, 1H, H^{4}), 4.68 (d, $^3J_{H-H}$ = 11.4 Hz, 1H, H^{10}), 4.45 (dd, $^3J_{H-H}$ = 7.9, 2.4 Hz, 1H, H^{6}), 4.28 – 4.22 (m, 2H, $H^{8,19}$), 3.72 (s, 3H, H^{27}), 2.99 (s, 3H, H^{30}), 2.12 (s, 3H, H^{29}), 2.10 – 2.04 (m, 1H, H^{11}), 2.01 – 1.94 (m, 1H, H^{15}), 1.59 – 1.51 (m, 1H, H^{22}), 1.46 – 1.30 (m, 10H, $H^{12a,16a21,25,26}$), 1.09 (d, $^3J_{H-H}$ = 6.5 Hz, 1H, H^{20}), 1.00 – 0.93 (m, 2H, $H^{12b,16b}$), 0.89 – 0.81 (m, 18H, $H^{13,14,17,18,23,24}$); **^{13}C{^{1}H} NMR** (126 MHz, $CDCl_3$, 298 K) δ (ppm) = 207.6 (C^{3}), 173.6 (C^{1}), 172.3 (C^{28}), 171.9 (C^{7}), 170.8 (C^{9}), 170.5 (C^{5}), 66.7 (C^{19}), 61.6 (C^{10}), 58.1 (C^{8}), 56.6 (C^{6}), 55.1 (C^{2}), 52.8 (C^{27}), 52.6 (C^{4}), 41.3 (C^{21}), 36.2 (C^{15}), 32.3 (C^{30}), 32.1 (C^{11}), 24.9 ($C^{12/16/22}$), 24.8 ($C^{12/16/22}$), 24.7 ($C^{12/16/22}$), 23.6 ($C^{23/24}$), 22.4 (C^{29}), 22.3 (C^{25}), 22.2 (C^{26}), 21.3 ($C^{23/24}$), 18.2 (C^{20}), 15.6($C^{14/18}$), 15.6 ($C^{14/18}$), 11.3 ($C^{13/17}$), 10.7 ($C^{13/17}$); **HR-ESI-MS** (ESI^+): *m/z* = calc. for $[C_{30}H_{54}N_4NaO_8]^+$: 621.3834 ($[M+Na]^+$), found: 621.3820; *m/z* = calc. for $[C_{30}H_{54}N_4KO_8]^+$: 637.3573 ($[M+K]^+$), found: 637.3557; **IR:** (ATR) $\tilde{\nu}$ = 3286 (s, br, O-H,N-H), 3066 (w, br), 2963 (m, C-H), 2935 (w, C-H), 2876 (w, C-H), 1746 (w, C=O), 1717 (w, C=O), 1626 (s, C=O), 1538 (m), 1464 (w), 1387 (w), 1314 (w), 1261 (w), 1215 (w), 1148 (m), 1107 (w), 1047 (w), 1017 (w), 933 (w), 876 (w), 833 (w), 689 (w).

Methyl (4*S*,7*S*,10*S*,13*S*)-4,7-di((*S*)-*sec*-butyl)-10-((*R*)-1-hydroxyethyl)-13-isobutyl-3-methyl-15,15-di(methyl-13C)-2,5,8,11,14-pentaoxo-3,6,9,12-tetraazahexadecan-16-oate (Compound [$^{13}C_2$]**17**)

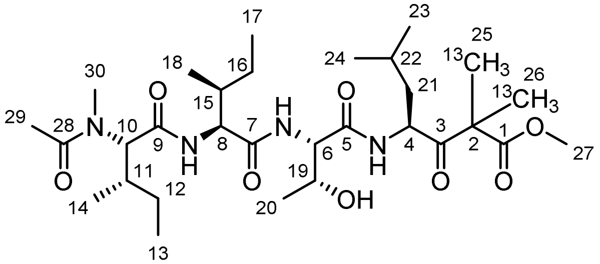

Compound [$^{13}C_2$]**15** (7.93 mg, 25 µmol, 1.12 equiv.) is deprotected using TFA (10% (*v/v*) in CH_2Cl_2, [[$^{13}C_2$]**15**] = 13 mM). The reaction mixture is stirred at RT for 2 h and all volatiles are removed *in vacuo*. Compound **S6** (11.0 mg, 22.4 µmol, 1.00 equiv.) and palladium (10% (*w/w*) on carbon, 2.38 mg, 2.24 µmol, 0.10 equiv.) are suspended in methanol (anhydrous, 500 µL, 50 mM). The reaction mixture is degassed *via* freeze-pump-thaw (one cycle) and the atmosphere is exchanged for H_2. After stirring at RT for 4 h, the reaction mixture is filtered through a pad of Ceilite®, and all volatiles are removed *in vacuo* yielding the crude acid. The acid and the amine are dissolved in CH_2Cl_2 (500 µL, 50 mM) and cooled to T = 0 °C. HOBT (3.43 mg, 22.4 µmol, 1.00 equiv.), *N*,*N*-diisopropylethylamine (7.79 µL, 5.78 mg, 44.7 µmol, 2.00 equiv.) and HBTU (8.48 mg, 22.4 µmol, 1.00 equiv.) are added successively while stirring. The reaction is allowed to warm up slowly to RT overnight. The reaction mixture is partitioned between CH_2Cl_2 (20 mL) and HCl (aqueous, 1 N, 10 mL), the aqueous layer is extracted with CH_2Cl_2 (3 x 20 mL), and the combined organic layers are washed with $NaHCO_3$-solution (aqueous, saturated, 20 mL), brine (20 mL), dried over Na_2SO_4 and filtered. All volatiles are removed *in vacuo* and purification by column chromatography (silica, CH_2Cl_2/Ac = 90/10 → 70/30) yields [$^{13}C_2$]**17** (two isomers were observed in $CDCl_3$ solution [ratio of 84/16], presumably as a result of slight epimerization. The NMR assignments are reported for the major isomer).

Colorless solid (7.80 mg, 13.0 µmol, 58%); **TLC:** R_f = 0.07 (CH_2Cl_2/Ac = 90/10) [CAM]; **^{1}H NMR** (500 MHz, $CDCl_3$, 298 K) δ (ppm) = 7.37 (d, $^3J_{H\text{-}H}$ = 9.10 Hz, 1H, Thr-N*H*-Leu), 7.28 – 7.23 (m, 1H, Ile-N*H*-Ile), 6.93 (d, $^3J_{H\text{-}H}$ = 7.85 Hz, 1H, Ile-N*H*-Thr), 4.94 (ddd, $^3J_{H\text{-}H}$ = 10.8, 9.1, 3.4 Hz, 1H, H^4), 4.66 (d, $^3J_{H\text{-}H}$ = 11.4 Hz, 1H, H^{10}), 4.41 (dd, $^3J_{H\text{-}H}$ = 7.9, 2.5 Hz, 1H, H^6), 4.27 – 4.22 (m, 2H, $H^{8,19}$), 3.72 (s, 3H, H^{27}), 2.98 (s, 3H, H^{30}), 2.12 (s, 3H, H^{29}), 1.42 (dd, $^1J_{C\text{-}H}$ = 130.4 Hz, $^2J_{H\text{-}H}$ = 4.9 Hz, 3H, $H^{25/26}$), 1.40 (dd, $^1J_{C\text{-}H}$ = 130.1 Hz, $^2J_{H\text{-}H}$ = 4.5 Hz, 3H, $H^{25/26}$), 1.47 – 1.32 (m, 7H, $H^{12,16,21,22}$), 1.09 (d, $^3J_{H\text{-}H}$ = 6.46 Hz, 1H, H^{20}), 0.92 – 0.81 (m, 18H, $H^{13,14,17,18,23,24}$); **^{13}C{^{1}H} NMR** (126 MHz, $CDCl_3$, 298 K) δ (ppm) = 207.6 (s, C^3), 173.6 (s, C^1), 172.3 (s, C^{28}), 172.0 (s, C^7), 170.8 (s, 1C, $C^{9/5}$), 170.5 (s, 1C, $C^{9/5}$), 66.7 (s, C^{19}), 61.7 (s, C^{10}), 58.1 (s, C^8), 56.7 (s, C^6), 55.1 (t, $^2J_{C\text{-}C}$ = 34.3 Hz, C^2), 52.8 (s, C^{27}), 52.6 (s, C^4), 41.3 (s, C^{21}), 36.2 (s, $C^{11/15}$), 32.0 (s, C^{30}), 29.4 (s, $C^{11/15}$), 24.8 (s, 1C, $C^{12/16/22/23/24}$), 24.8 (s, 1C, $C^{12/16/22/23/24}$), 24.7 (s, 1C, $C^{12/16/22/23/24}$), 23.6 (s, 1C, $C^{12/16/22/23/24}$), 22.4 (s, $C^{25/26}$), 22.3 (s, $C^{25/26}$), 22.1 (s, C^{29}), 21.3 (s, 1C, $C^{12/16/22/23/24}$), 18.2 (s, C^{20}), 15.7 (s, $C^{14/18}$), 15.6 (s, $C^{14/18}$), 11.3 (s, $C^{13/17}$), 10.7 (s, $C^{13/17}$); **HR-ESI-MS** (ESI^+): *m/z* = calc. for $[C_{28}{}^{13}C_2H_{54}N_4NaO_8]^+$: 623.3901 ($[M+Na]^+$), found: 623.3887; *m/z* = calc. for $[C_{28}{}^{13}C_2H_{54}N_4KO_8]^+$: 639.3640 ($[M+K]^+$), found: 639.3626; **IR:** (ATR) $\tilde{\nu}$ = 3287 (m, br, O-H, N-H), 3064 (w, br), 2963 (m, C-H), 2934 (w,), 2876 (w, C-H), 1718 (w, C=O), 1630 (s, C=O), 1539 (m), 1466 (w), 1386 (w), 1315 (w), 1272 (w), 1219 (w), 1144 (w), 1015 (w), 872 (w), 689 (w) cm^{-1}.

(4*S*,7*S*,10*S*)-4,7-di((*S*)-*sec*-Butyl)-10-((*R*)-1-hydroxyethyl)-13-isobutyl-3,15,15-trimethyl-2,5,8,11,14-pentaoxo-3,6,9,12-tetraazahexadecan-16-oic acid (Compounds **7** and *epi*-**7**)

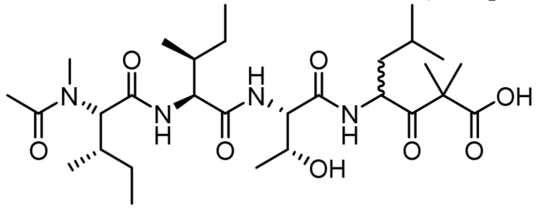

The saponification of Compound **17** is performed according to the saponification of compound **15**. Compound **17** (8.00 mg, 13.4 µmol, 1.00 equiv.) is dissolved in THF (anhydrous, 66.8 µL, 200 mM) and cooled to T = 0 °C and a solution of LiOH (352 µg, 14.7 µmol, 1.10 equiv.) in H_2O (66.8 µL, 200 mM) is added dropwise (a stock solution of 3.52 mg LiOH in 668 µL H_2O is prepared). After stirring for 25 min at T = 0 °C the reaction mixture is poured on ice and the pH is carefully adjusted to pH = 9 with LiOH using the prepared stock solution. The cold, aqueous layer is extracted with CH_2Cl_2 (3 x 20 mL) and then acidified with H_2SO_4 (0.5 N) to pH = 3. The cold aqueous layer is extracted with CH_2Cl_2 (4 x 20 mL) and the combined organic layers from the second extraction are combined, dried over Na_2SO_4 and filtered. All volatiles are removed *in vacuo* at T = 0 °C to avoid decarboxylation, and the β-ketoacid is directly used in the assays.

Colorless solid (4.20 mg, 7.18 µmol, 54%); **HR-ESI-MS** (ESI^-): *m/z*= calc. for $[C_{29}H_{51}N_4O_8]^-$: 583.3712 ($[M\text{-}H]^-$), found: 583.3716; *m/z*= calc. for $[C_{28}H_{51}N_4O_6]^-$: 539.3814 ($[M\text{-}CO_2\text{-}H]^-$), found: 539.3817.

(4*S*,7*S*,10*S*)-4,7-di((*R*)-*sec*-butyl)-10-((*R*)-1-hydroxyethyl)-13-isobutyl-3-methyl-15,15-di(methyl-13C)-2,5,8,11,14-pentaoxo-3,6,9,12-tetraazahexadecan-16-oic acid (Compounds [$^{13}C_2$]**7** and *epi*-[$^{13}C_2$]**7**)

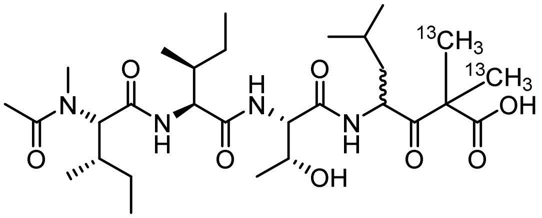

Saponification of Compound [$^{13}C_2$]**17** is performed according to the saponification of compound **15**. Compound [$^{13}C_2$]**17** (4.8 mg, 7.99 µmol, 1.00 equiv.) is dissolved in THF (anhydrous, 40.0 µL, 200 mM) and cooled to T = 0 °C and a solution of LiOH (210 µg, 8.79 µmol, 1.10 equiv.) in H_2O (40.0 µL, 200 mM) is added dropwise (a stock solution of 2.10 mg LiOH in 400 µL H_2O is prepared). After stirring for 25 min at T = 0 °C the reaction mixture is poured on ice and the pH is carefully adjusted to pH = 9 with LiOH using the prepared stock solution. The cold, aqueous layer is extracted with CH_2Cl_2 (3 x 20 mL) and then acidified with H_2SO_4 (0.5 N) to pH = 3. The cold aqueous layer is extracted with CH_2Cl_2 (4 x 20 mL) and the combined organic layers from the second extraction are combined, dried over Na_2SO_4 and filtered. All volatiles are removed *in vacuo* at T = 0 °C to avoid decarboxylation, and the β-ketoacid is directly used in the assays.

Colorless solid (1.90 mg, 3.24 µmol, 41%); HR-ESI-MS (ESI$^+$): *m/z* = calc. for $[C_{27}{}^{13}C_2H_{53}N_4O_8]^+$: 587.3925 ($[M+H]^+$), found: 587.3918; *m/z* = calc. for $[C_{27}{}^{13}C_2H_{52}NaN_4O_8]^+$: 609.3744 ($[M+Na]^+$), found: 609.3735.

3-Butyl-10-methylbenzo[*g*]pteridine-2,4(3*H*,10*H*)-dione (Compound **12**)

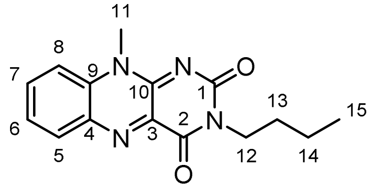

This is a known compound and it was prepared according to the published methods. The analytic data is in accordance with the literature.[24]

Yellow solid: 267 mg (0.94 mmol, 36%); **TLC:** $R_f = 0.29$ (CH_2Cl_2/Ac = 90/10) [coloured compound]; **^{1}H NMR** (500 MHz, $CDCl_3$, 298 K) δ (ppm) = 8.31 (dd, $^3J_{H\text{-}H} = 8.13$ Hz, $^4J_{H\text{-}H} = 1.57$ Hz, 1H, H^5), 7.90 (ddd, $^3J_{H\text{-}H} = 8.70$ Hz, $^3J_{H\text{-}H} = 7.16$ Hz, $^4J_{H\text{-}H} = 1.53$ Hz, 1H, H^7), 7.66 – 7.61 (m, 2H, $H^{6,8}$), 4.14 – 4.07 (m, 5H, $H^{11,12}$), 1.70 (m, 2H, H^{13}), 1.42 (*virt.* sext, $^3J_{H\text{-}H} \approx {}^3J_{H\text{-}H'} = 7.45$ Hz, 2H, H^{14}), 0.95 (t, $^3J_{H\text{-}H} = 7.36$ Hz, 3H, H^{15}); **^{13}C{^{1}H} NMR** (126 MHz, $CDCl_3$, 298 K) δ (ppm) = 159.6 (C^1), 155.6 (C^2), 149.4 (C^{10}), 137.3 (C^3), 135.9 (C^7), 135.8 (C^4), 133.5 (2C, $C^{5,9}$), 126.7 (C^6), 115.2 (C^8), 42.1 (C^{12}), 32.1 (C^{11}), 30.0 (C^{13}), 20.3 (C^{14}), 14.0 (1C, C^{15}).

Saponification Study

The epimerisation of the leucine α-C*H* stereocenter was studied with head group **15** under saponification conditions. The experiments were performed in NMR tubes with 15.9 μmol (5.0 mg) of substrate **15**. The material was dissolved in THF-d_8/D_2O (2:1) or THF-d_8/H_2O (2:1), and the base was added subsequently. Independent studies with the bases LiOH and K_2CO_3 were performed (with 1.5 equiv. of base in both cases).

The saponification with lithium hydroxide occurred rapidly. In THF-d_8/D_2O, full consumption of the methyl ester starting material **15** was observed within 10 min (Fig. S7), and a 60:40 ratio of β-ketoacid **10** and ketone **11** was detected. The formation of MeOH (proton signal at δ = 3.23 ppm) confirmed saponification. The decarboxylation was complete within 5 h and the spectrum at that point only showed ketone **11**. Complete deuteration at the leucine α-C*H* position was confirmed by the ^{1}H,^{13}C-HSQC contact with the residual proton signal (δ = 4.23 ppm // δ = 56.3 ppm, integral < 0.05 H). Complete deuteration of the isopropyl methine C*H* position was also confirmed since two singlets were observed (δ = 0.98 and 0.94 ppm // δ = 17.4 and 18.1 ppm). In THF-d_8/H_2O, similar saponification was observed (Fig. S8) and full conversion to ketone **11** occurred within 5 h of reaction time without deuteration. The ^{1}H,^{13}C-HSQC contacts confirmed the leucine α-C*H* position and the isopropyl methine C*H* position.

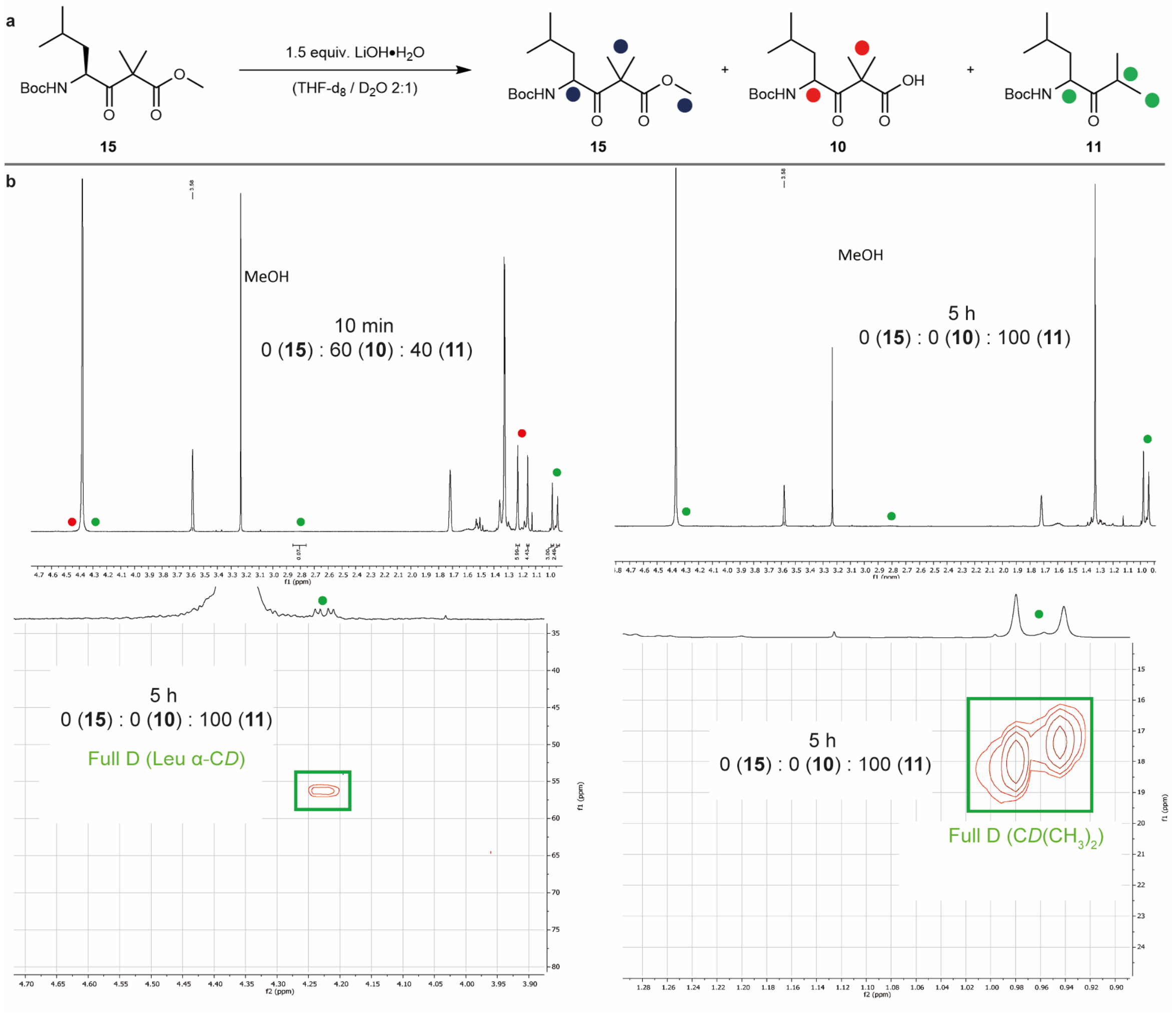

Figure S7. Saponification study of compound **15** with LiOH in THF-d8/D_2O.

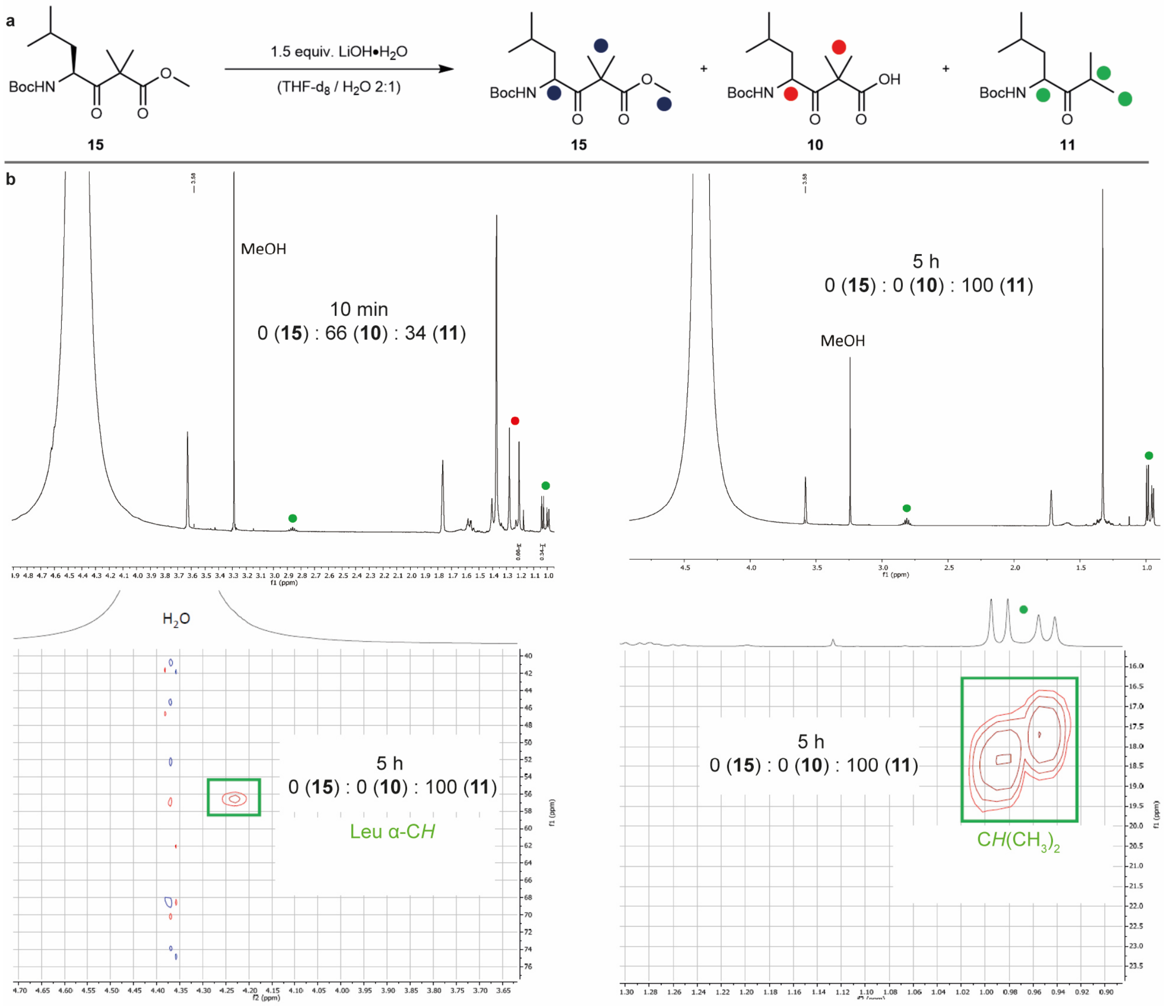

Figure S8. Saponification study of compound **15** with LiOH in THF-d8/H_2O.

The conversion of methyl ester **15** under milder basic conditions with K_2CO_3 was then studied. Indeed, saponification was much slower under these conditions (in THF-d_8/D_2O). After 10 min reaction time, 24% of the ketoacid **10** had formed, while 76% of ester substrate **15** remained (Fig. S9). Relative integration of the signals at 4.43 ppm (**10**, leucine α-C*H*) and at 1.22 ppm (**10**, methyl CH_3) revealed a deuteration degree of approx. 40%. After prolonged reaction time of 30 min, the conversion to β-ketoacid **10** had reached 39% and the deuteration degree (**10**, leucine α-C*H*) was determined as 62%. After 4 h, β-ketoacid **10** (60%) was the major species with a deuteration degree of 96% (**10**, leucine α-C*H*). At this point, already 13% of the decarboxylated ketone **11** had formed, which was fully deuterated at the leucine α-C*H* and the isopropyl methine C*H* positions. After 20 h of reaction time, the product ratio was determined as 10 (**15**) : 58 (**10**) : 32 (**11**). The leucine α-C*H* positions in both **10** and **11**, and also the isopropyl methine C*H* position in **11** were fully deuterated. Analysis of the $^1H,^{13}C$-HSQC spectrum after 4 h (Fig. S10) confirmed the assignment of the leucine α-C*H* positions of **15**, **10**, and **11**, and also of the methyl CH_3 positions of all three species. When the analogous reaction was performed in THF-d_8/H_2O, a ratio of 37 (**15**) : 50 (**10**) : 13 (**11**) was determined after 8 h of reaction time. In this experiment, the $^1H,^{13}C$-HSQC analysis confirmed that no deuteration took place at either position of β-ketoacid **10** or ketone **11**.

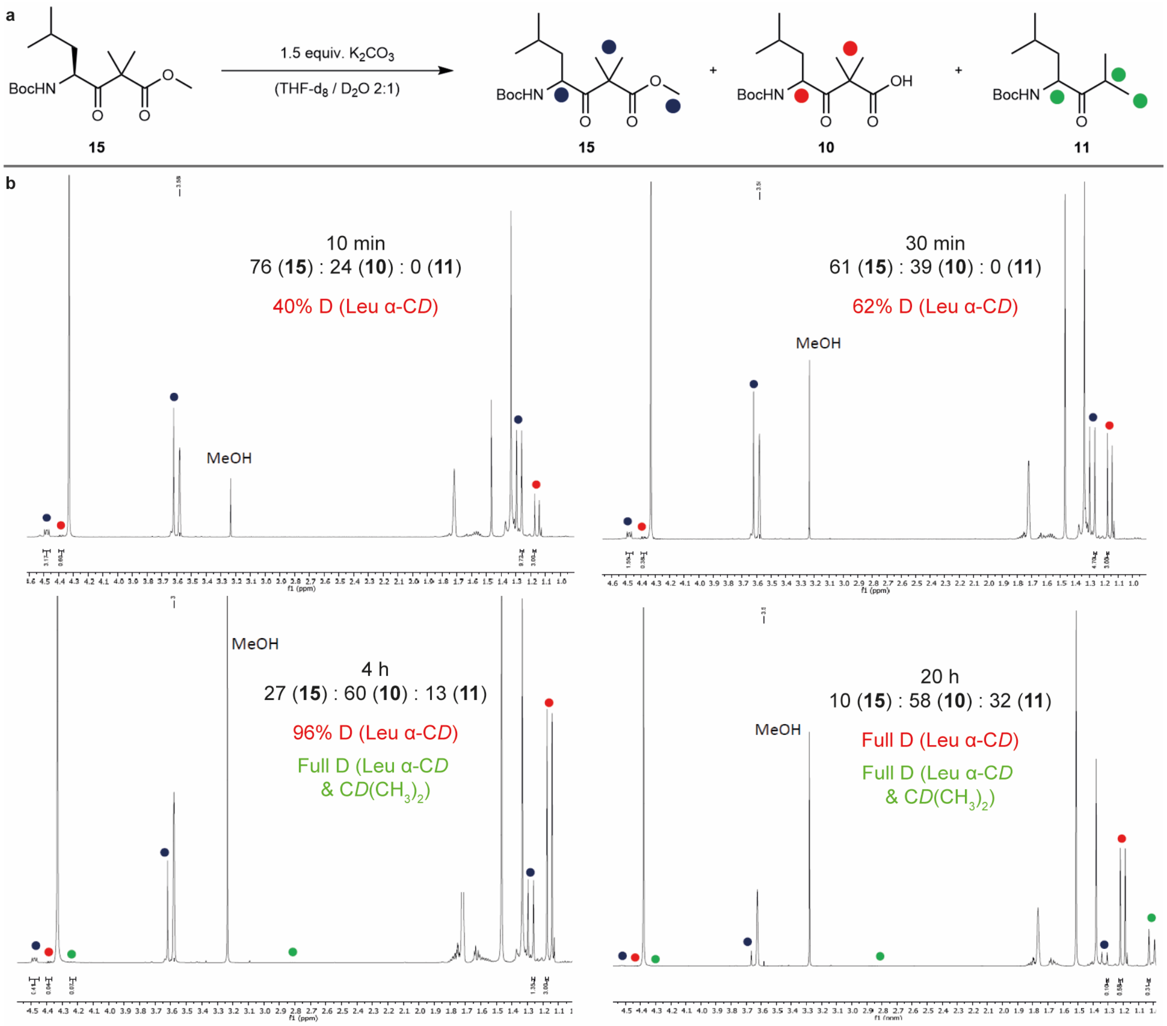

Figure S9. Saponification study of compound **15** with K_2CO_3 in THF-d8/D_2O.

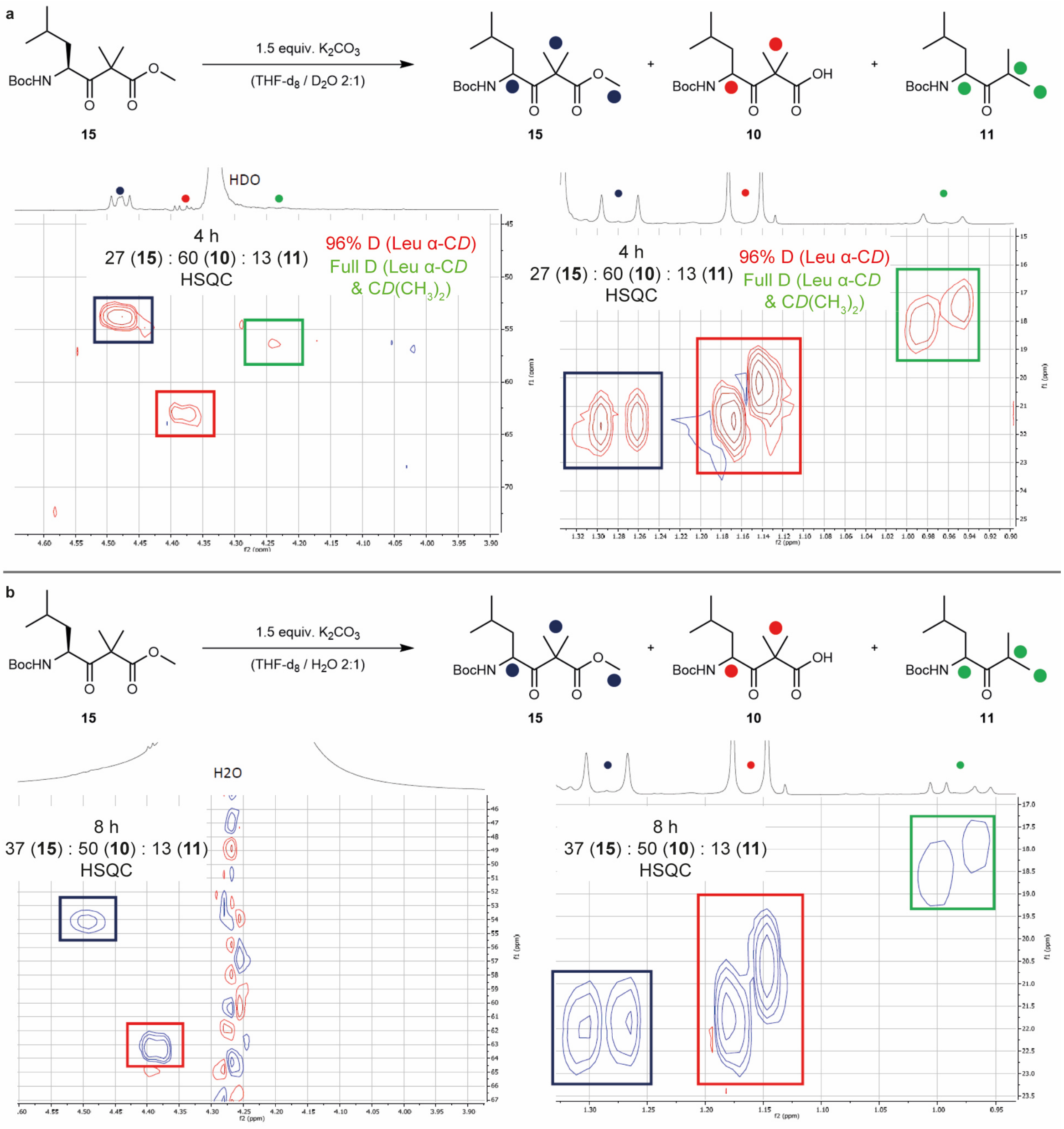

Figure S10. Saponification study of compound **15** with K_2CO_3 in THF-d8/D_2O and THF-d8/H_2O.

Stoichiometric Flavin-Mediated Reactions

Flavin-Mediated Oxidation

Flavin **12** (7.11 mg, 25.0 μmol, 1.00 equiv.) and ketoacid **10** (7.53 mg, 25.0 μmol, 1.00 equiv.) are placed in a crimp-cap vial equipped with a magnetic stirring bar. The vial is sealed with a septum safety cap and the atmosphere is exchanged by evacuating the vial and backfilling it with argon. The reaction components are suspended in acetonitrile (anhydrous, degassed, 500 μL, 50 mM). The reaction mixture is irradiated with blue LED light (λ_{max} = 451 nm, 1 W) at T = 15 °C under stirring while manually swiveling the reaction vial every hour to ensure even distribution of the insoluble reaction components. After 8 h the reaction mixture is diluted with CH_2Cl_2 and transferred into a round-bottom flask, rinsing the reaction vial with CH_2Cl_2. All volatiles are removed under reduced pressure, and $CDCl_3$ with trimethyl trimesic acid (8.33 μmol, the integral of the signal at δ = 8.82 ppm is set to 1.00, signal marked yellow) as internal standard is added. Yields determined by NMR spectroscopy (Fig. S11): 56% *α,β*-unsaturated ketone **14** (signals marked green). The reaction mixture contains 19% of ketone **11** (signal marked red) resulting from decarboxylation. Purification by column chromatography yields an inseparable mixture of **14** and **11** (Fig. S12 and S13). Combined yield: 3.8 mg. Calculated total yield of isolated material: 43% **14**.

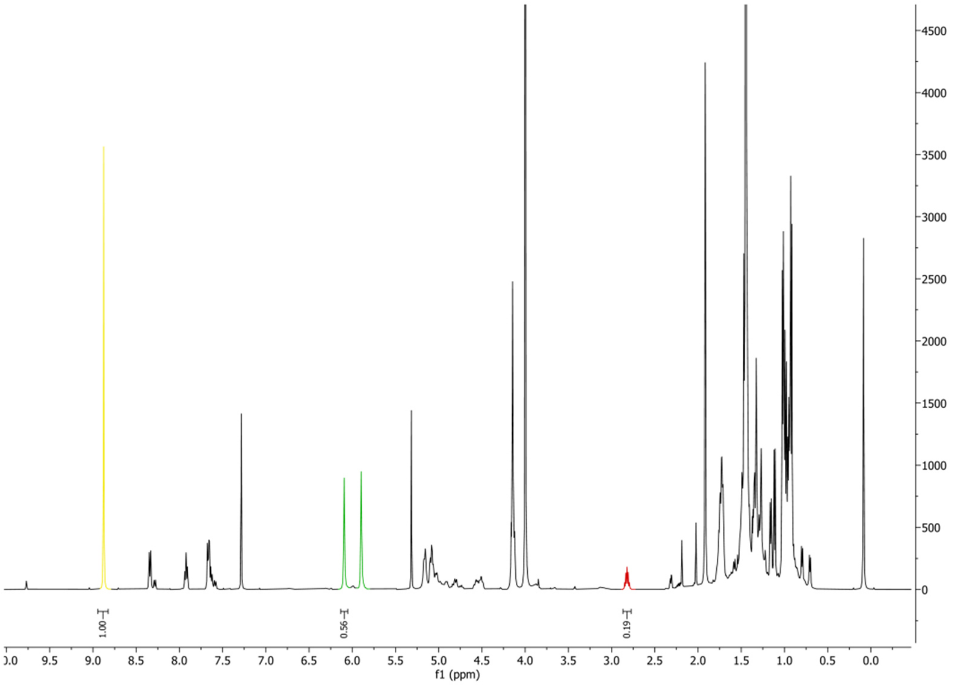

Figure S11. Crude ^{1}H NMR spectrum in $CDCl_3$ with trimethyl trimesic acid as internal standard. Signals relevant for assignment in product mixture are color-coded.

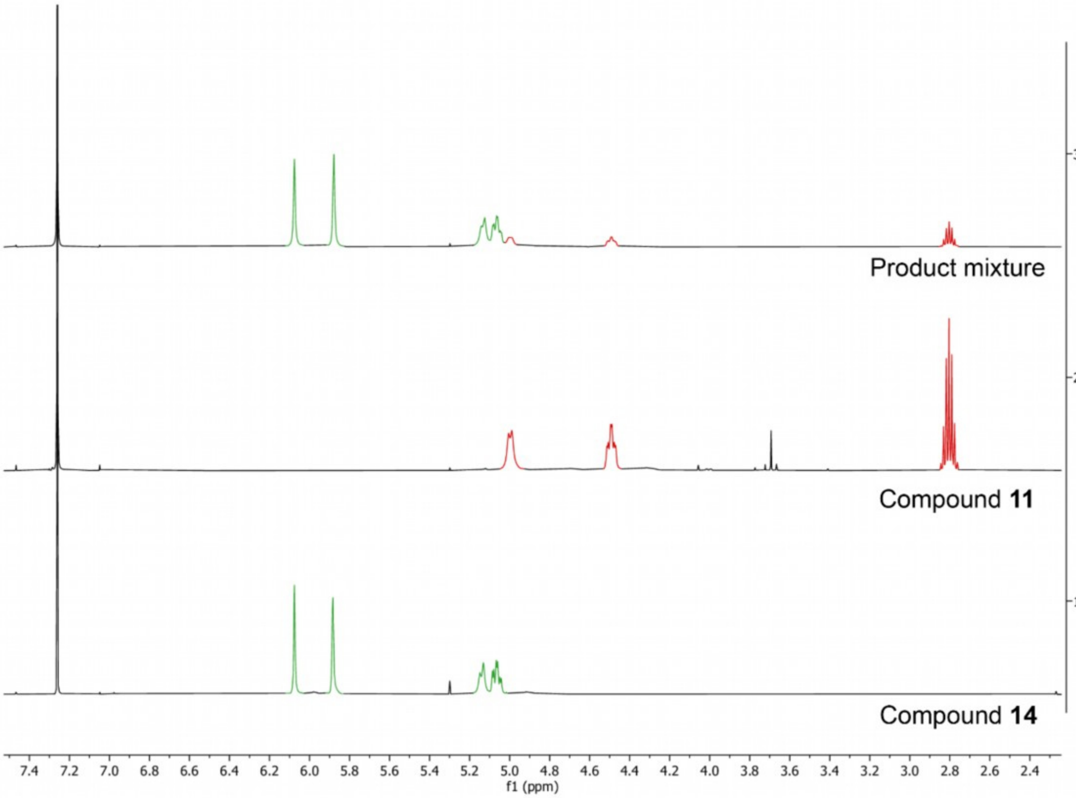

Figure S12. Stacked ^{1}H NMR spectra of α,β-unsaturated ketone **14** (spectrum #1), ketone **11** (spectrum #2), and the obtained product mixture (spectrum #3). Signals relevant for assignment are color-coded.

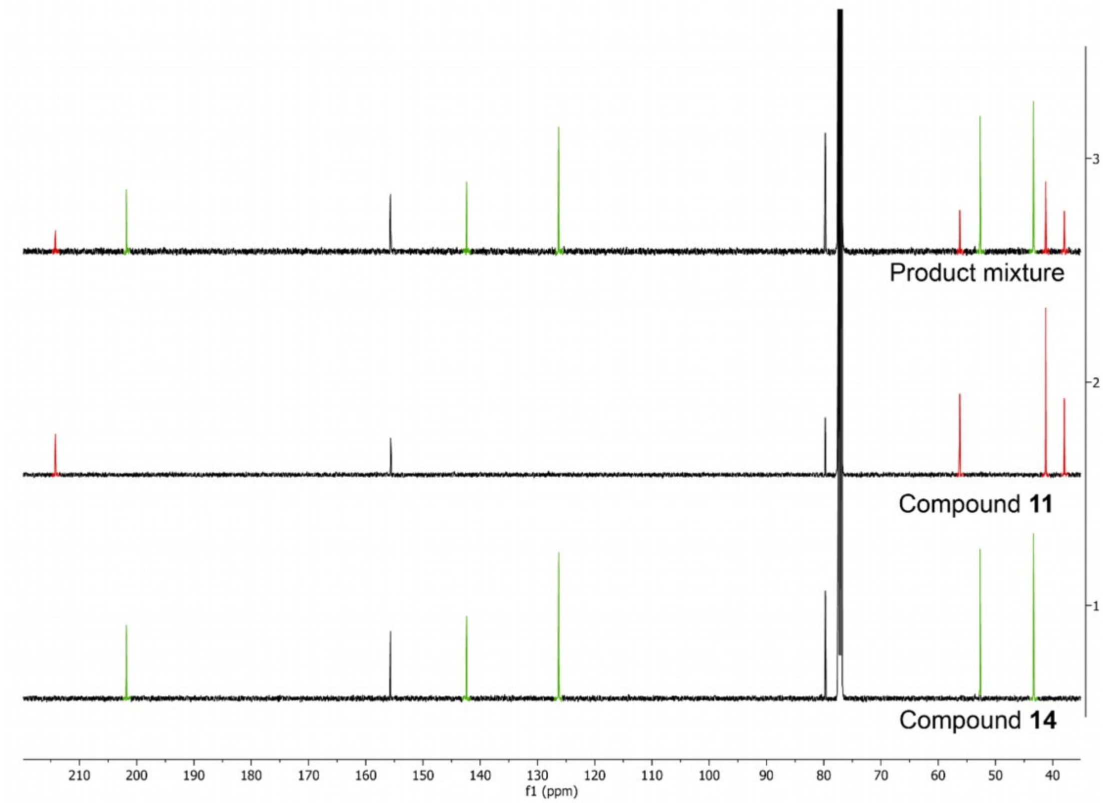

Figure S13. Stacked $^{13}C\{^{1}H\}$ NMR spectra of α,β-unsaturated ketone **14** (spectrum #1), ketone **11** (spectrum #2), and the obtained product mixture (spectrum #3). Signals relevant for assignment in product mixture are color-coded.

Flavin-Mediated Oxidation-Epoxidation Sequence

Flavin **12** (7.11 mg, 25.0 µmol, 1.00 equiv.) and ketoacid **10** (7.53 mg, 25.0 µmol, 1.00 equiv.) are placed in a crimp-cap vial equipped with a magnetic stirring bar. The vial is sealed with a septum safety cap and the atmosphere is exchanged by evacuating the vial and backfilling it with argon. The reaction components are suspended in acetonitrile (anhydrous, degassed, 500 µL, 50 mM). The reaction mixture is irradiated with blue LED light (λ_{max} = 451 nm, 1 W) at T = 15 °C under stirring while manually swiveling the reaction vial every hour to ensure even distribution of the insoluble reaction components. After 8 h irradiation, the crimp-cap vial is opened, and *Hantzsch* Ester (12.7 mg, 50.0 µmol, 2.00 equiv.), Cs_2CO_3 (12.2 mg, 37.5 µmol, 1.50 equiv.) and a mixture of MeOH (anhydrous, 167 µL, filtered through a 0.22 µm Millex® syringe filter) and H_2O (0.18 µL) from a previously prepared stock solution is added to the reaction mixture. The crimp-cap vial is sealed again and the atmosphere is exchanged for O_2 by bubbling through the solution for 30 sec. The vial cap is sealed with parafilm and the reaction is stirred at 40 °C overnight. The reaction is diluted with CH_2Cl_2, transferred to a round bottom flask, and all volatiles are removed under reduced pressure. Due to the decomposition of epoxyketone **16** on silica over time, then the crude mixture is filtered through a short column (silica, *n*-pentane/EtOAc = 95/5).

A mixture of epoxyketone **16**, ketone **11**, *α,β*-unsaturated ketone **14,** and diethyl 2,6-dimethylpyridine-3,5-dicarboxylate (resulting from *Hantzsch* ester oxidation) is obtained in a combined yield of 1.6 mg. Calculated total yield of epoxyketone **16:** 8%.

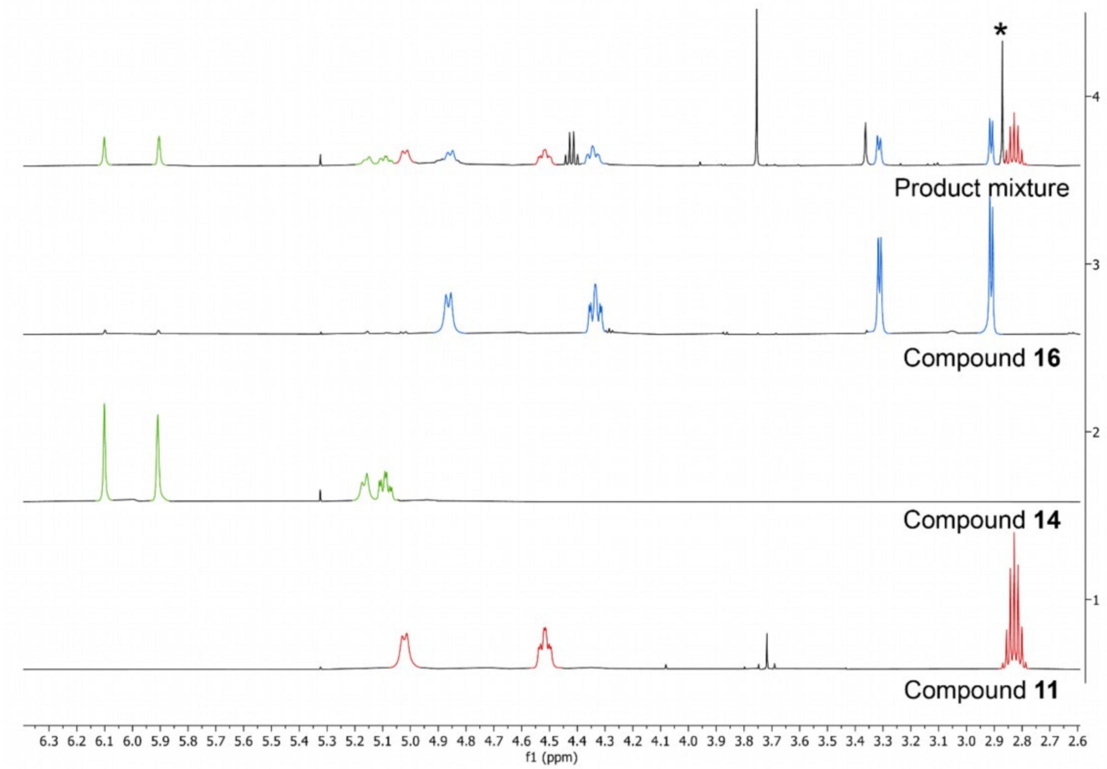

Figure S14. Stacked ^{1}H NMR spectra of ketone **11** (spectrum #1), α,β-unsaturated ketone **14** (spectrum #2), epoxyketone **16** (spectrum #3) and the obtained product mixture (spectrum #4). Signals relevant for assignment in product mixture are color-coded.

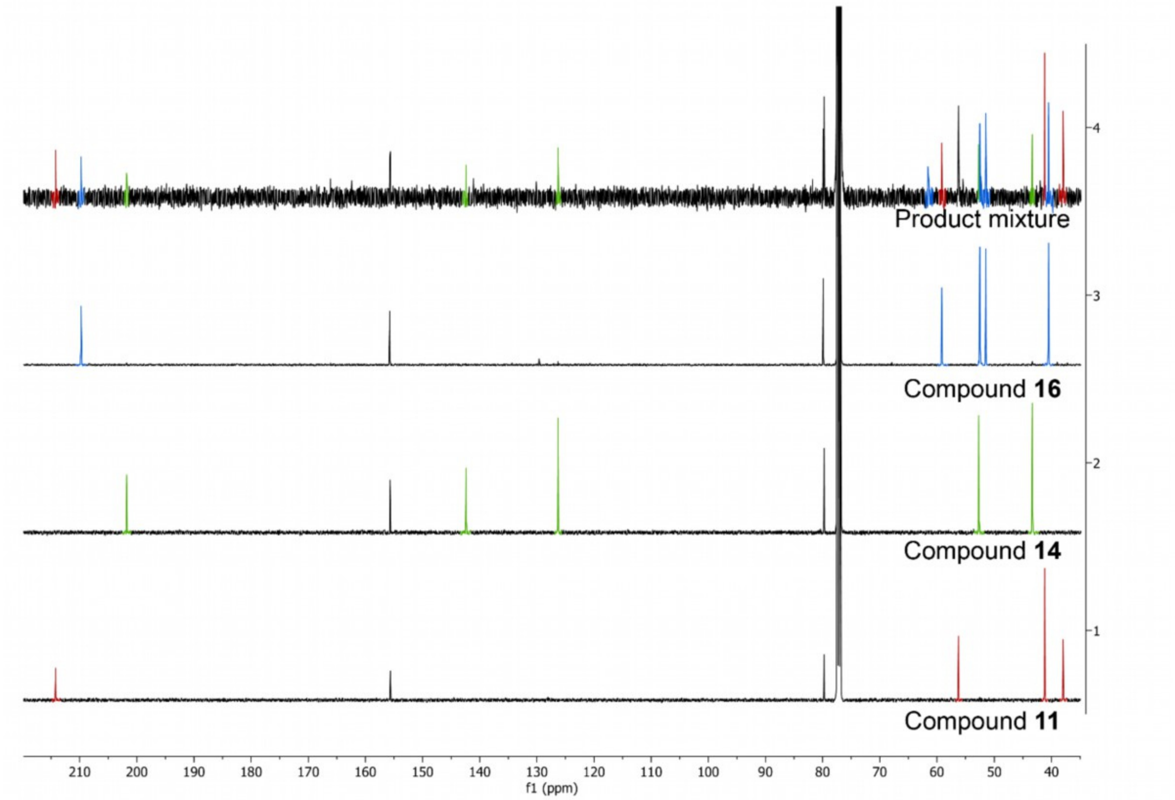

Figure S15. Stacked $^{13}C\{^{1}H\}$ NMR spectra of ketone **11** (spectrum #1), α,β-unsaturated ketone **14** (spectrum #2), epoxide **16** (spectrum #3), and the obtained product mixture (spectrum #4). Signals relevant for assignment in product mixture are color-coded.

Catalytic Flavin-Mediated Reactions

Flavin-Catalyzed Oxidation

A crimp-cap vial equipped with a magnetic stirring bar is filled with the acid **10** (15.1 mg, 50.0 µmol, 1.00 equiv.), flavin **12** (1.42 mg, 5.00 µmol, 0.10 equiv.), $K_2S_2O_8$ (13.5 mg, 50.0 µmol, 1.00 equiv.) and Na_2HPO_4 (17.7 mg, 125 µmol, 2.50 equiv.). The vial is sealed with a septum safety cap and the atmosphere is exchanged by evacuating the vial and backfilling it with argon. The reaction components are suspended in acetonitrile (250 µL, anhydrous, 200 mM) and the reaction mixture is degassed three times by freeze-pump-thaw. The reaction mixture is irradiated with blue LED light (λ_{max} = 451 nm, 1 W) at T = 15 °C under stirring while manually swiveling the reaction vial every 30 min to ensure even distribution of the insoluble reaction components. After 8 h the reaction mixture is diluted with CH_2Cl_2 and transferred into a round-bottom flask, rinsing the reaction vial with CH_2Cl_2. All volatiles are removed under reduced pressure and $CDCl_3$ with trimethyl trimesic acid (8.33 µmol, the integral of the signal at δ = 8.82 ppm is set to 0.50, signal marked yellow) as internal standard is added (Fig. S16). Yield determined by NMR-Spectroscopy: 33% *α,β*-unsaturated ketone **14** (signals marked green). The reaction contained 13% of ketone **11** (signal marked red) resulting from decarboxylation.

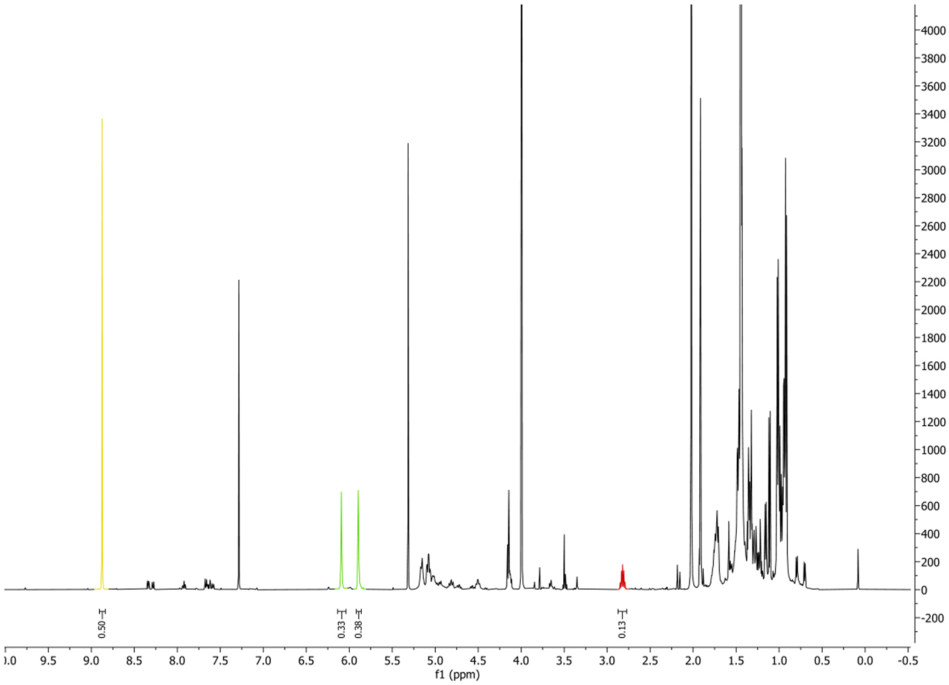

Figure S16. Crude ^{1}H NMR spectrum in $CDCl_3$ with trimethyl trimesic acid as internal standard. Signals relevant for assignment in product mixture are color-coded.

Flavin-Catalyzed Oxidation-Epoxidation Sequence

A crimp-cap vial equipped with a magnetic stirring bar is filled with the ketoacid **10** (15.1 mg, 50.0 µmol, 1.00 equiv.), flavin **12** (1.42 mg, 5.00 µmol, 0.10 equiv.), $K_2S_2O_8$ (13.5 mg, 50.0 µmol, 1.00 equiv.) and Na_2HPO_4 (17.7 mg, 125 µmol, 2.50 equiv.). The vial is sealed with a septum safety cap and the atmosphere is exchanged by evacuating the vial and backfilling it with argon. The reaction components are suspended in acetonitrile (250 µL, anhydrous, 200 mM) and the reaction mixture is degassed three times by freeze-pump-thaw. The reaction mixture is irradiated with blue LED light (λ_{max} = 451 nm, 1 W) at T = 15 °C under stirring while manually swiveling the reaction vial every 30 min to ensure even distribution of the insoluble reaction components. After 8 h irradiation, the crimp-cap vial is opened, and *Hantzsch* Ester (25.4 mg, 100 µmol, 2.00 equiv.), Cs_2CO_3 (24.4 mg, 75.0 µmol, 1.50 equiv.) and a mixture of MeOH (anhydrous, 83 µL, filtered through a 0.22 µm Millex® syringe filter) and H_2O (0.09 µL, 10 mol%) from a previously prepared stock solution is added to the reaction mixture. The crimp-cap vial is sealed again and the atmosphere is exchanged for O_2 by bubbling through the solution for 30 sec. The vial cap is sealed with parafilm and the reaction is stirred at 40 °C overnight. The reaction is diluted with CH_2Cl_2, transferred to a round bottom flask, and all volatiles are removed under reduced pressure. Due to the decomposition of epoxyketone **16** on silica over time, the crude mixture is filtered through a short column (silica, *n*-pentane/EtOAc = 95/5). Epoxyketone **16** (Fig. S17 and S18) is obtained in 8% yield (1.10 mg, 4.05 µmol).

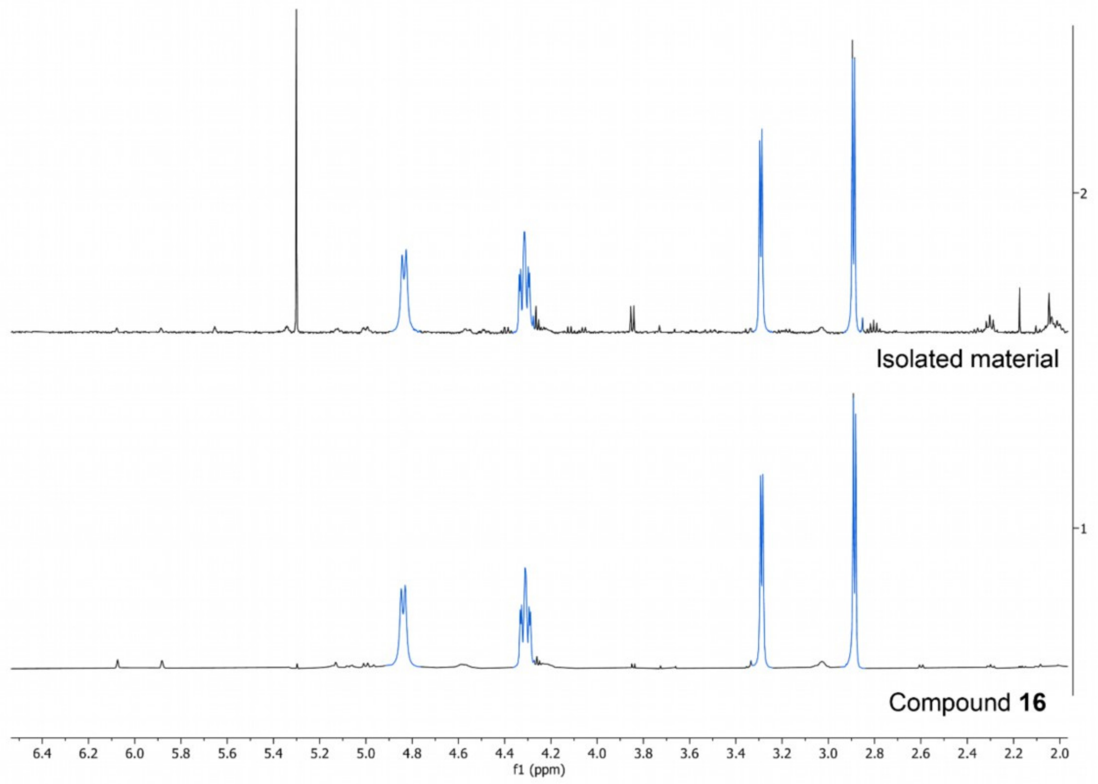

Figure S17. Stacked ^1H NMR spectra of epoxide **16** (spectrum #1) and the isolated product (spectrum #2). Signals relevant for assignment in isolated product are color-coded.

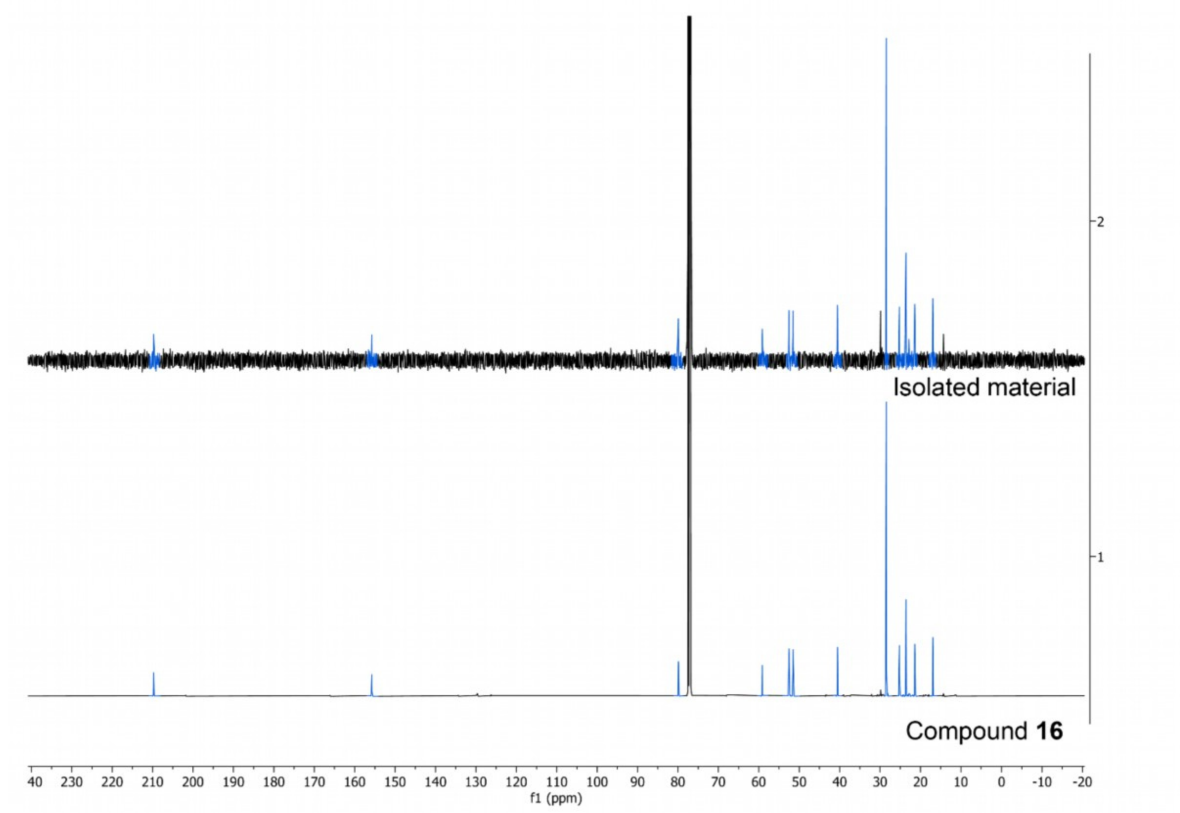

Figure S18. Stacked $^{13}C\{^1H\}$ NMR spectra of epoxide **16** (spectrum #1) and the isolated product (spectrum #2). Signals relevant for assignment in isolated product are color-coded.

Control Experiments for the Flavin-Mediated Oxidation

Table S3. Control experiments for the oxidation of compound 10 with flavin 12. Yields were determined by NMR spectroscopy *versus* internal standard.

#	Subst.	Flavin [mol%]	Oxidant (equiv.)	Additive (equiv.)	Time [h]	EPK 16 [%]	USK 14 [%]	SM [%]	Ketone 11 [%]
**1**	Ester **15**	100	-	-	3	-	-	97	-
**2**	Keton **11**	100	-	-	3	-	-	88	88
**3**	Acid **10**	100	-	-	3 (dark)	-	-	59	17
**4**	Acid **10**	-	-	-	3	-	-	41	38
**5**	Acid **10**	100	-	-	3	-	15	39	13
**6**	Acid **10**	-	$K_2S_2O_8$ (1.00)	NaH_2PO_4 (2.50)	8	-	-	trace	85
**7**[a]	Acid **10**	10	-	-	3	-	trace	29	trace

[a] Reaction was performed under O_2; EPK = α,β-Epoxyketone; USK = α,β-Unsaturated Ketone; SM = Starting Material.

NMR Spectra

Compound **S1**

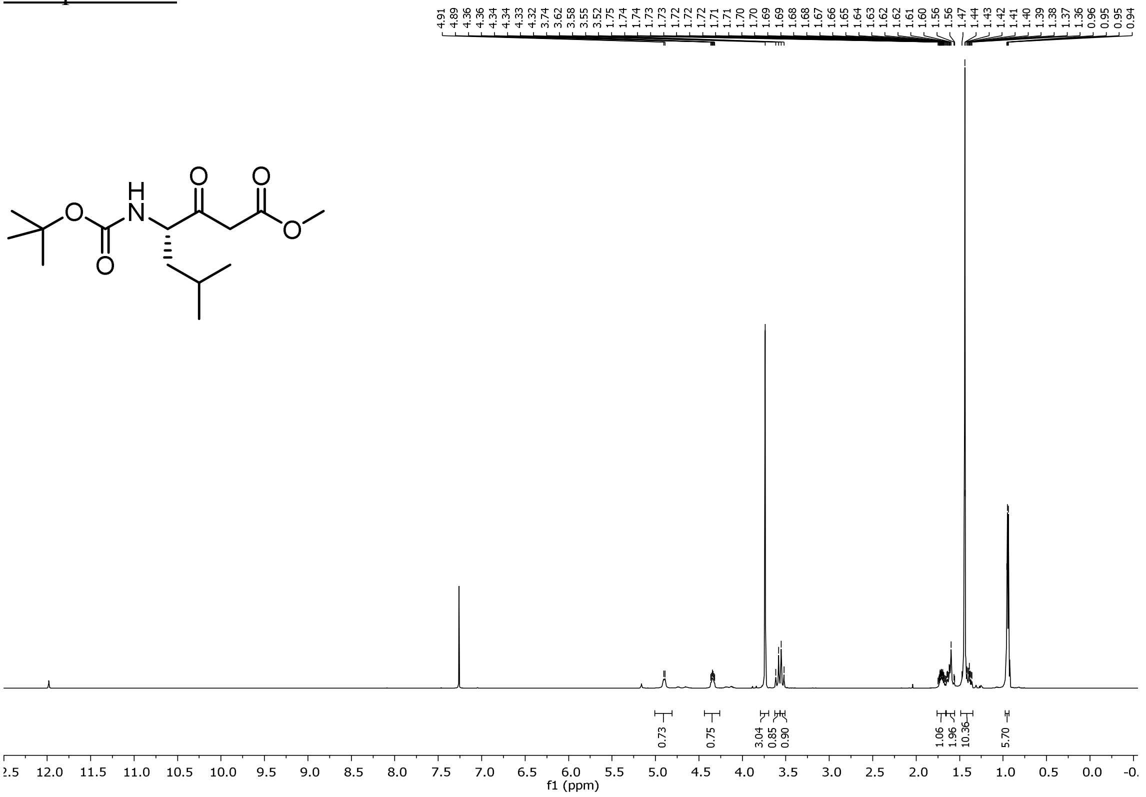

Compound **15**

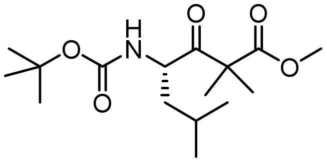
H
N
O
O
O
O
O

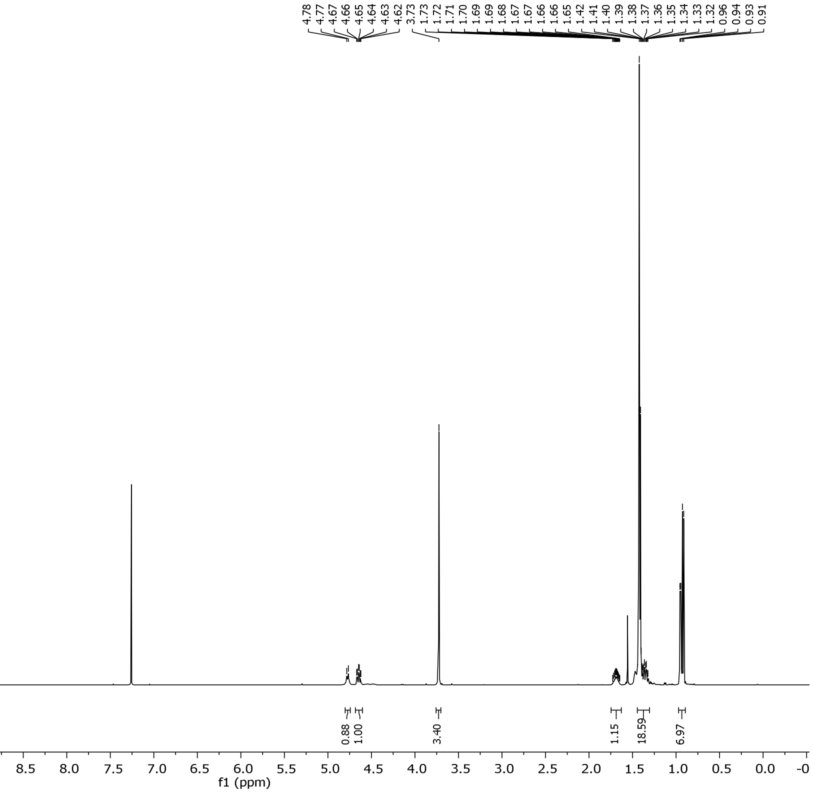
4.78 4.77 4.67 4.66 4.65 4.64 4.63 4.62 3.73 1.73 1.72 1.71 1.70 1.69 1.69 1.68 1.67 1.67 1.66 1.66 1.65 1.42 1.41 1.40 1.39 1.38 1.37 1.36 1.35 1.34 1.33 1.32 0.96 0.94 0.93 0.91
0.88
1.00
3.40
1.15
18.59
6.97
2.5 12.0 11.5 11.0 10.5 10.0 9.5 9.0 8.5 8.0 7.5 7.0 6.5 6.0 5.5 5.0 4.5 4.0 3.5 3.0 2.5 2.0 1.5 1.0 0.5 0.0 -0
f1 (ppm)

Compound **10**

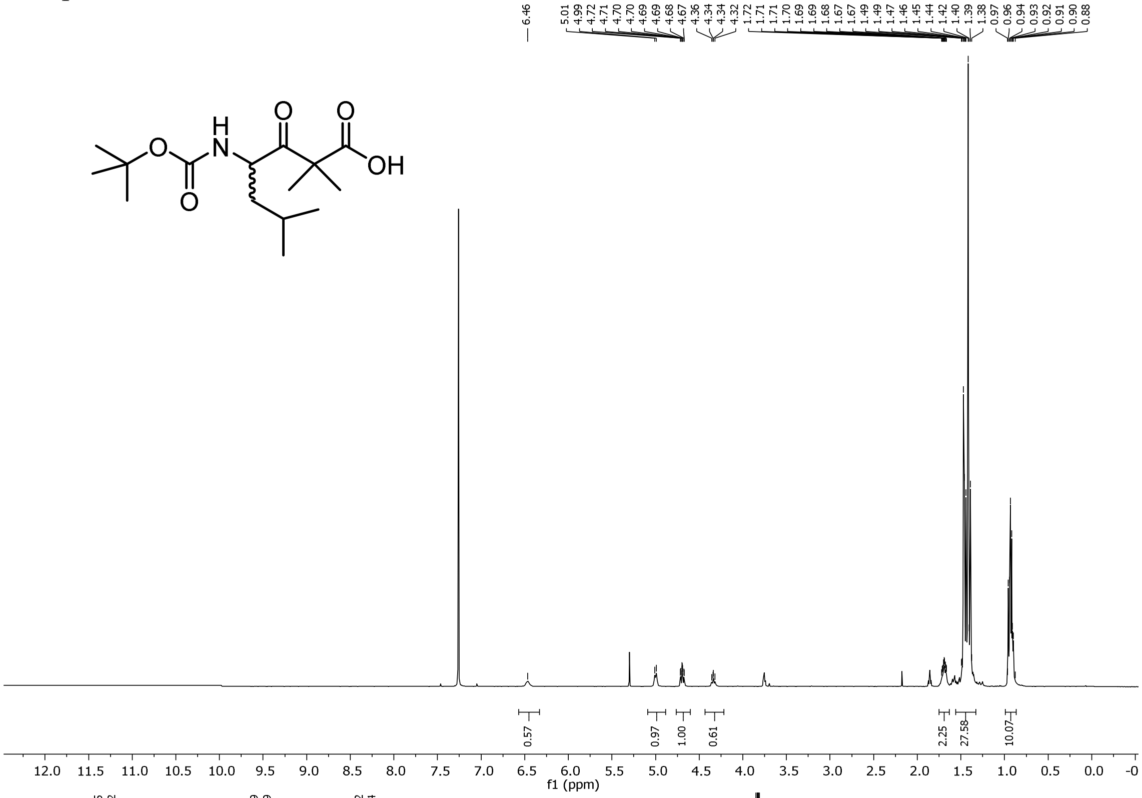

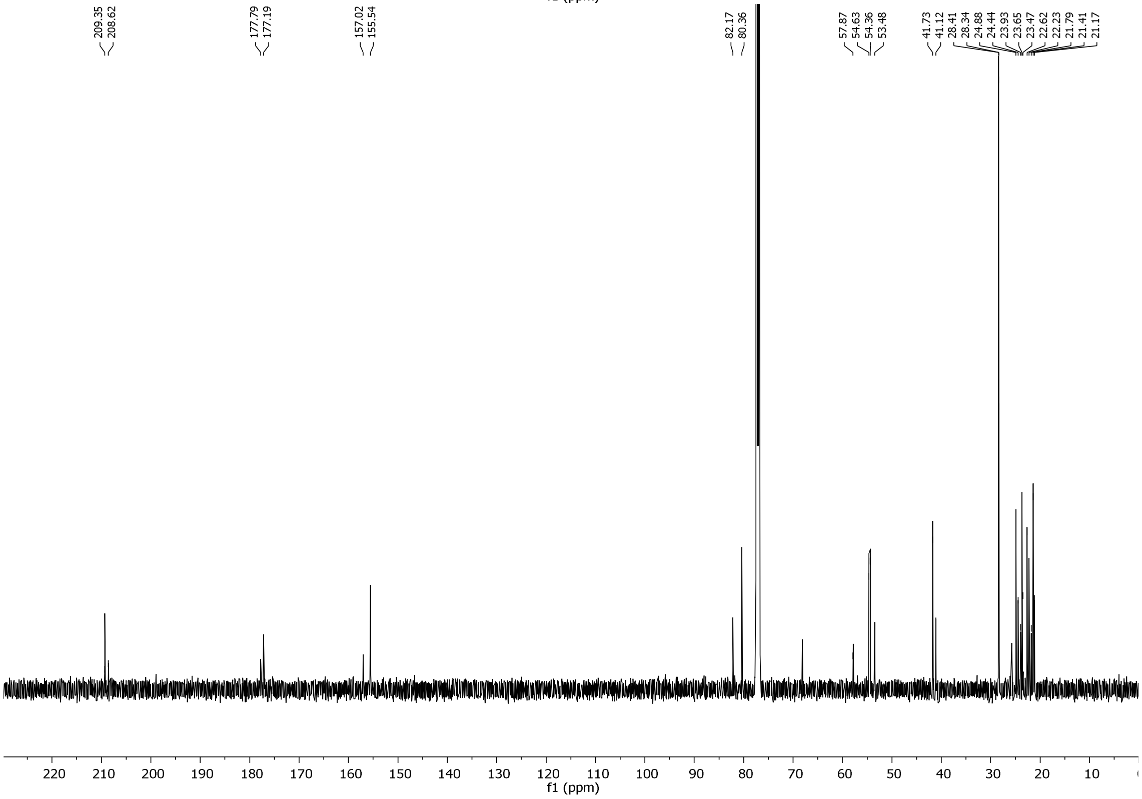

Compound **11**

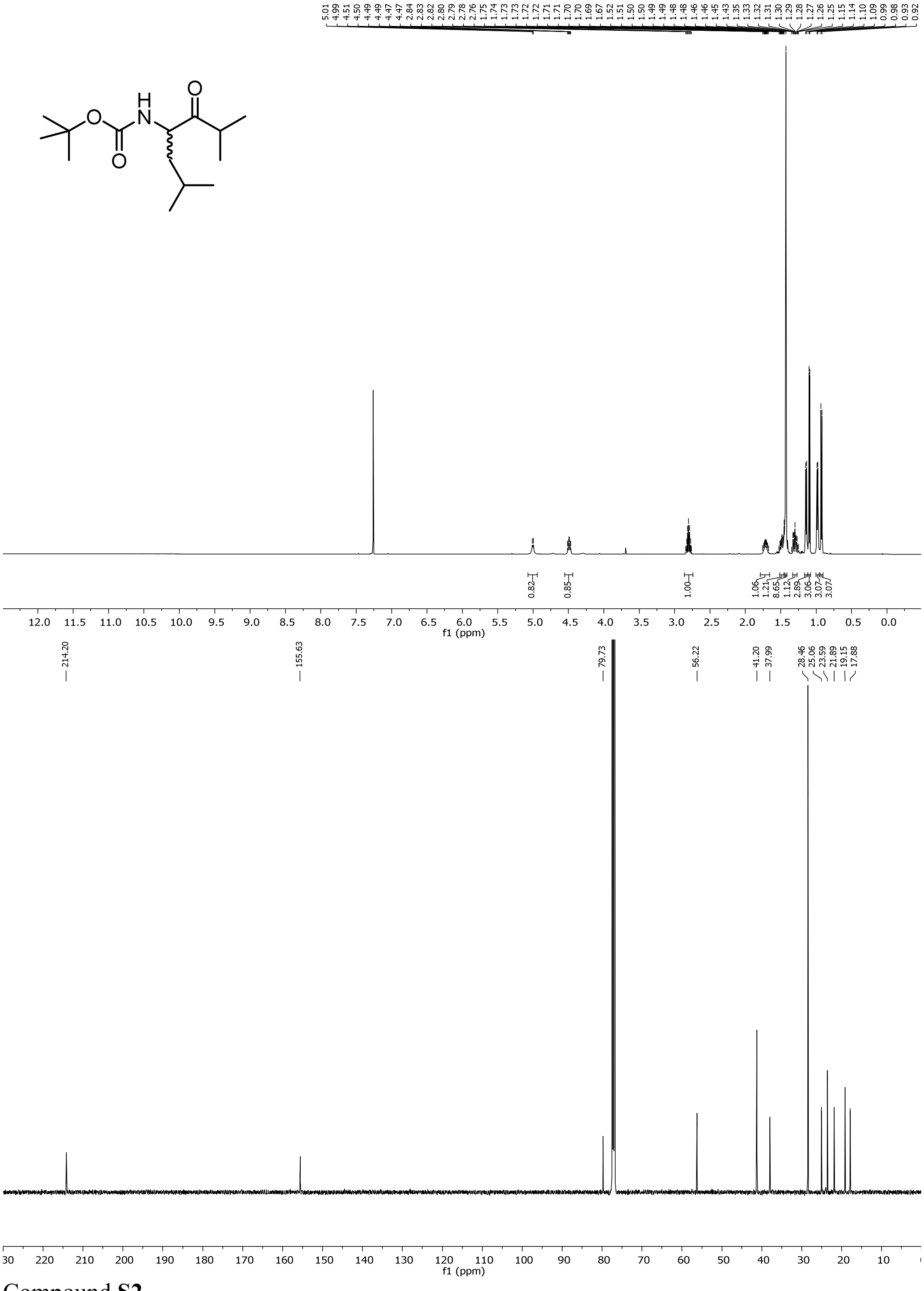
5.01 4.99 4.51 4.50 4.49 4.49 4.47 2.84 2.83 2.82 2.80 2.79 2.78 2.76 1.75 1.74 1.73 1.73 1.72 1.72 1.71 1.71 1.70 1.69 1.67 1.52 1.51 1.50 1.50 1.49 1.49 1.48 1.48 1.46 1.46 1.45 1.43 1.35 1.33 1.32 1.31 1.30 1.29 1.28 1.27 1.26 1.25 1.15 1.14 1.10 1.09 0.99 0.98 0.93 0.92
O
H
N
O
O
0.82
0.85
1.00
1.06
1.21
8.65
1.12
2.89
3.06
3.07
3.07
f1 (ppm)
214.20
155.63
79.73
56.22
41.20
37.99
28.46
25.06
23.59
21.89
19.15
17.88
f1 (ppm)

Compound **S2**

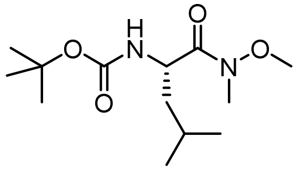

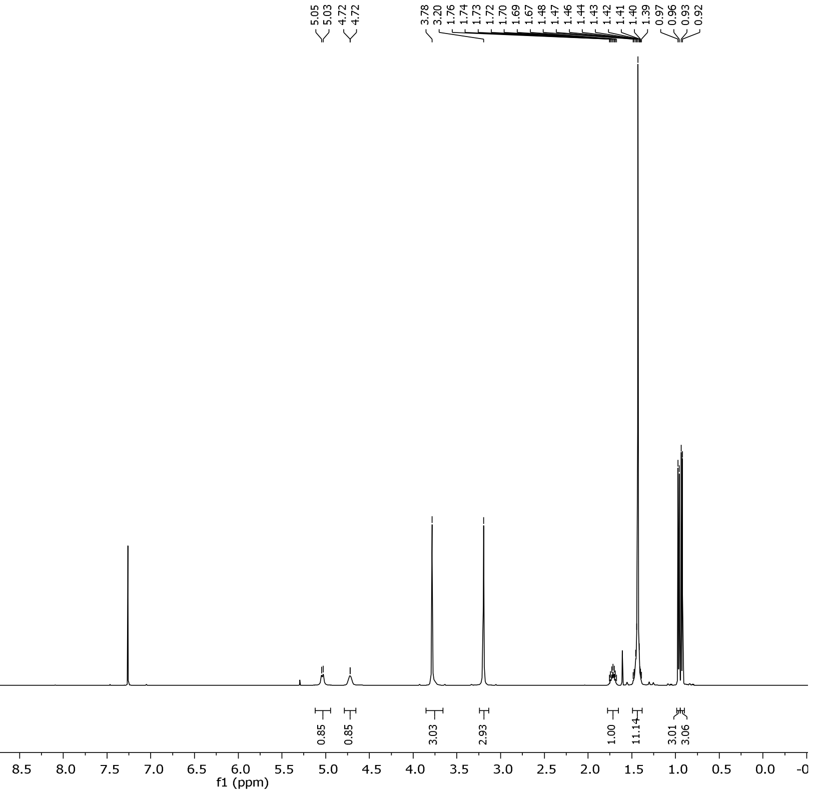
5.05
5.03
4.72
4.72
3.78
3.20
1.76
1.74
1.73
1.72
1.70
1.69
1.67
1.48
1.47
1.46
1.44
1.43
1.42
1.41
1.40
1.39
0.97
0.96
0.93
0.92
0.85
0.85
3.03
2.93
1.00
11.14
3.01
3.06
12.0 11.5 11.0 10.5 10.0 9.5 9.0 8.5 8.0 7.5 7.0 6.5 6.0 5.5 5.0 4.5 4.0 3.5 3.0 2.5 2.0 1.5 1.0 0.5 0.0
f1 (ppm)

Compound **14**

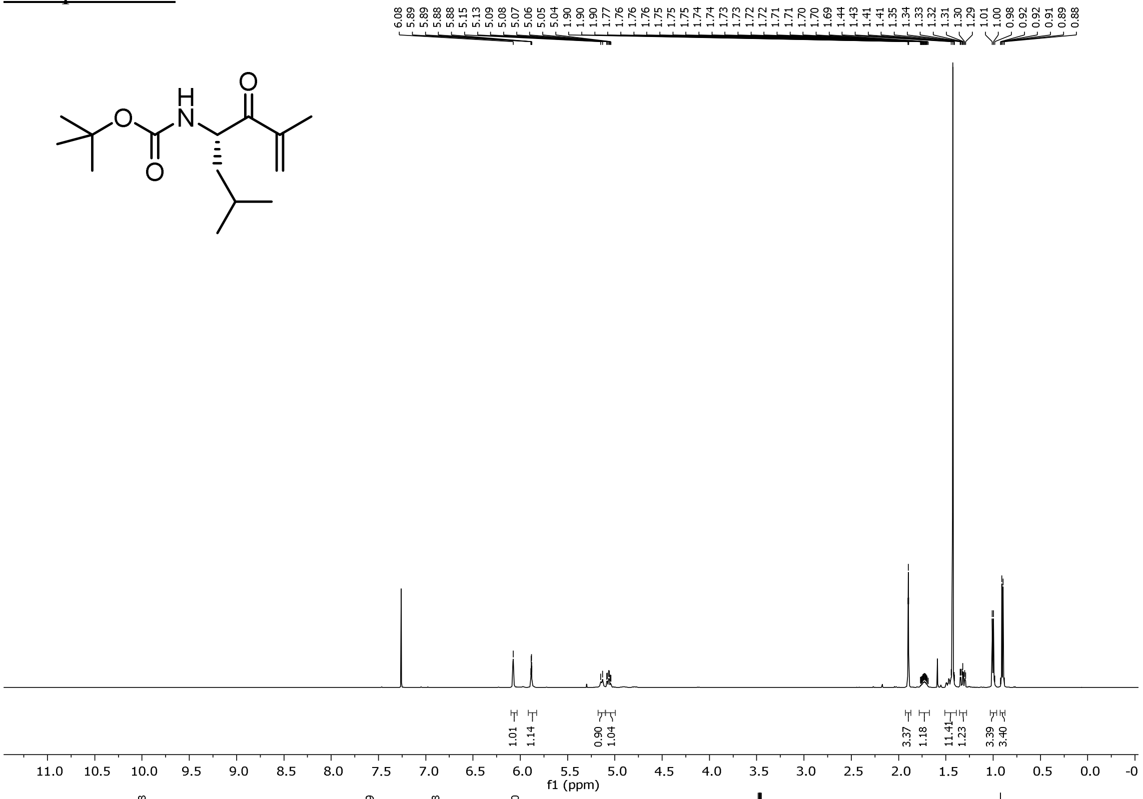

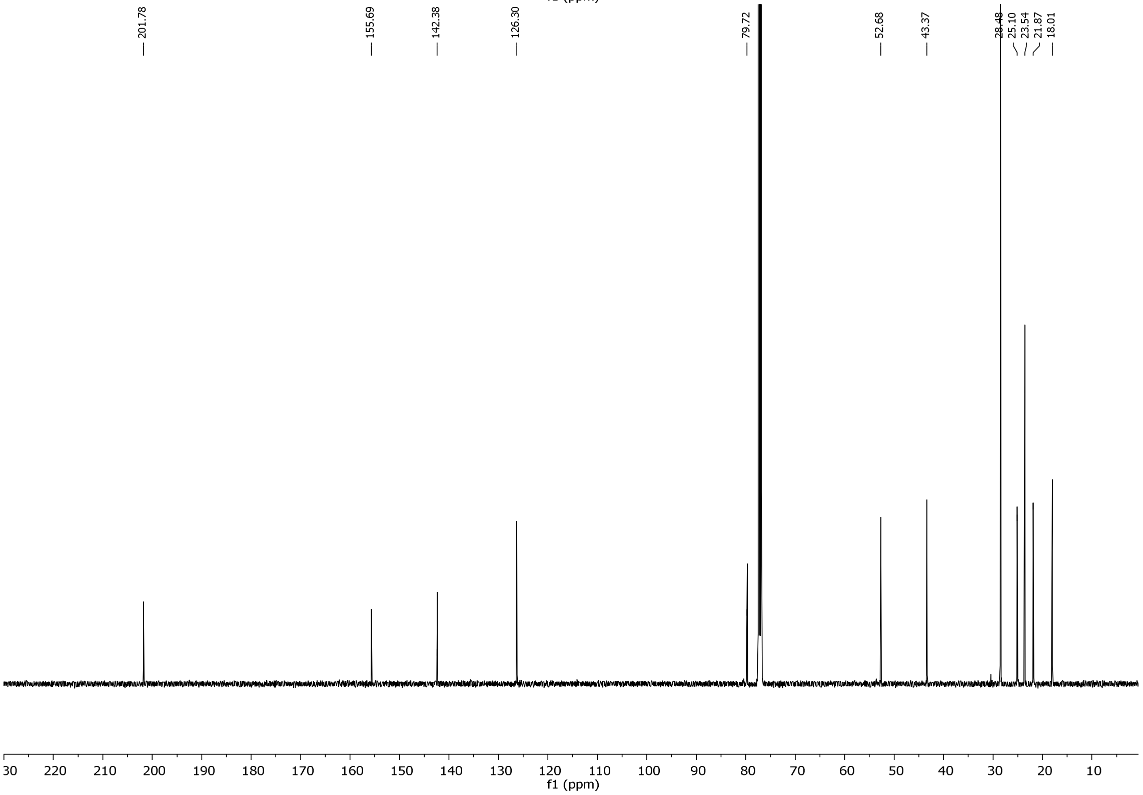

Compound **16**

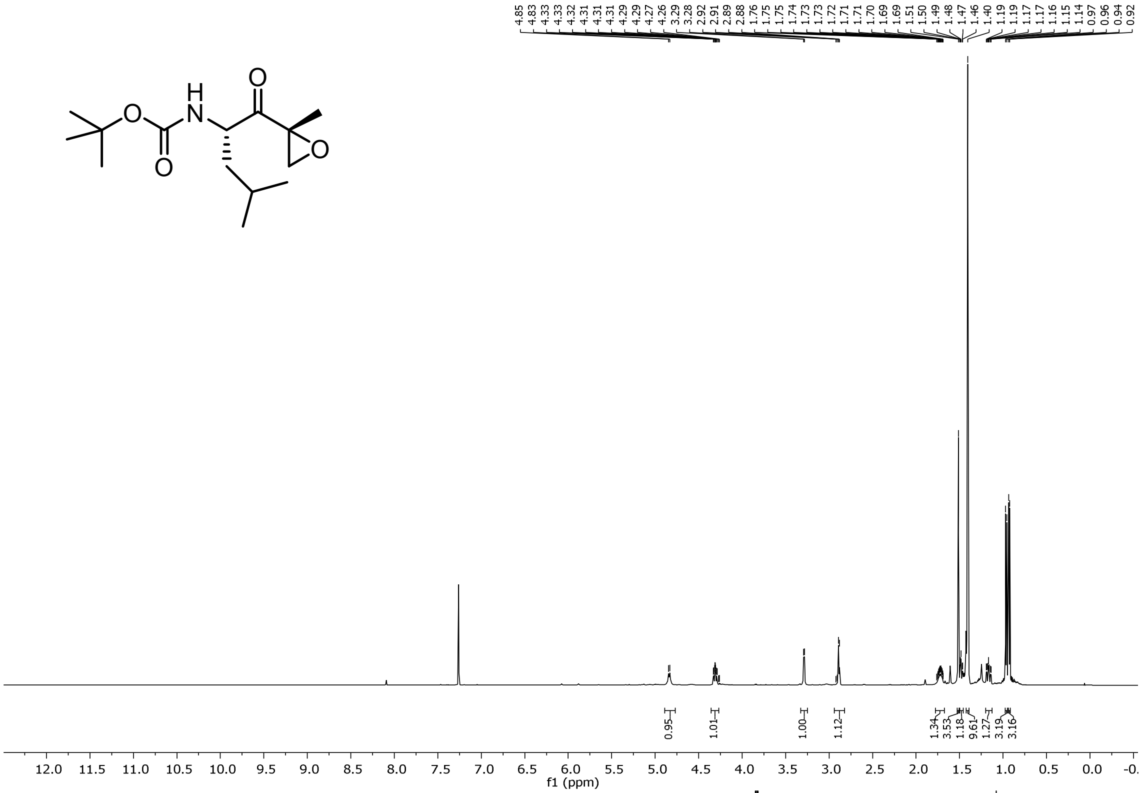

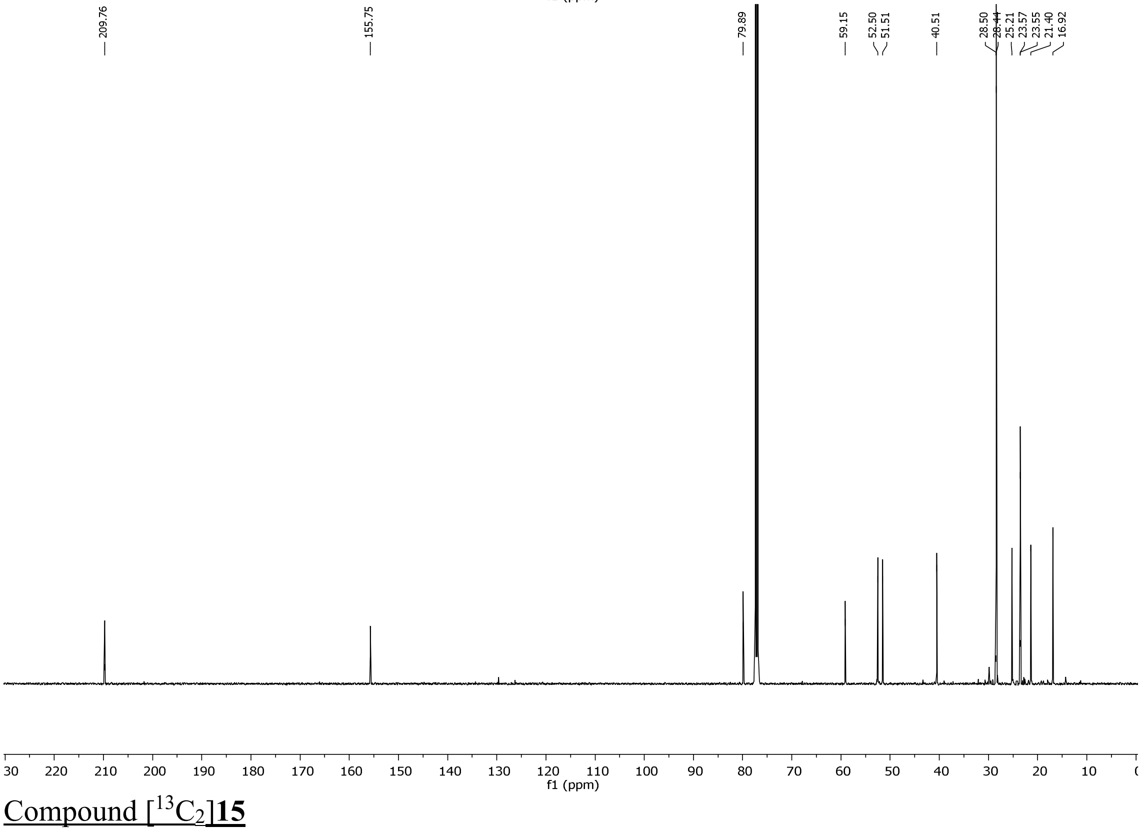

Compound [$^{13}C_2$]**15**

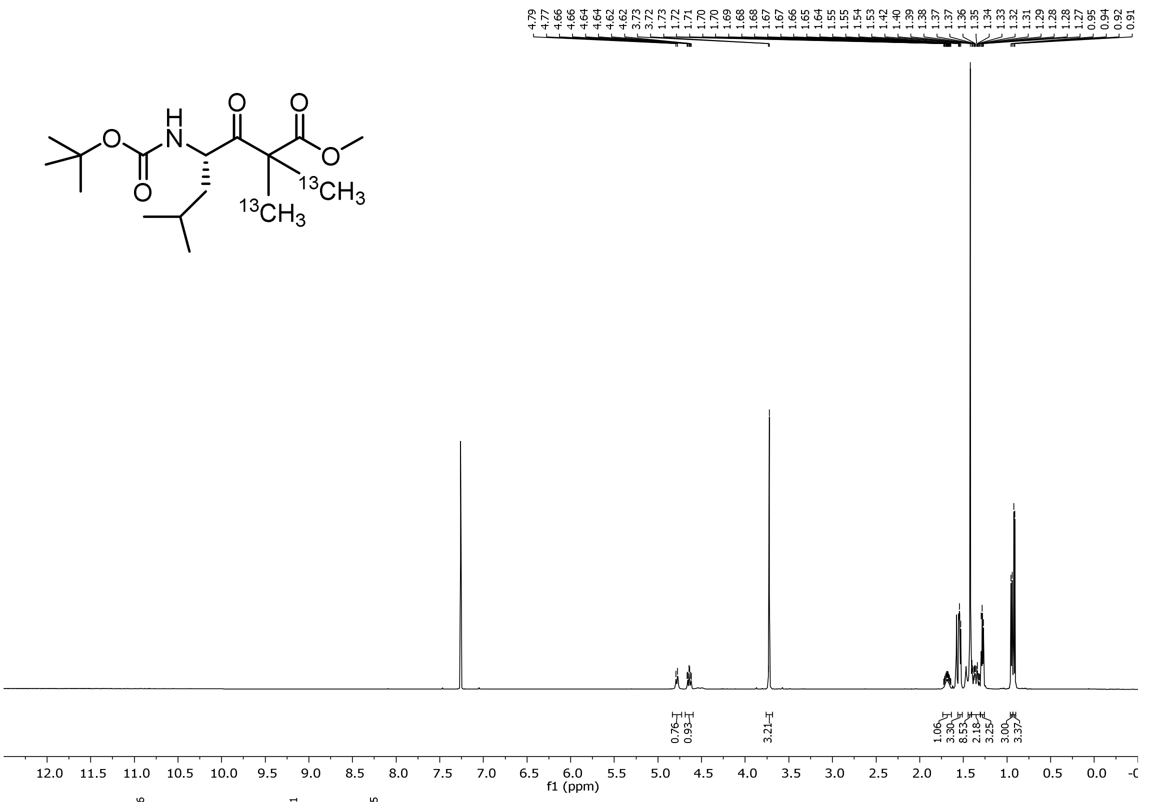
f1 (ppm)

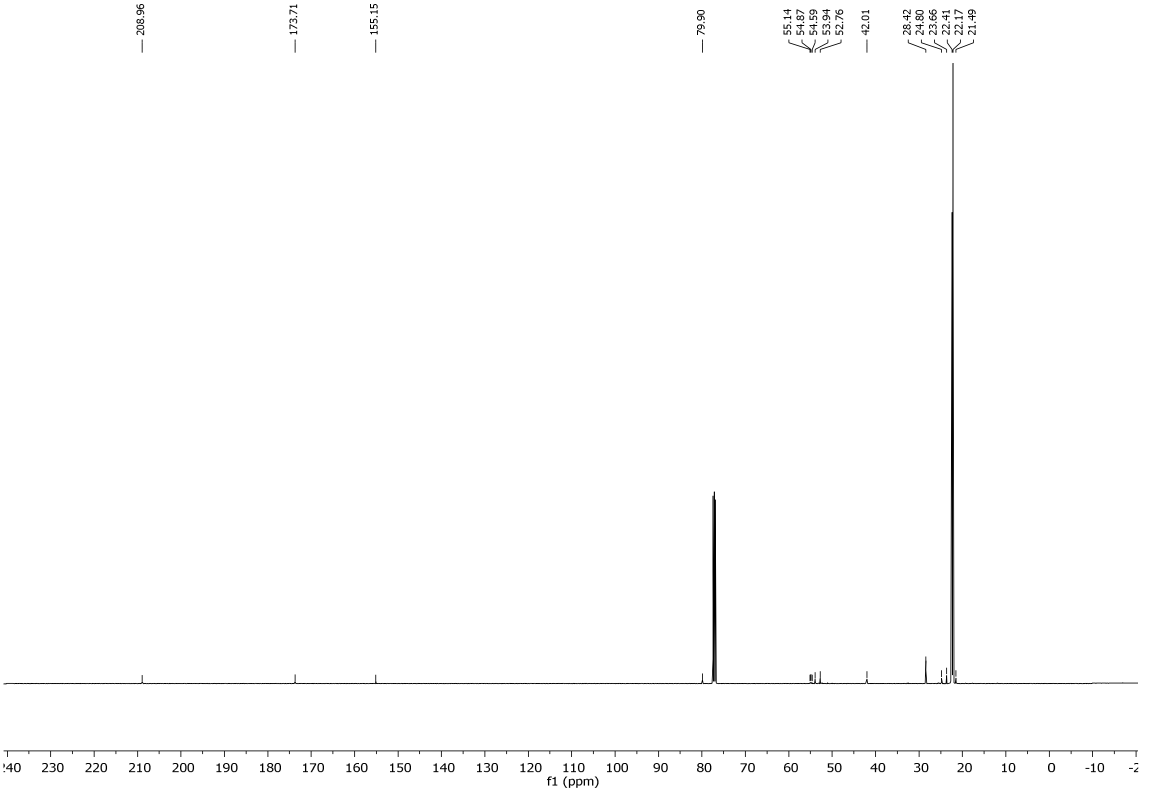
208.96
173.71
155.15
79.90
55.14
54.87
54.59
53.94
52.76
42.01
28.42
24.80
23.66
22.41
22.17
21.49
f1 (ppm)

Compound **S3**

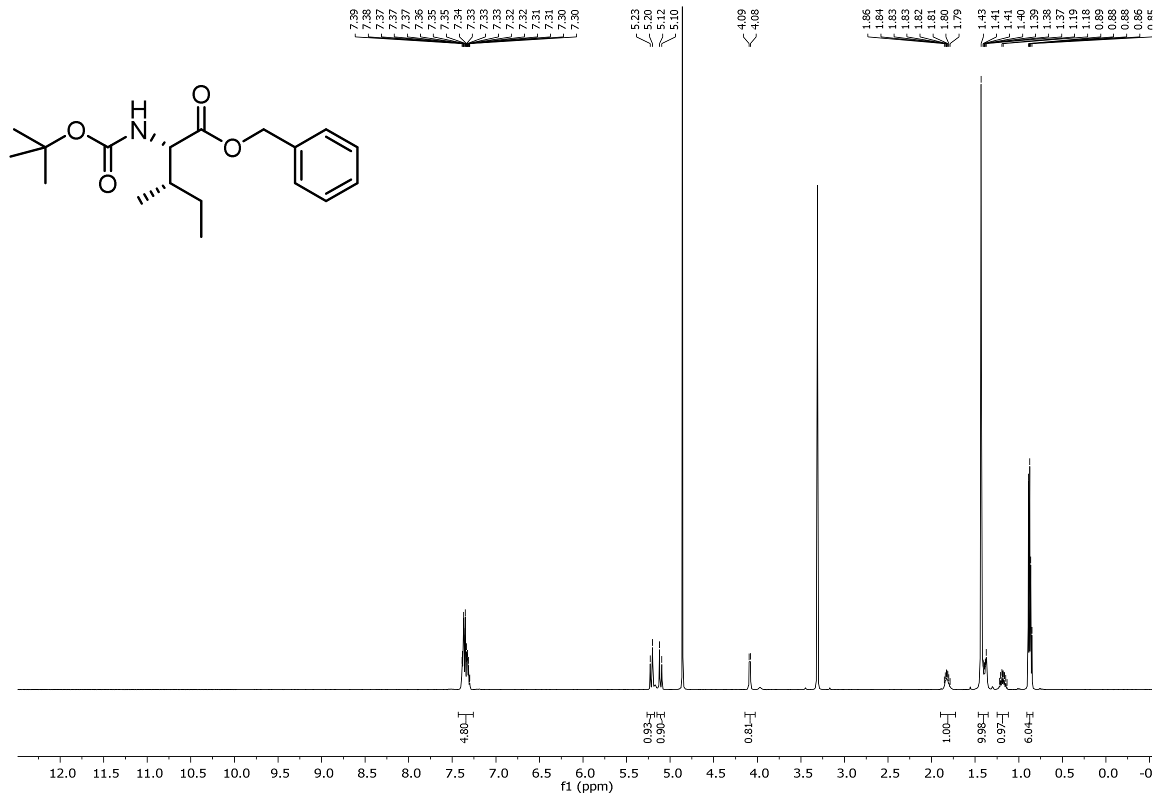

Compound **S4**

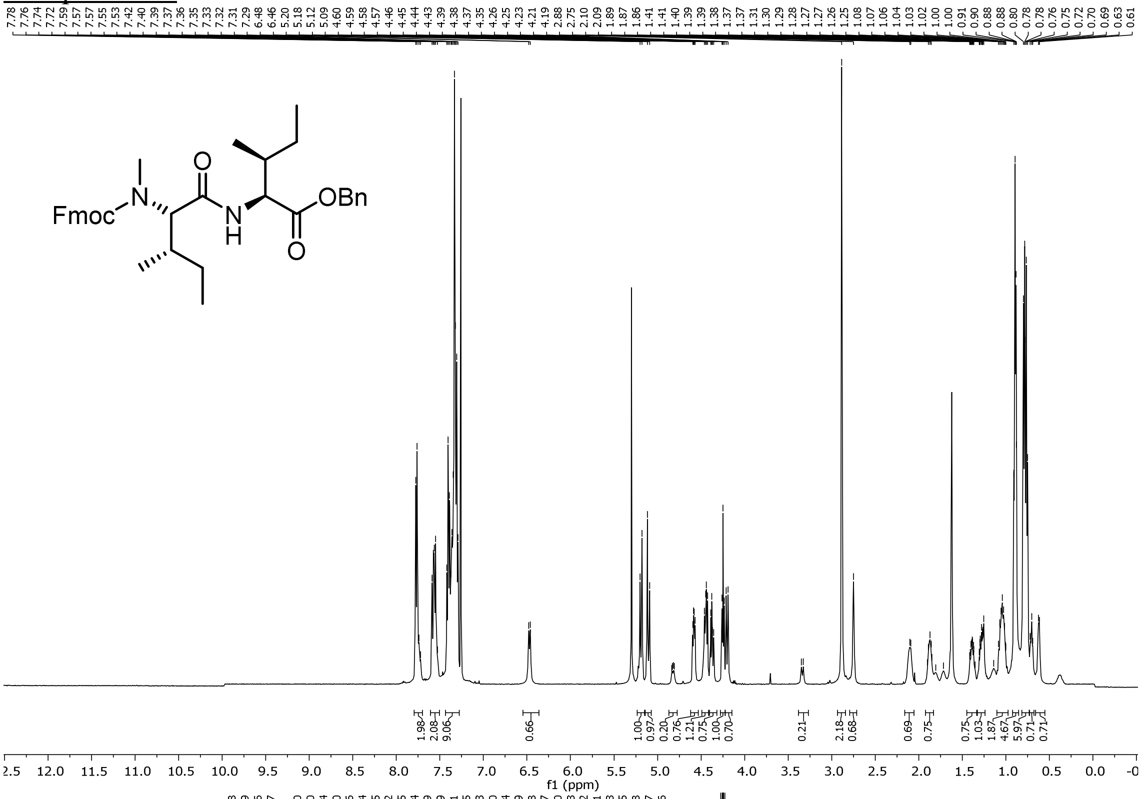

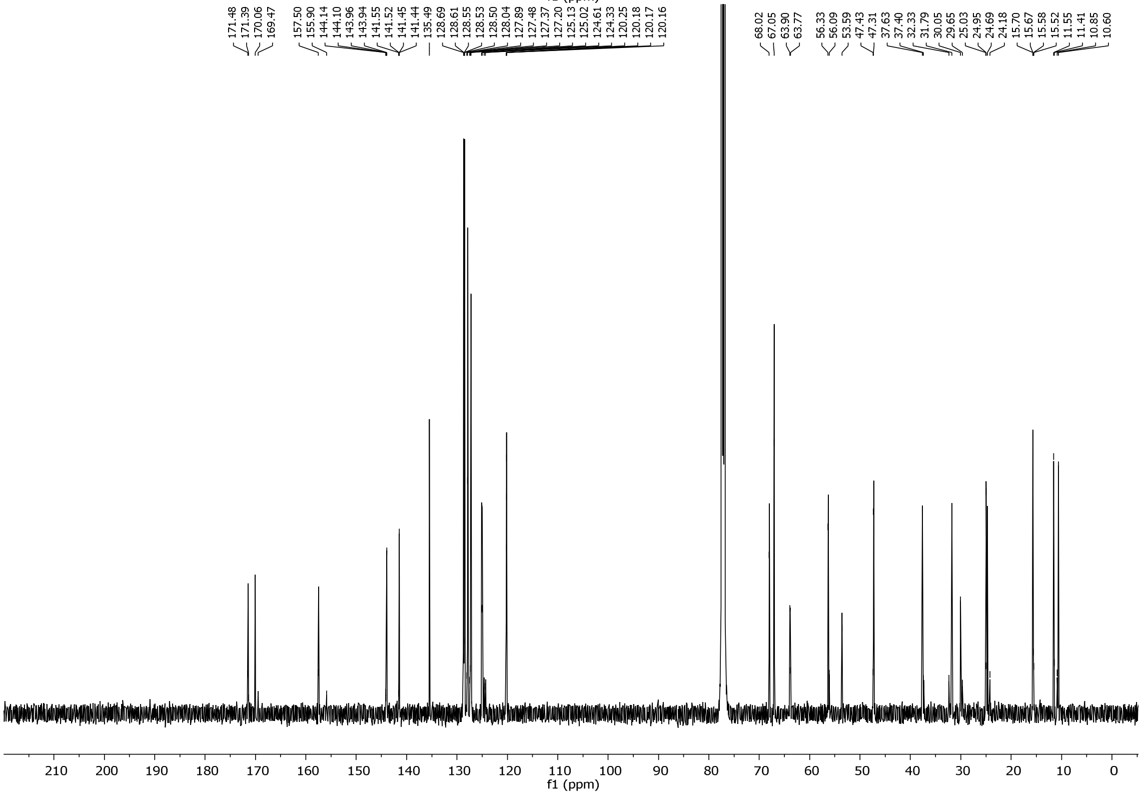

Compound **S5**

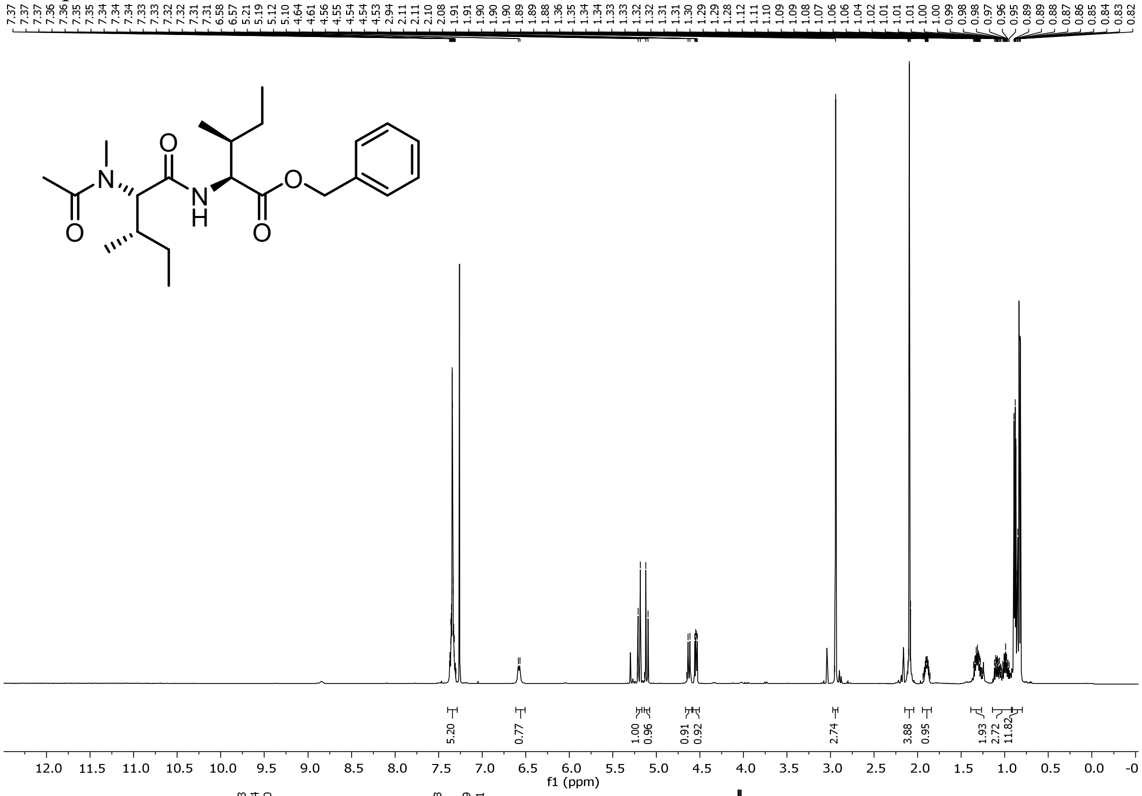
7.37 7.37 7.37 7.36 7.36 7.35 7.35 7.34 7.34 7.34 7.33 7.33 7.32 7.32 7.31 7.31 6.58 6.57 5.21 5.19 5.12 5.10 4.64 4.61 4.56 4.55 4.54 4.54 4.53 2.94 2.11 2.11 2.10 2.08 1.91 1.91 1.90 1.90 1.90 1.89 1.89 1.88 1.36 1.35 1.34 1.34 1.33 1.33 1.32 1.32 1.31 1.31 1.30 1.29 1.29 1.28 1.12 1.11 1.10 1.09 1.09 1.08 1.07 1.06 1.06 1.04 1.02 1.01 1.01 1.01 1.00 1.00 0.99 0.98 0.98 0.97 0.96 0.95 0.89 0.89 0.88 0.87 0.86 0.85 0.84 0.83 0.82
O
N
N
H
O
O
O
5.20
0.77
1.00
0.96
0.91
0.92
2.74
3.88
0.95
1.93
2.72
11.82
12.0 11.5 11.0 10.5 10.0 9.5 9.0 8.5 8.0 7.5 7.0 6.5 6.0 5.5 5.0 4.5 4.0 3.5 3.0 2.5 2.0 1.5 1.0 0.5 0.0
f1 (ppm)

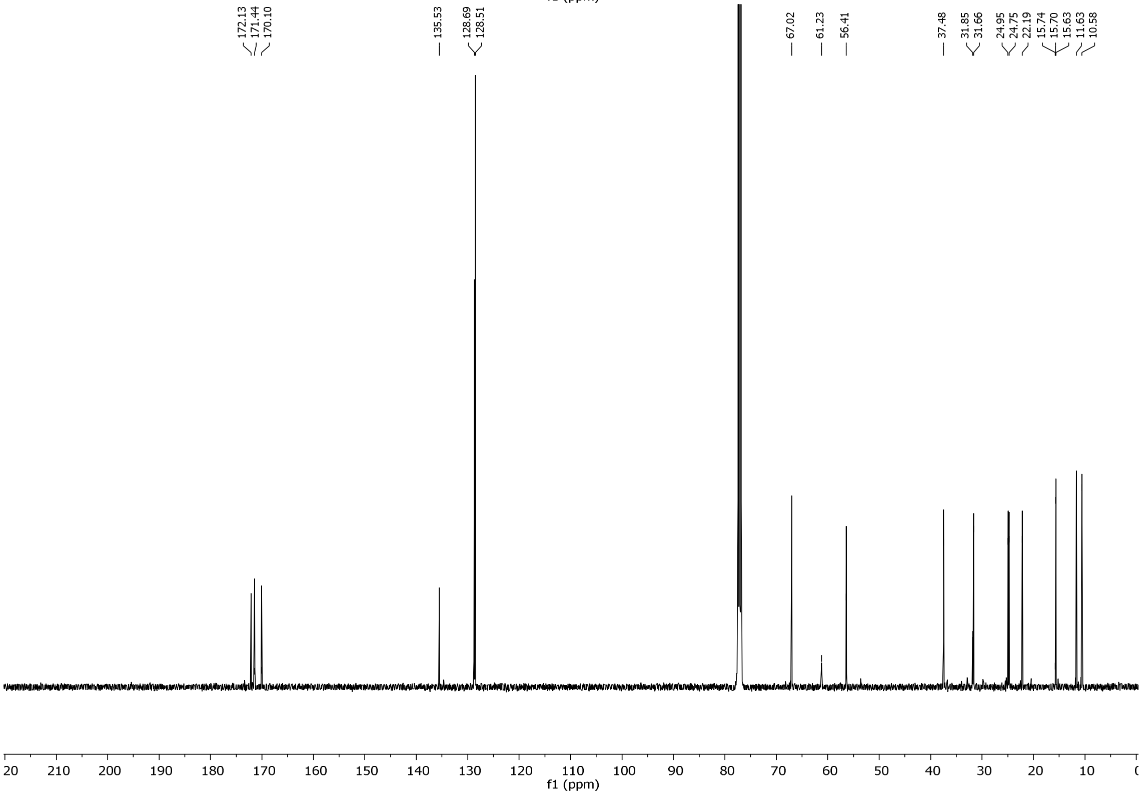
172.13
171.44
170.10
135.53
128.69
128.51
67.02
61.23
56.41
37.48
31.85
31.66
24.95
24.75
22.19
15.74
15.70
15.63
11.63
10.58
210 200 190 180 170 160 150 140 130 120 110 100 90 80 70 60 50 40 30 20 10
f1 (ppm)

Compound **S6**

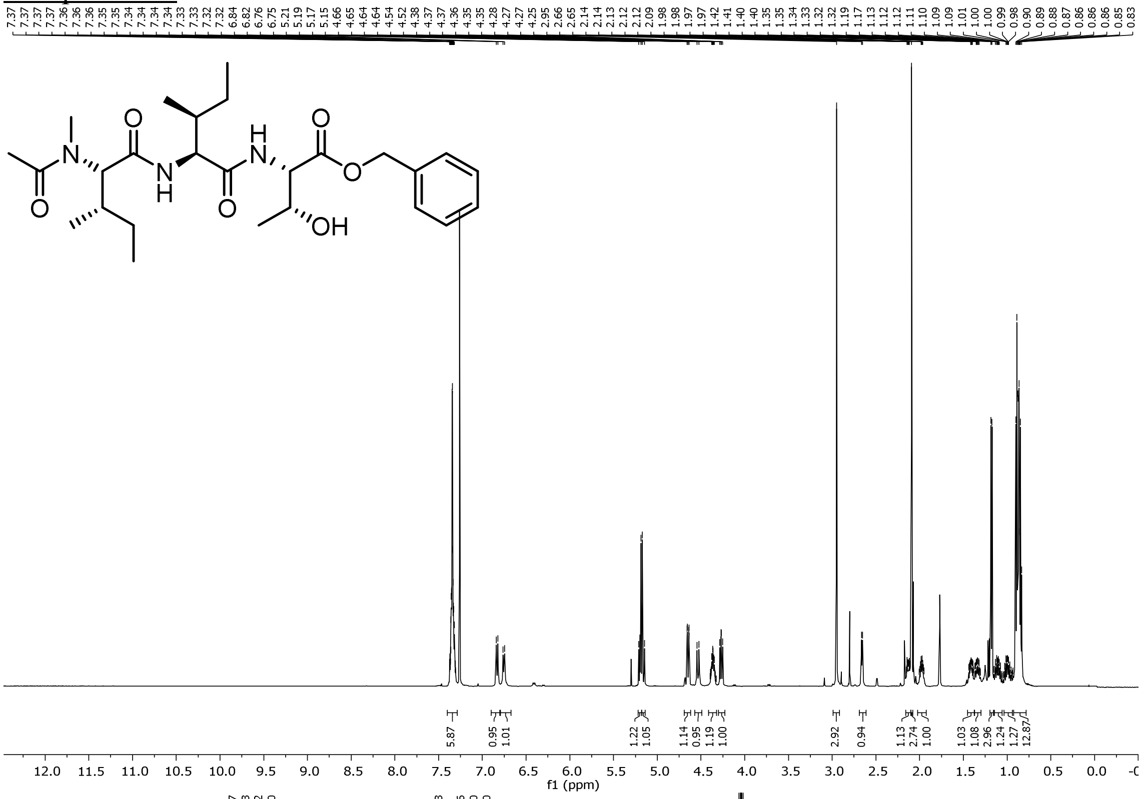

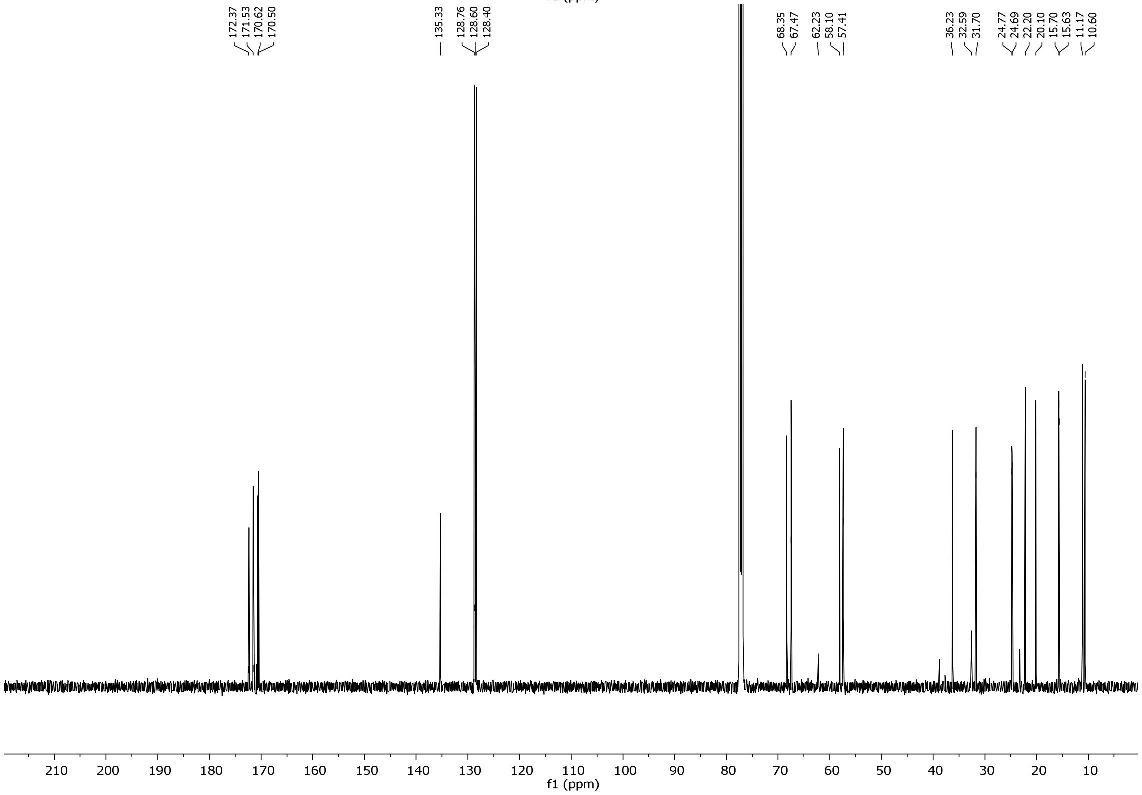

Compound **17**

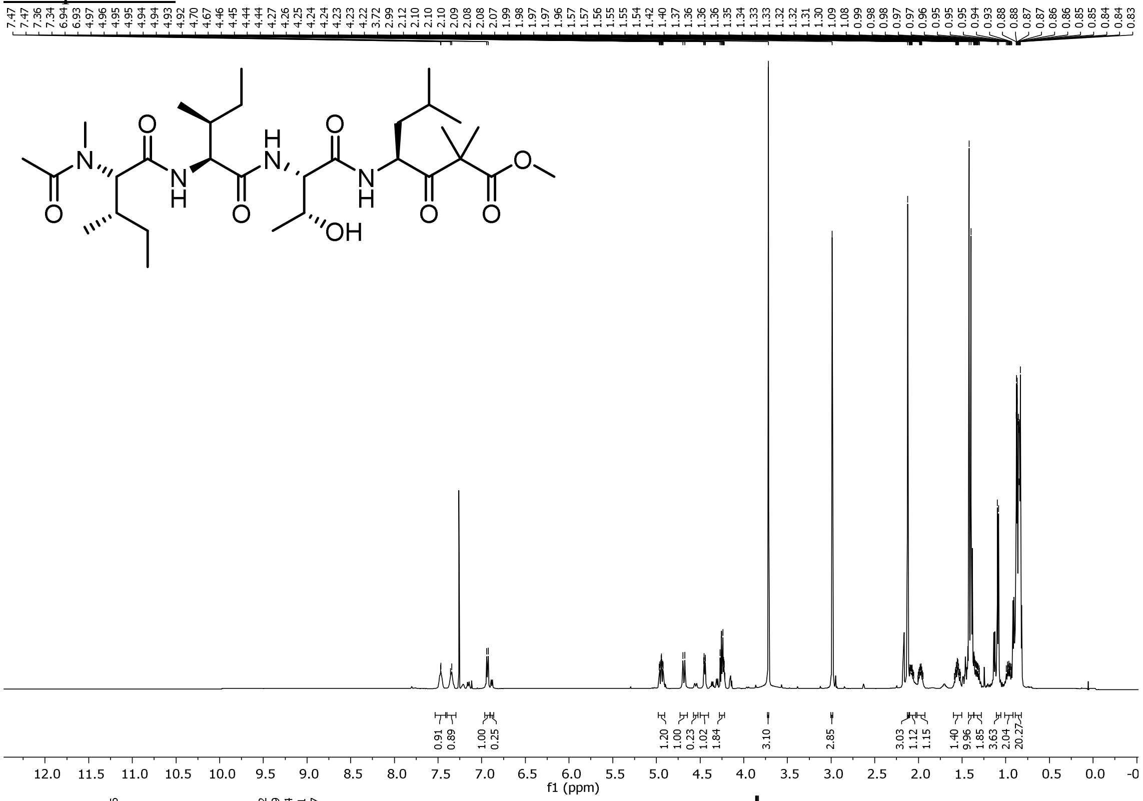
7.47 7.47 7.36 7.34 6.94 6.93 4.97 4.96 4.95 4.95 4.94 4.94 4.93 4.92 4.70 4.67 4.46 4.45 4.44 4.44 4.27 4.26 4.25 4.24 4.24 4.23 4.23 4.22 3.72 2.99 2.12 2.10 2.10 2.09 2.08 2.08 2.07 1.99 1.98 1.97 1.97 1.96 1.57 1.57 1.56 1.55 1.55 1.54 1.42 1.40 1.37 1.36 1.36 1.35 1.34 1.33 1.33 1.32 1.32 1.31 1.30 1.09 1.08 0.99 0.98 0.98 0.97 0.97 0.96 0.95 0.95 0.95 0.94 0.93 0.88 0.88 0.87 0.87 0.86 0.86 0.85 0.85 0.84 0.83
O
N
H
OH
0.91 0.89 1.00 0.25 1.20 1.00 0.23 1.02 1.84 3.10 2.85 3.03 1.12 1.15 1.40 9.96 1.85 3.63 2.04 20.27
12.0 11.5 11.0 10.5 10.0 9.5 9.0 8.5 8.0 7.5 7.0 6.5 6.0 5.5 5.0 4.5 4.0 3.5 3.0 2.5 2.0 1.5 1.0 0.5 0.0 -0
f1 (ppm)

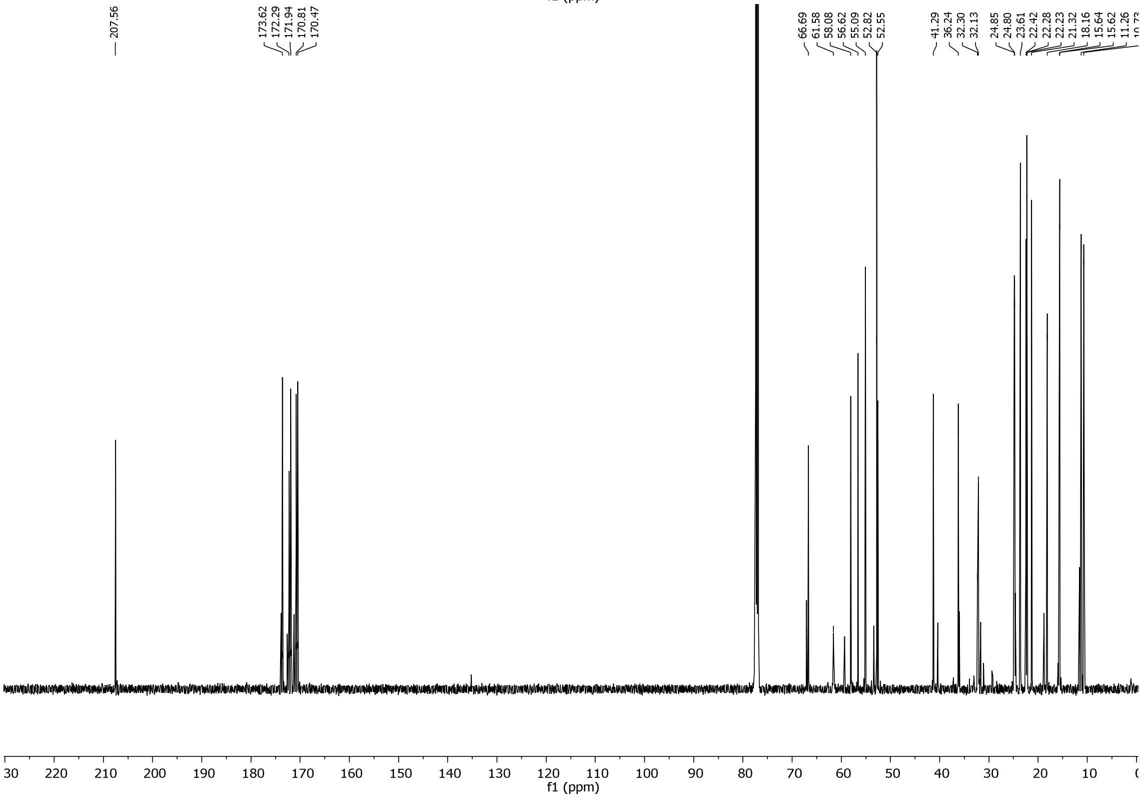
207.56
173.62 172.29 171.94 170.81 170.47
66.69 61.58 58.08 56.62 55.09 52.82 52.55
41.29 36.24 32.30 32.13 24.85 24.80 23.61 22.42 22.28 22.23 21.32 18.16 15.64 15.62 11.26
30 220 210 200 190 180 170 160 150 140 130 120 110 100 90 80 70 60 50 40 30 20 10
f1 (ppm)

Compound [$^{13}C_2$]**17**

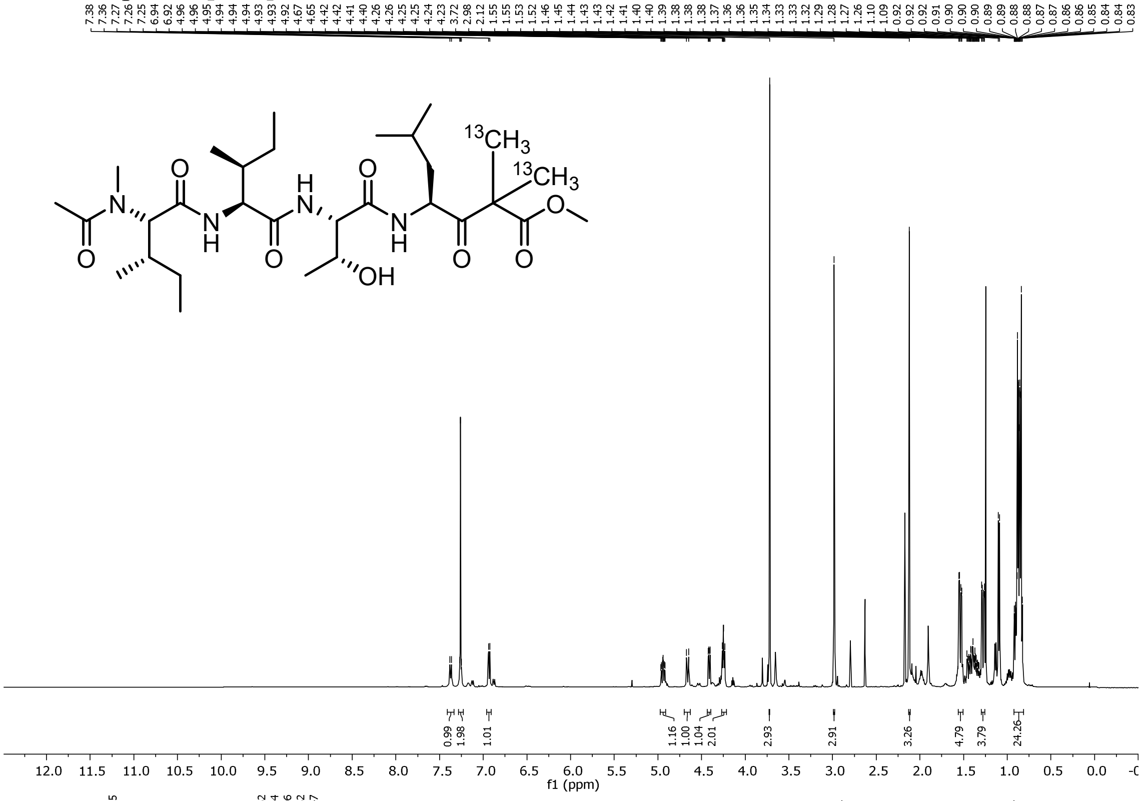

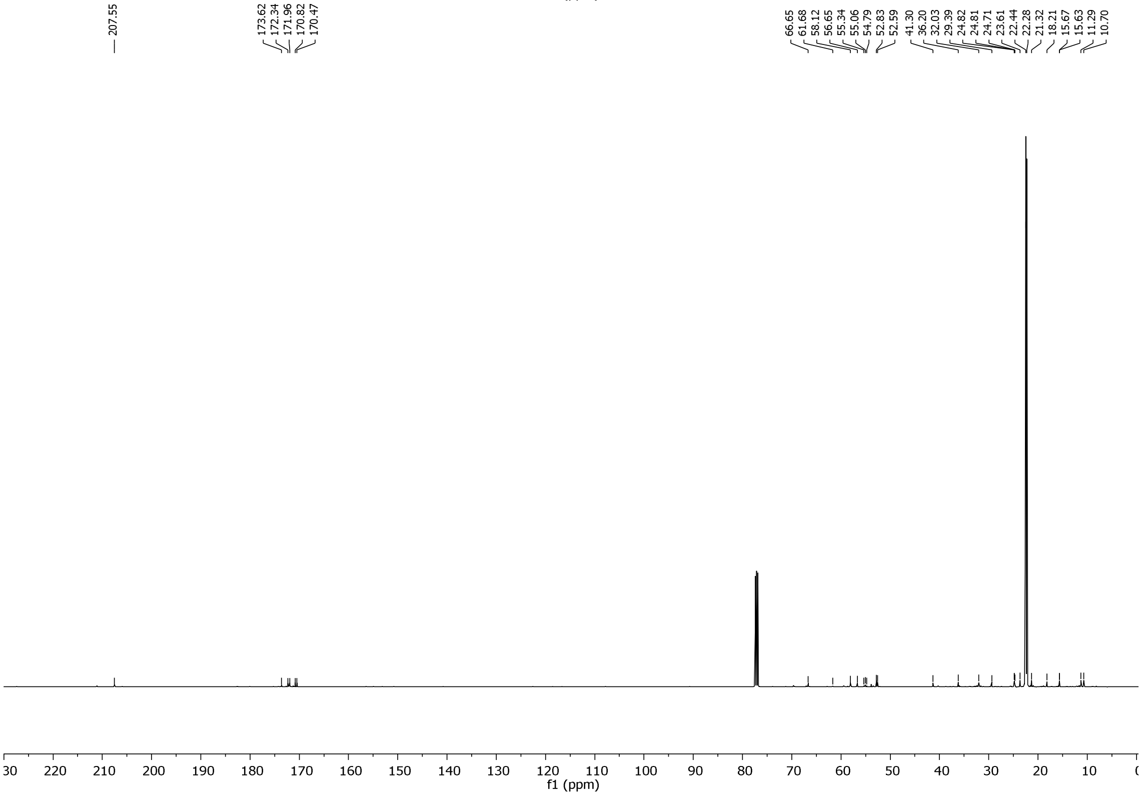

Compound **12**

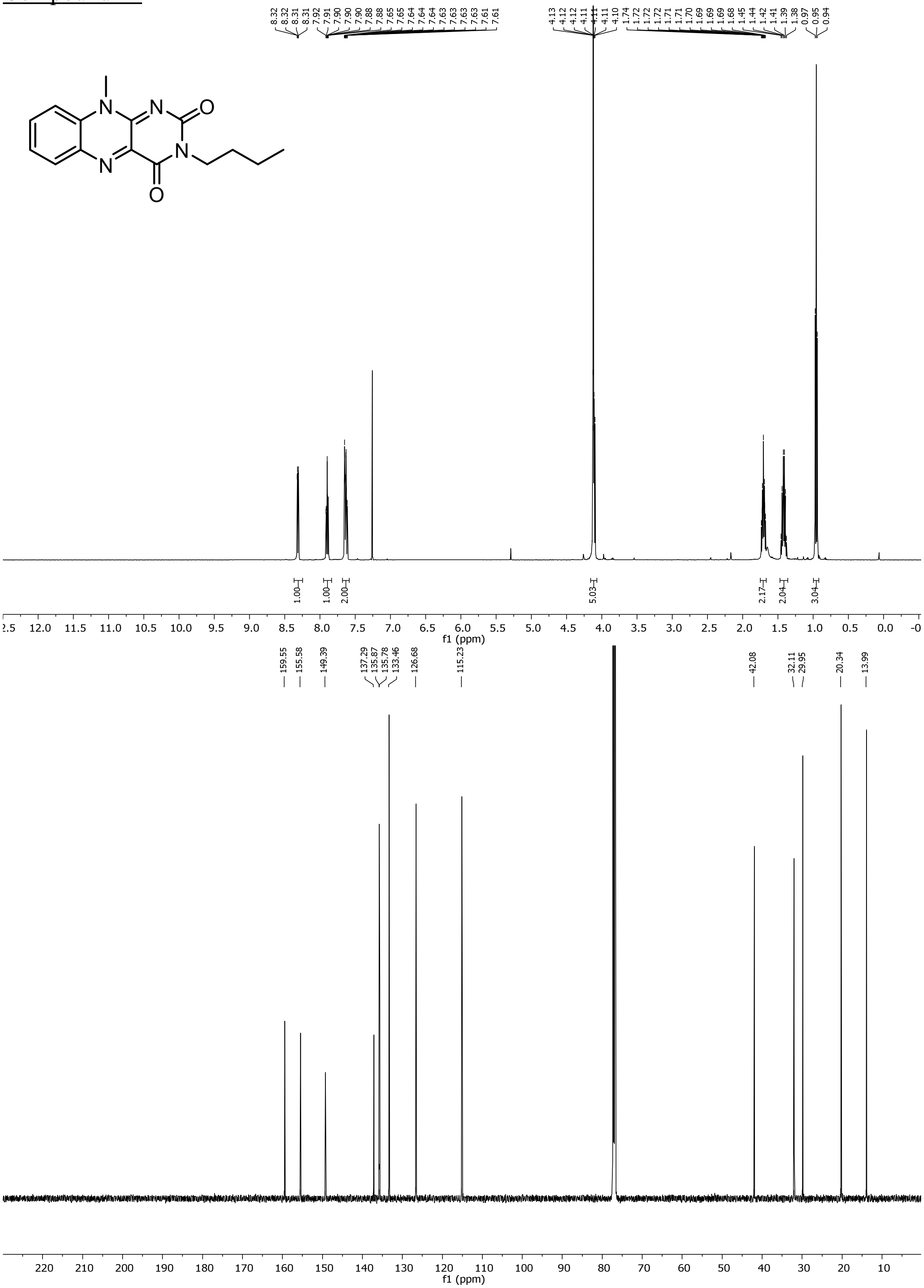

Manuscript Figure 4C – Panel #1

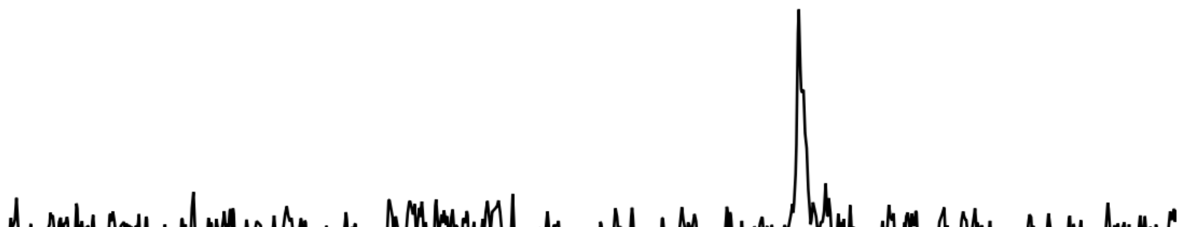

Manuscript Figure 4C – Panel #2

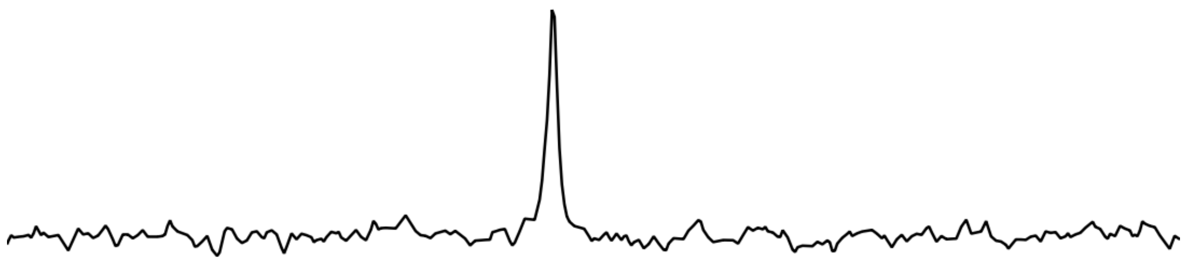

Manuscript Figure 4C – Panel #3

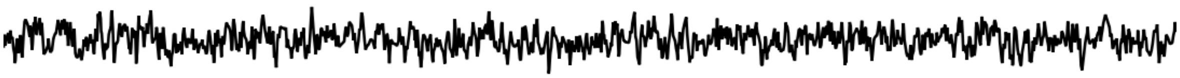

Manuscript Figure 4D – Panel #1

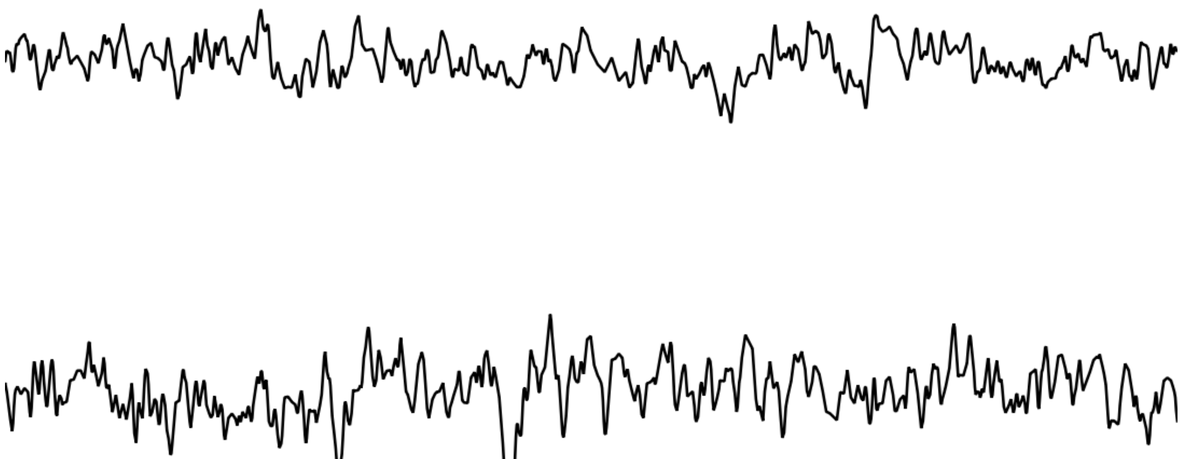

Manuscript Figure 4D – Panel #2

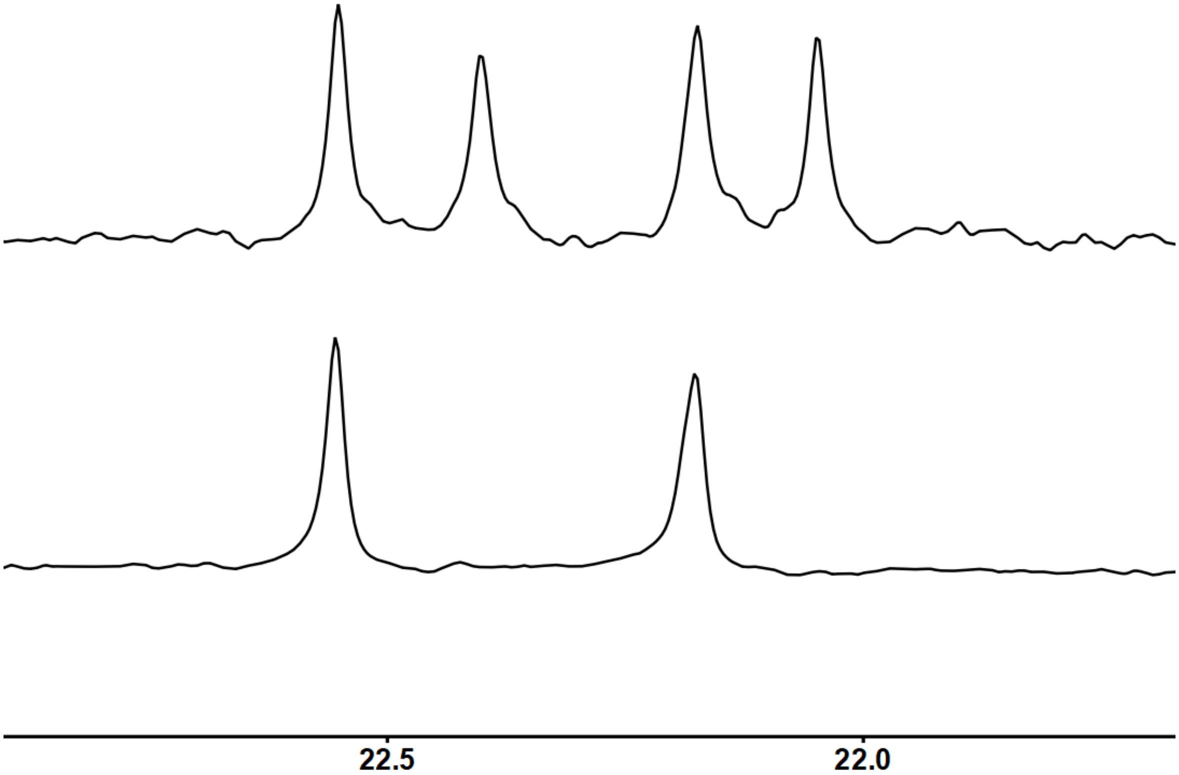

Manuscript Figure 4D – Panel #3

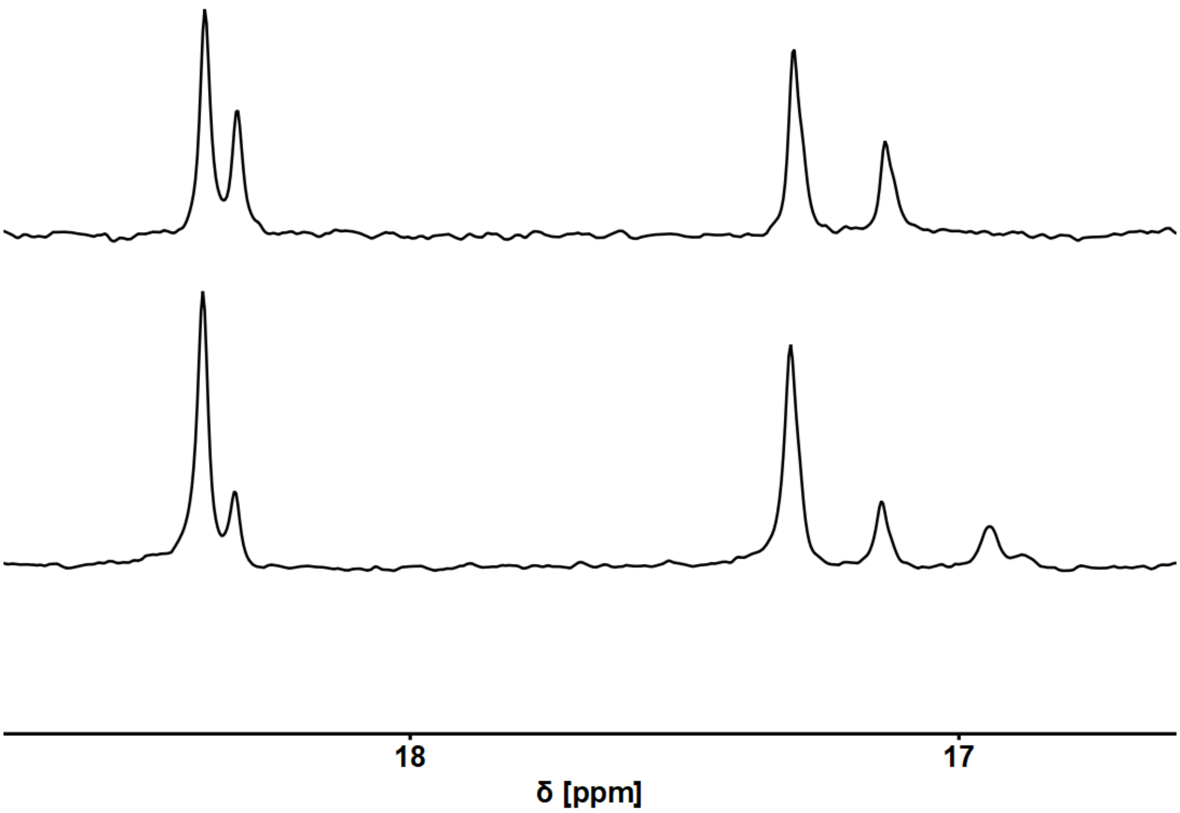

Manuscript Figure 4E – Panel #1

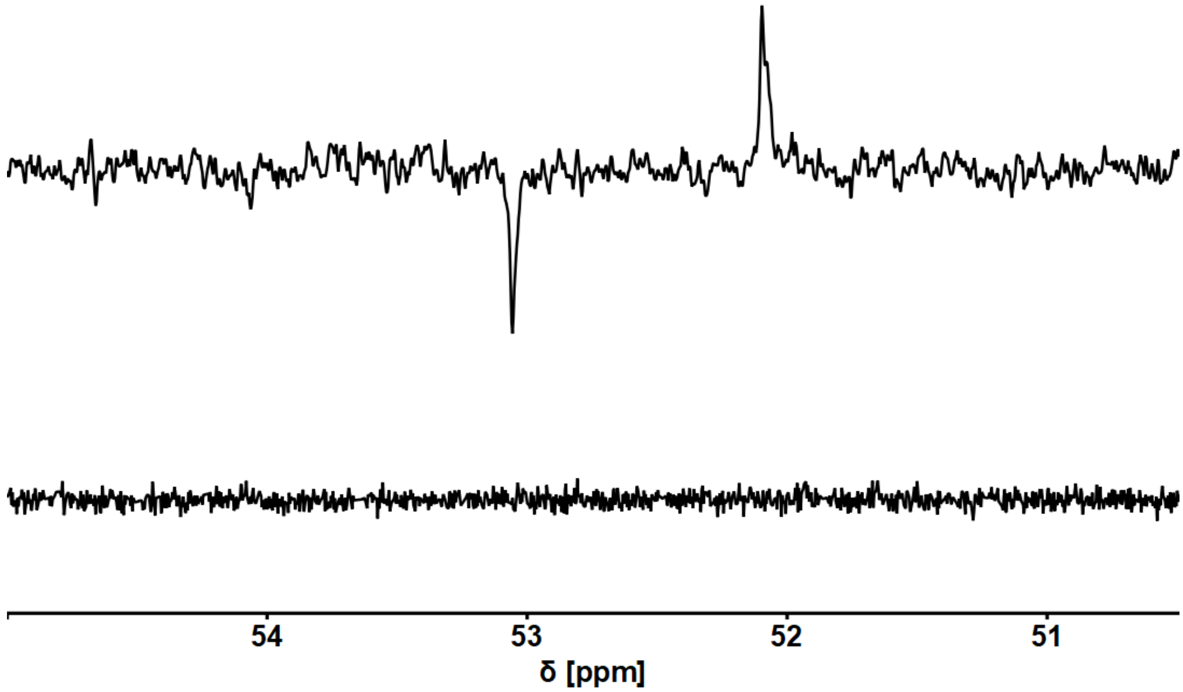

Manuscript Figure 4E – Panel #2

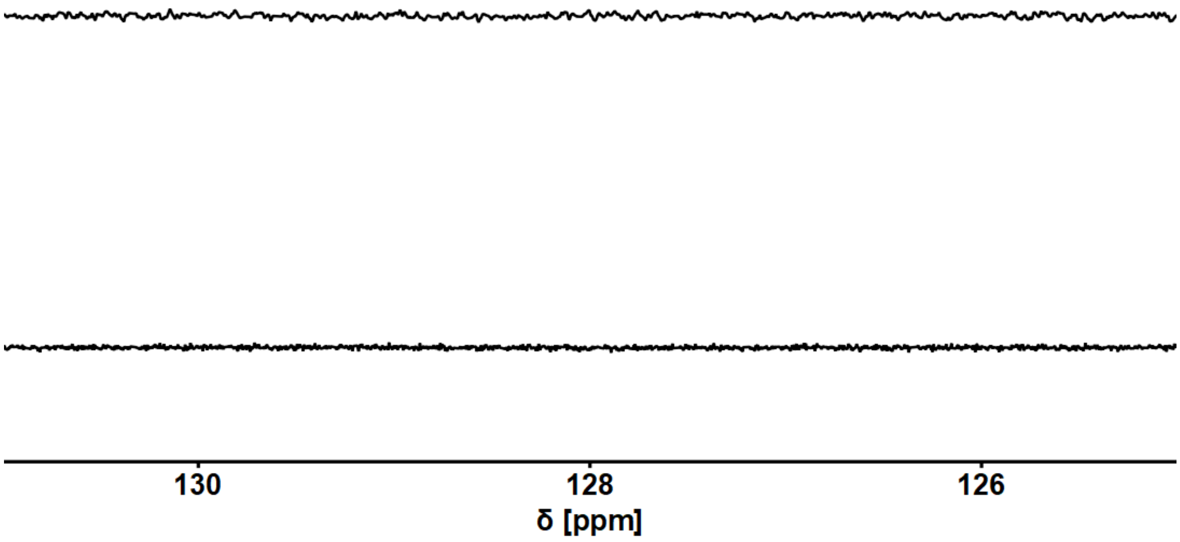

Manuscript Figure 4E – Panel #3

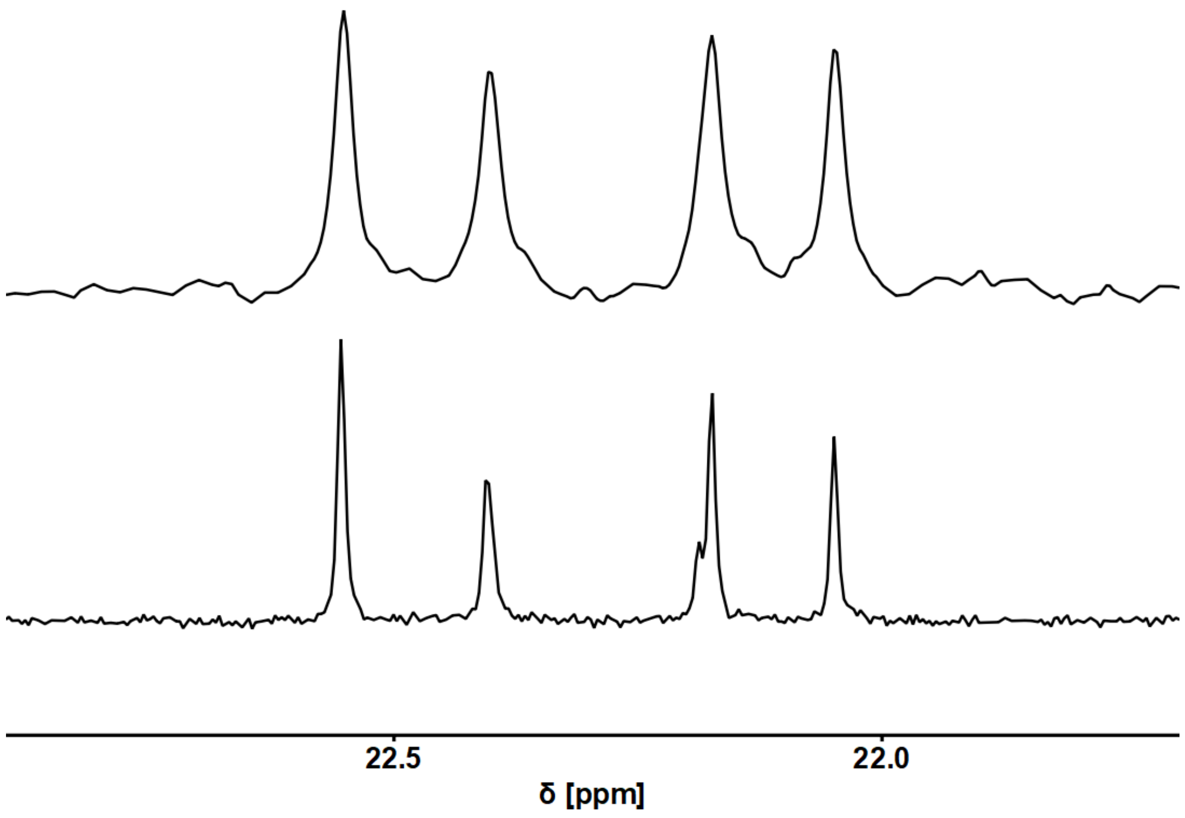

Manuscript Figure 4F – Panel #1

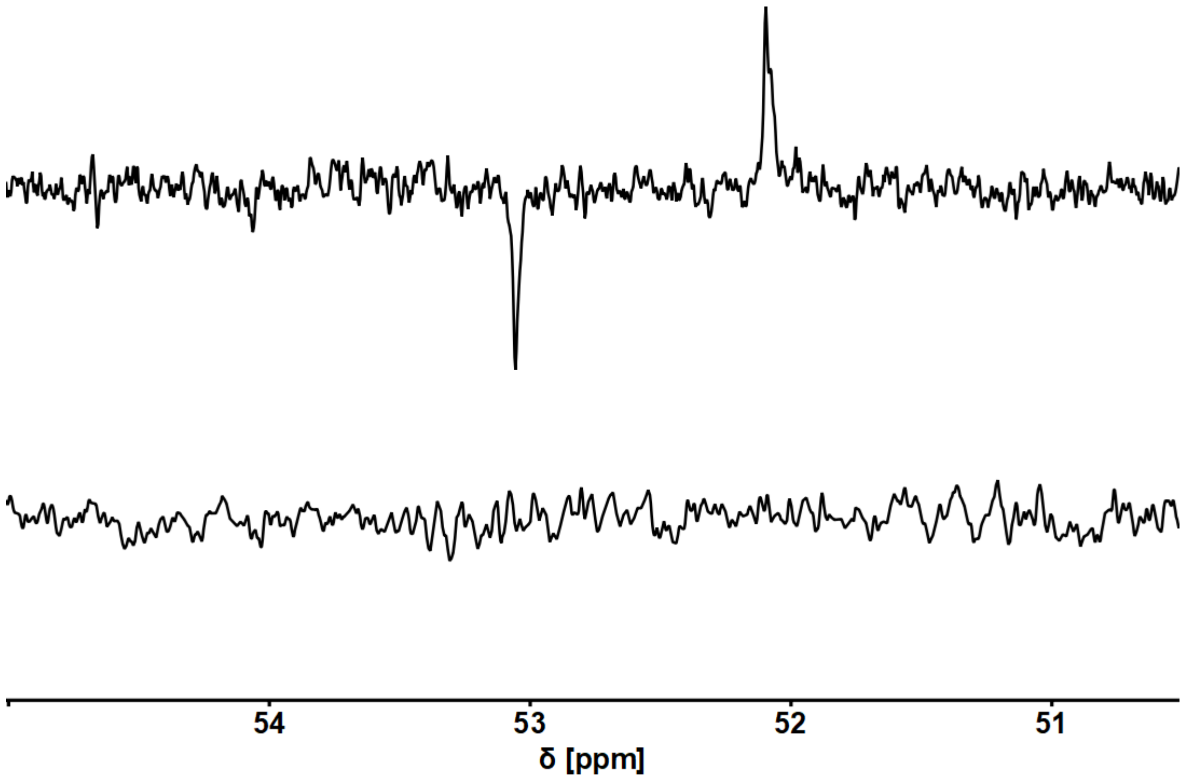

Manuscript Figure 4F – Panel #2

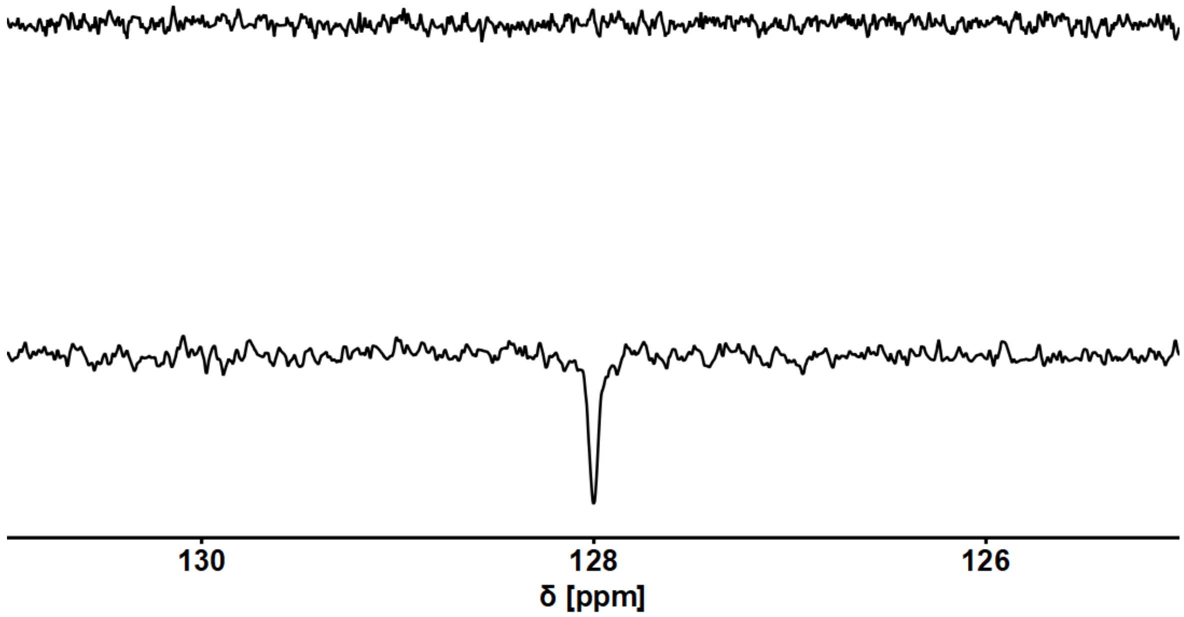

Manuscript Figure 4F – Panel #3

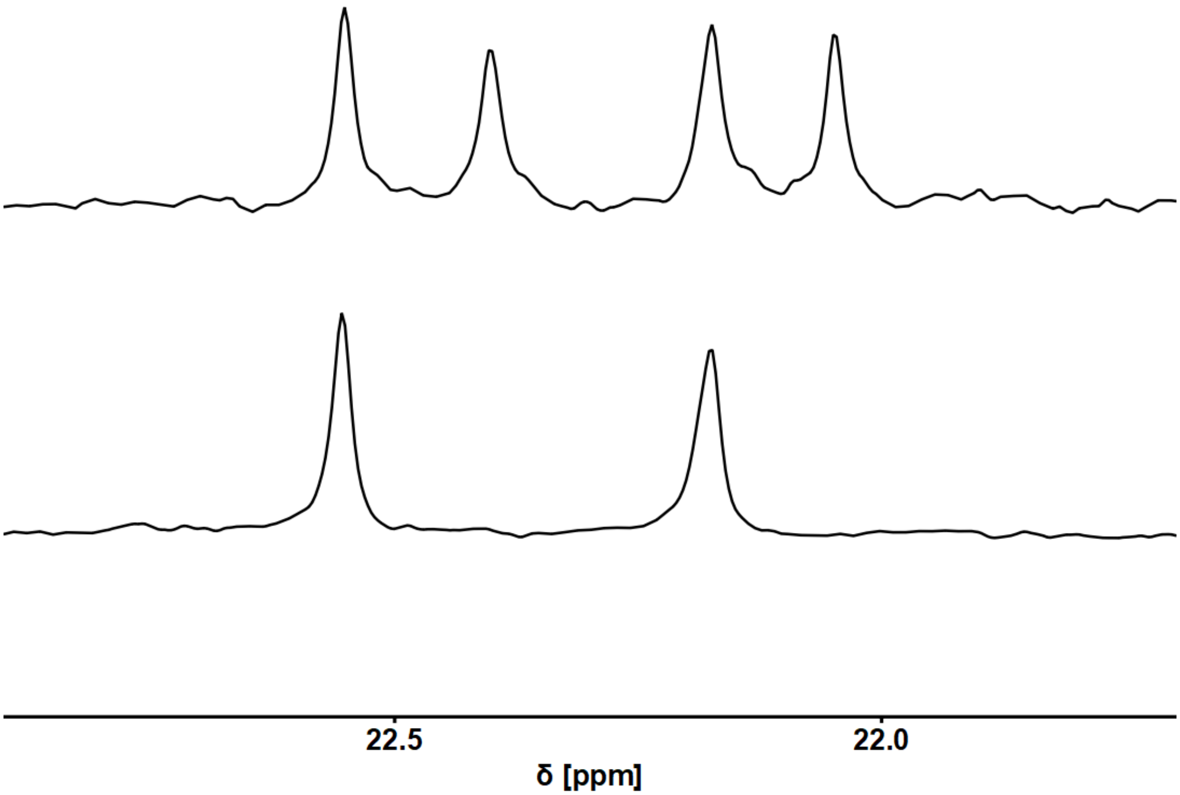

References

[21] D. Zabala, J. W. Cartwright, D. M. Roberts, B. J. C. Law, L. Song, M. Samborskyy, P. F. Leadlay, J. Micklefield, G. L. Challis, "A Flavin-Dependent Decarboxylase–Dehydrogenase–Monooxygenase Assembles the Warhead of α,β-Epoxyketone Proteasome Inhibitors." *J. Am. Chem. Soc.* **2016**, *138*, 4342–4345.

[24] A. Walter, W. Eisenreich, G. Storch, "Photochemical Desaturation and Epoxidation with Oxygen by Sequential Flavin Catalysis." *Angew. Chem. Int. Ed.* **2023**, *62*, e202310634.

[29] D. Bhattarai, M. J. Lee, A. Baek, I. J. Yeo, Z. Miller, Y. M. Baek, S. Lee, D.-E. Kim, J. T. Hong, K. B. Kim, "LMP2 Inhibitors as a Potential Treatment for Alzheimer's Disease." *J. Med. Chem.* **2020**, *63*, 3763–3783.

[39] O. Trott, A. J. Olson, "AutoDock Vina: improving the speed and accuracy of docking with a new scoring function, efficient optimization, and multithreading." *J. Comput. Chem.* **2010**, *31*, 455–461.

[40] J. Eberhardt, D. Santos-Martins, A. F. Tillack, S. Forli, "AutoDock Vina 1.2.0: New Docking Methods, Expanded Force Field, and Python Bindings." *J. Chem. Inf. Model.* **2021**, *61*, 3891–3898.

[44] X. Robert, P. Gouet, "Deciphering key features in protein structures with the new ENDscript server." *Nucleic Acids Res.* **2014**, *42*, W320–W324.

[45] Y.-M. Shi, M. Hirschmann, Y.-N. Shi, S. Ahmed, D. Abebew, N. J. Tobias, P. Grün, J. J. Crames, L. Pöschel, W. Kuttenlochner, C. Richter, J. Herrmann, R. Müller, A. Thanwisai, S. J. Pidot, T. P. Stinear, M. Groll, Y. Kim, H. B. Bode, "Global analysis of biosynthetic gene clusters reveals conserved and unique natural products in entomopathogenic nematode-symbiotic bacteria." *Nat. Chem.* **2022**, *14*, 701–712.

[46] F. Long, R. A. Nicholls, P. Emsley, S. Gražulis, A. Merkys, A. Vaitkus, G. N. Murshudov, "ACEDRG: A stereo-chemical description generator for ligands" *Acta Cryst.* **2017**, *D73*, 112–122.

[47] W. Kabsch, "XDS" *Acta Cryst. D* **2010**, *66*, 125–132.

[48] Collaborative, The CCP4 suite: programs for protein crystallography. *Acta Cryst. D* **1994**, *50*, 760–763.

[49] A. A. Vagin, R. A. Steiner, A. A. Lebedev, L. Potterton, S. McNicholas, F. Long, G. N. Murshudov, "REFMAC5 dictionary: organization of prior chemical knowledge and guidelines for its use." *Acta Cryst. D* **2004**, *60*, 2184–2195.

[50] P. Emsley, B. Lohkamp, W. G. Scott, K. Cowtan, "Features and development of Coot." *Acta Cryst. D* **2010**, *66*, 486–501.

[51] T. S. Mansour, C. A. Evans, "Decarboxylative Carbon Acylation of Malonates with Aminoacylimidazolides Mediated by Lewis Acids." *Synth. Commun.* **1990**, *20*, 773–781.

[52] G. R. Pettit, T. H. Smith, S. Feng, J. C. Knight, R. Tan, R. K. Pettit, P. Hinrichs, "Antineoplastic Agents. 561. Total Synthesis of Respirantin." *J. Nat. Prod.* **2007**, *70*, 1073–1083.

[53] C. Bérubé, A. Borgia, N. Voyer, "A novel route towards cycle-tail peptides using oxime resin: teaching an old dog a new trick." *Org. Biomol. Chem.* **2018**, *16*, 9117–9123.

[54] A. R. Hooper, A. Oštrek, A. Millan-Lopez, D. Sarlah, “Bioinspired Total Synthesis of Pyritide A2 through Pyridine Ring Synthesis.” *Angew. Chem. Int. Ed.* **2022**, *61*, e202212299.
