# Supplementary figures and images for: Studies of α′,β′‐Epoxyketone Synthesis by Small‐Molecule Flavins and Flavoenzymes
Source: Angew Chem Int Ed Engl. 2025 Oct 14;64(47):e202512568. doi: 10.1002/anie.202512568 (PMC12624316; doi:10.1002/anie.202512568)

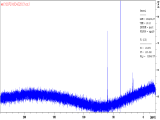

Supplement: Supplementary file 2 — Supporting Information [file ANIE-64-e202512568-s002.zip › Assay NMR Raw Data/Blank/WK-EPXF-Referenz/10/pdata/1/thumb.png]

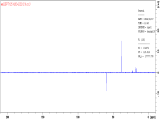

Supplement: Supplementary file 2 — Supporting Information [file ANIE-64-e202512568-s002.zip › Assay NMR Raw Data/Blank/WK-EPXF-Referenz/11/pdata/1/thumb.png]

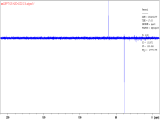

Supplement: Supplementary file 2 — Supporting Information [file ANIE-64-e202512568-s002.zip › Assay NMR Raw Data/Epoxomicin in Buffer/WK-Probe-Epoximicin-Ref/10/pdata/1/thumb.png]
