# Supplementary material for: Studies of α′,β′‐Epoxyketone Synthesis by Small‐Molecule Flavins and Flavoenzymes
Source: Angew Chem Int Ed Engl. 2025 Oct 14;64(47):e202512568. doi: 10.1002/anie.202512568 (PMC12624316; doi:10.1002/anie.202512568)
Supplement: Supplementary file 3 — Supporting Information [file ANIE-64-e202512568-s001.zip › NMR Data/NMR Raw Data/Compound 12 - Flavin - AW-21-FE-10-C2F2-cryo/11/LOGS-47306-2.html]

LOGS dataset LOGS-47306-2

If you are not redirected to LOGS, follow the link to dataset LOGS-47306-2
